# Intact but empty forests? Patterns of hunting-induced mammal defaunation in the tropics

**DOI:** 10.1371/journal.pbio.3000247

**Published:** 2019-05-14

**Authors:** Ana Benítez-López, Luca Santini, Aafke M. Schipper, Michela Busana, Mark A. J. Huijbregts

**Affiliations:** 1 Department of Environmental Science, Institute for Water and Wetland Research, Radboud University, Nijmegen, the Netherlands; 2 PBL, Netherlands Environmental Assessment Agency, The Hague, the Netherlands; Princeton University, UNITED STATES

## Abstract

Tropical forests are increasingly degraded by industrial logging, urbanization, agriculture, and infrastructure, with only 20% of the remaining area considered intact. However, this figure does not include other, more cryptic but pervasive forms of degradation, such as overhunting. Here, we quantified and mapped the spatial patterns of mammal defaunation in the tropics using a database of 3,281 mammal abundance declines from local hunting studies. We simultaneously accounted for population abundance declines and the probability of local extirpation of a population as a function of several predictors related to human accessibility to remote areas and species’ vulnerability to hunting. We estimated an average abundance decline of 13% across all tropical mammal species, with medium-sized species being reduced by >27% and large mammals by >40%. Mammal populations are predicted to be partially defaunated (i.e., declines of 10%–100%) in ca. 50% of the pantropical forest area (14 million km^2^), with large declines (>70%) in West Africa. According to our projections, 52% of the intact forests (IFs) and 62% of the wilderness areas (WAs) are partially devoid of large mammals, and hunting may affect mammal populations in 20% of protected areas (PAs) in the tropics, particularly in West and Central Africa and Southeast Asia. The pervasive effects of overhunting on tropical mammal populations may have profound ramifications for ecosystem functioning and the livelihoods of wild-meat-dependent communities, and underscore that forest coverage alone is not necessarily indicative of ecosystem intactness. We call for a systematic consideration of hunting effects in (large-scale) biodiversity assessments for more representative estimates of human-induced biodiversity loss.

## Abstract 2

This study estimates that roughly half of the tropics contain defaunated populations of medium and large mammal species as a result of hunting pressure; hunting effects are so pervasive that even intact forests and wilderness areas are becoming empty of mammal populations.

## Introduction

Tropical forests are increasingly degraded by industrial logging, urbanization, agriculture, and infrastructure [1, 2], with only 20% of the remaining area considered intact. Yet, this figure does not include other, more cryptic but pervasive forms of degradation, such as losses of wildlife due to overhunting [3]. Although humans in tropical areas have hunted for millennia to secure food and income, the current hunting rates are unsustainably high across the tropics due to the demand from growing human populations, an increasing commercialization of wild meat, and higher human accessibility to otherwise remote areas [4–8]. As a consequence, many tropical forests in the developing world are becoming “empty” (sensu Redford, 1992 [9]), with wildlife populations reduced or locally extirpated (i.e., defaunated), resulting in concomitant catastrophic consequences for ecosystems and the services and livelihoods that they provide [10]. Despite the increasing evidence of overhunting in the tropics, there is virtually no information about the spatial variation of hunting-induced defaunation and the areas where impacts might be most severe. Mapping “hotspots” of defaunation is crucial as a first step towards identifying and quantifying possible consequences for ecosystem functioning, as well as designing more targeted conservation measures.

Overhunting, as opposed to deforestation, is undetectable by remote-sensing techniques [11], and results from local studies have limited applicability to other areas [12–15]. However, upscaling local data with models based on quantitative relationships between impacts on wildlife populations and the main drivers of hunting pressure represents an unexplored approach to predict large-scale defaunation, particularly in understudied areas. Some of the main drivers of hunting include hunters’ accessibility to wildlife resources via road development and settlement establishment [3], hunters’ preferences for certain species [3, 10], and proximity to urban markets [3]. Additional factors are human population growth and subsequent increases in wild meat demand, socioeconomic status, food security, and governmental controls on hunting via law enforcement in protected areas (PAs) [16]. While the main drivers of such a multifaceted phenomenon were recently identified [3], the spatial pattern of hunting pressure on wildlife populations at the pantropical scale remains elusive.

Here, we model and project the spatial patterns of hunting-induced mammal defaunation in the tropics and identify areas where hunting impacts on mammal communities could be high. To this end, we developed a modelling framework based on a suite of important socioeconomic drivers of hunting pressure and taking into account the vulnerability of species to hunting [10]. Our models were based on the most extensive database to date on hunting impacts on mammal populations, consisting of 3,281 mammal abundance estimates in hunted and non-hunted areas extracted from 163 studies (S1 Table, https://figshare.com/projects/Intact_but_emtpy_forests_Patterns_of_hunting-induced_mammal_defaunation_in_the_tropics/31118). We related the abundance declines to socioeconomic drivers of hunting, including the distance to hunters’ access points, accessibility of urban markets, protection status (whether hunting occurred inside or outside PAs), human population densities, poverty levels, and access to domestic meat. Species body mass and diet were included as proxies of vulnerability to hunting [10] (S1 and S2 Figs). We modelled abundance declines with hurdle models to simultaneously incorporate the probability of being locally extirpated as well as abundance reductions (Methods). Subsequently, we used our models to map defaunation gradients across the tropics and quantify the magnitude and spatial extent of the population declines of 3,923 mammal species. We averaged the declines across species into a defaunation index (DI) ranging from 0 (intact mammal assemblage) to 1 (fully defaunated mammal assemblage). We conservatively consider areas with a DI >0.1 (more than 10% average reduction in mammal abundance across all species) to be partially defaunated (hereafter defaunated), and areas with DI >0.7 to be severely defaunated. We identified defaunation hotspots in areas where at least one third of the species had declines >70%. We overlaid our defaunation maps with intact forest (IF) [1] and human footprint (HF) [17] maps to assess the extent to which these pristine landscapes could be defaunated. Both initiatives indicate the location and extent of IFs, but do not consider the effect of hunting [18]. Finally, we assessed potential hunting-induced mammal abundance declines in PAs (PA, International Union for Conservation of Nature [IUCN] I–IV categories).

## Results and discussion

Distance to hunters’ access points, species body mass, and human population density (HPD) were the most important predictors of defaunation, followed by stunting and the protection status of the area (S1 Text, S5 Table, S3, S4, S5 Figs). More than half of the pantropical forest area (60%) is located within 10 km of the nearest human settlement, and >80% is within 20 km (S2 Fig). These are typical distances that hunters travel in the tropics [19], thereby suggesting that most of the tropical forest area is relatively accessible for hunters. Despite the complexity of the phenomena being modelled, the predictive performance of our models was satisfactory, with an overall sensitivity and specificity of 0.5 and 0.7, respectively, and an overall balanced accuracy of 0.6 for the different defaunation categories (S6B and S6C Fig). Pseudo-*R*^2^ values were 0.32 and 0.24 for the full and cross-validated models, respectively. Model performance was highest for the high defaunation level (DI > 0.7, S6B and S6C Fig); hence, the models are particularly useful to identify potential defaunation hotspots.

**Fig 1:**
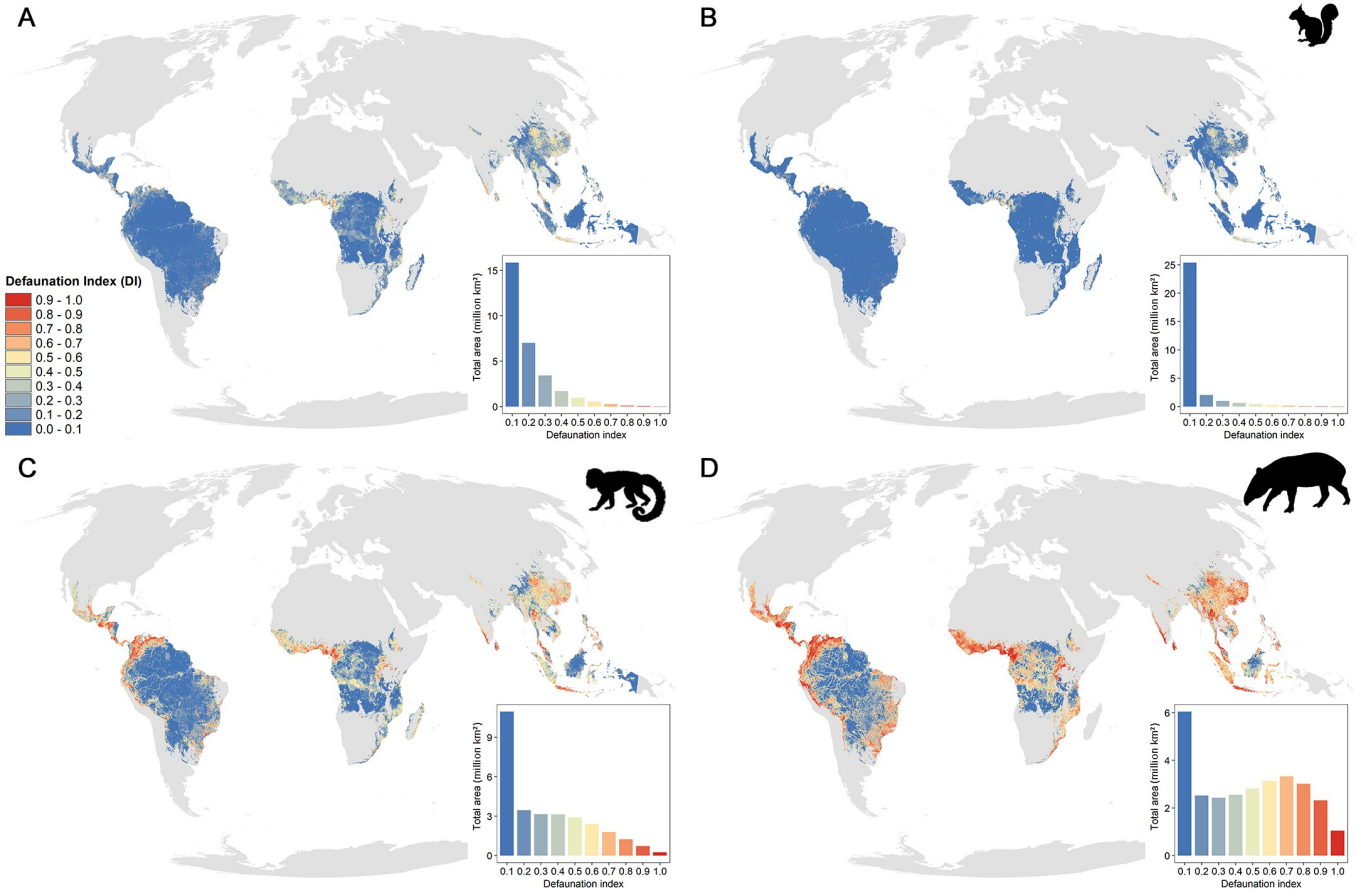
Geographic variation in hunting-induced defaunation for (A) all species, (B) small-sized species (<1 kg, e.g., *Sciurus* spp.), (C) medium-sized species (1–20 kg, e.g., *Allouatta* spp.), and (D) large-sized species (>20 kg, e.g., *Tapirus* spp.). The insets represent the total area (y-axis) under different levels of defaunation (x-axis, from DI = 0 [blue] to DI = 1 [red]). Note that the y-axes in the four insets have different scales. Available at https://figshare.com/projects/Intact_but_emtpy_forests_Patterns_of_hunting-induced_mammal_defaunation_in_the_tropics/31118. DI, defaunation index.

Across all mammal species and the entire pantropical area, we estimated an average DI of 0.13 ± 0.1 (mean ± SD, median: 0.09, IQR: 0.17, *N* = 30,004,854 grid cells) (Fig 1A). Results were highly similar if we removed areas outside the socioeconomic domain covered by our data (DI: 0.12 ± 0.1, median: 0.08, IQR: 0.16, *N* = 29,030,794 grid cells). For large mammals (>20 kg), we predicted an average decline of more than 40% across the tropics (DI: 0.42 ± 0.3, median: 0.43, IQR: 0.53, Fig 1D). An average reduction of 27% was predicted for medium-sized mammals (1–20 kg) (DI: 0.27 ± 0.2, median: 0.21, IQR: 0.42, Fig 1C), whereas impacts on small mammals (<1 kg) were negligible (DI: 0.05 ± 0.2, median: 0, IQR: 0.04; Fig 1B), reflecting that these are usually not hunted [20]. Our estimates are lower than the previously reported average decline of 83% [3], as here we are predicting hunting pressure in areas that are less hunted or intact, and for all mammals in the tropics, including a larger proportion of small species than in the original data set based on empirical studies (70% versus 20%).

We predict that approximately 47% of the pantropical forest area (ca. 14 million km^2^) is defaunated (DI > 0.1, Fig 1A), with mammal population declines of at least 50% in 3.5% (540 thousand km^2^) of the tropical forests. The average DI was highest in countries from West and Central Africa, particularly in The Gambia, Ghana, Togo, and Cameroon (DI range: 0.3–0.5, Fig 1A and S7 Fig), followed by some of the Asian countries (Thailand and Bangladesh). Nigeria, Burundi, Rwanda, Sri Lanka, and Java (Indonesia) also had high DIs, but these estimates were lower when we removed areas where local human population densities exceeded those covered in our data set (S8 Fig). For the rest of the tropics, our predictions were well within the range of observed values of the socioeconomic predictors (S8 Fig).

**Fig 2:**
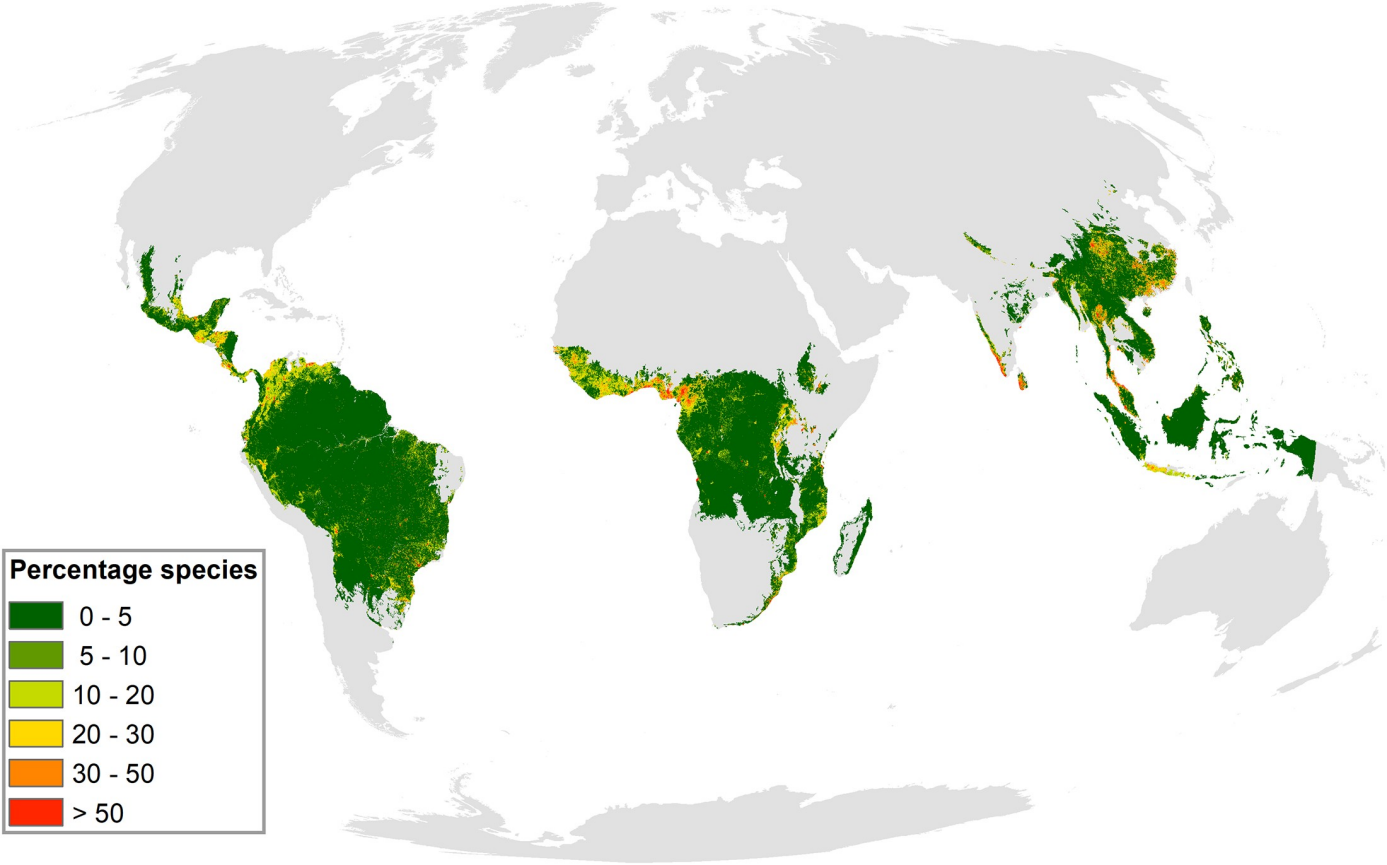
Percentage of mammal species per grid cell with DI >0.7 or abundance reductions of at least 70%. Colors range from green (low relative number of species with DI >0.7) to red (high relative number of species with DI >0.7). Orange to red areas showcase hotspots of hunting-induced mammal defaunation (with at least one third of the species with DI >0.7). Available at https://figshare.com/projects/Intact_but_emtpy_forests_Patterns_of_hunting-induced_mammal_defaunation_in_the_tropics/31118. DI, defaunation index.

We identified hotspots of hunting-induced defaunation in West and Central Africa (Cameroon, Guinea, and Cote D’Ivoire), Central America (Panama, Mexico, Costa Rica, Guatemala, and Honduras), Northwest South America (Colombia, Venezuela), and some areas in Southeast Asia (Thailand, Malaysia, and Southwest China, Fig 2). In the West African countries, particularly in Cameroon, more than 50% of the total number of species are predicted to have their populations reduced by 70%–100% because of hunting activities. Non-defaunated refugia (DI ≤ 0.1) were identified in the Guiana shield (Suriname, Guyana, and French Guiana) and the Brazilian Amazon, which represent inaccessible regions and sparsely populated areas (Fig 1A and S7 Fig).

Our maps with hunting-induced declines of medium- and large-sized mammals resemble spatial patterns of biomass harvest reported for Central Africa [21, 22], with high hunting pressure predicted in west, central, and north Cameroon, the Albertine Rift, south and north Democratic Republic of Congo (DRC), south Congo, and Gabon. Similarly to these earlier studies, we also identify hunting-free areas in central Congo, north Gabon, central DRC, and east Central African Republic (CAR). Furthermore, our defaunation maps for medium- and large-sized species suggest that high hunting pressure is expected along the Amazon River network and, particularly, in the east Amazon basin, which matches previous results on the spatial extent of hunting pressure for *Ateles* spp. (approximately 8.5 kg) [23].

**Fig 3:**
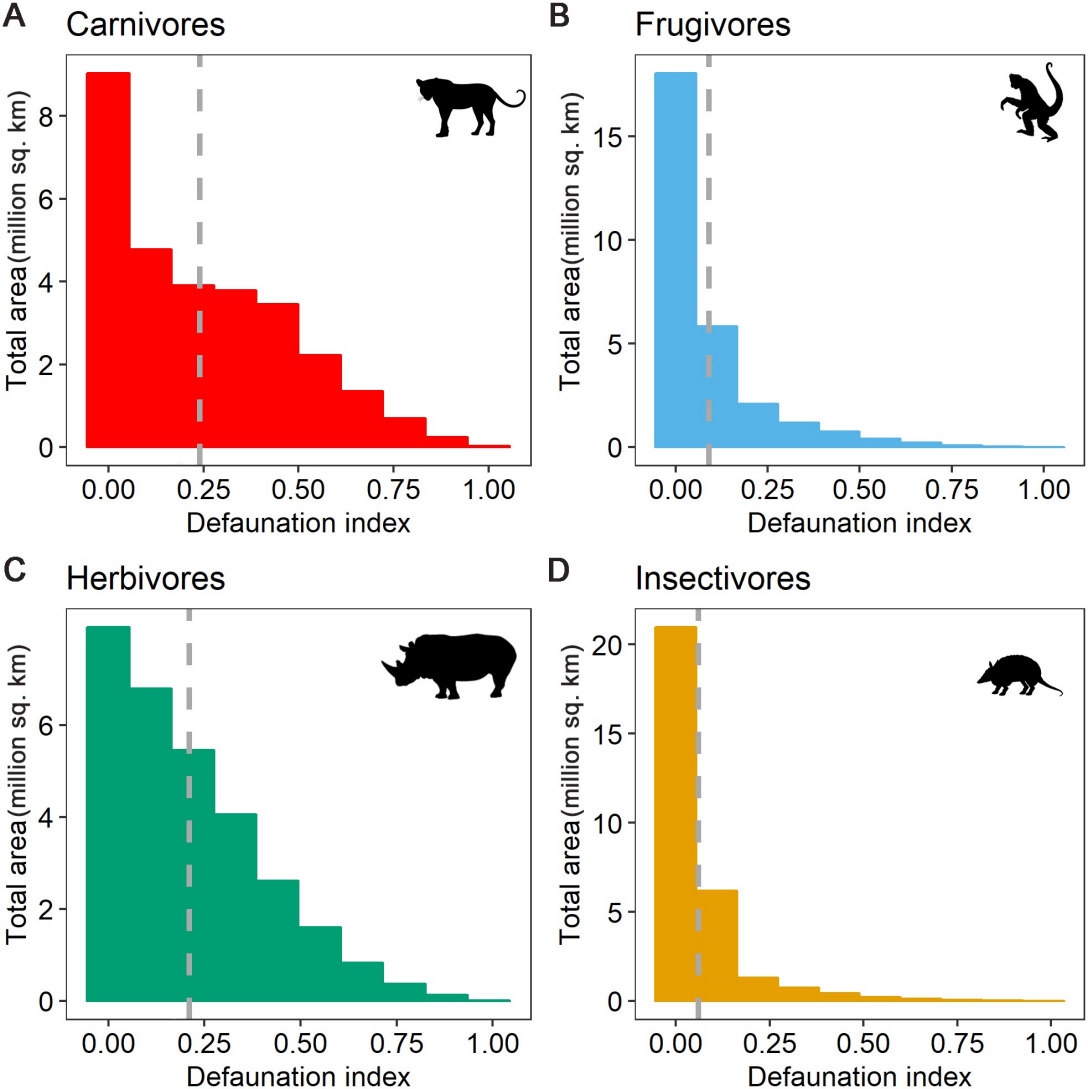
DI for different trophic groups: (A) carnivores, (B) herbivores, (C) frugivores, and (D) insectivores. The dashed gray line indicates the mean DI across the pantropical forest zone. The y-axes have different scales. Available at https://figshare.com/projects/Intact_but_emtpy_forests_Patterns_of_hunting-induced_mammal_defaunation_in_the_tropics/31118. DI, defaunation index; sq., square.

Our results are consistent with the idea that hunting is downsizing tropical mammal communities [3, 10]. The hunting-induced alteration of body size distributions across the tropics could trigger shifts in ecological functioning by impairing key ecological processes such as seed dispersal, predation, and herbivory, for which large-bodied species play a significantly more important role than smaller species [23–26]. We further estimated that declines were more severe for carnivores and herbivores (DI: 0.24 ± 0.2, median: 0.19, IQR: 0.37 and 0.22 ± 0.2, median: 0.17, IQR: 0.28, respectively) than for frugivores and insectivores (DI: 0.09 ± 0.1, median: 0.03, IQR: 0.1 and 0.06 ± 0.1, median: 0.02, IQR: 0.07, Fig 3). These results reflect differences in body mass distributions between feeding guilds (frugivores and insectivores are, on average, smaller than carnivores and herbivores), as well as differences in the spatial distribution and extent of the geographic ranges of the different guilds, with each species range encompassing diverse values of the socioeconomic drivers of hunting. Overall, the loss of large carnivores and herbivores may diminish top-down and bottom-up regulation, which can in turn trigger trophic cascades, resulting in the destabilization of tropical ecosystems and eventually leading to a net loss of diversity [27, 28].

**Fig 4:**
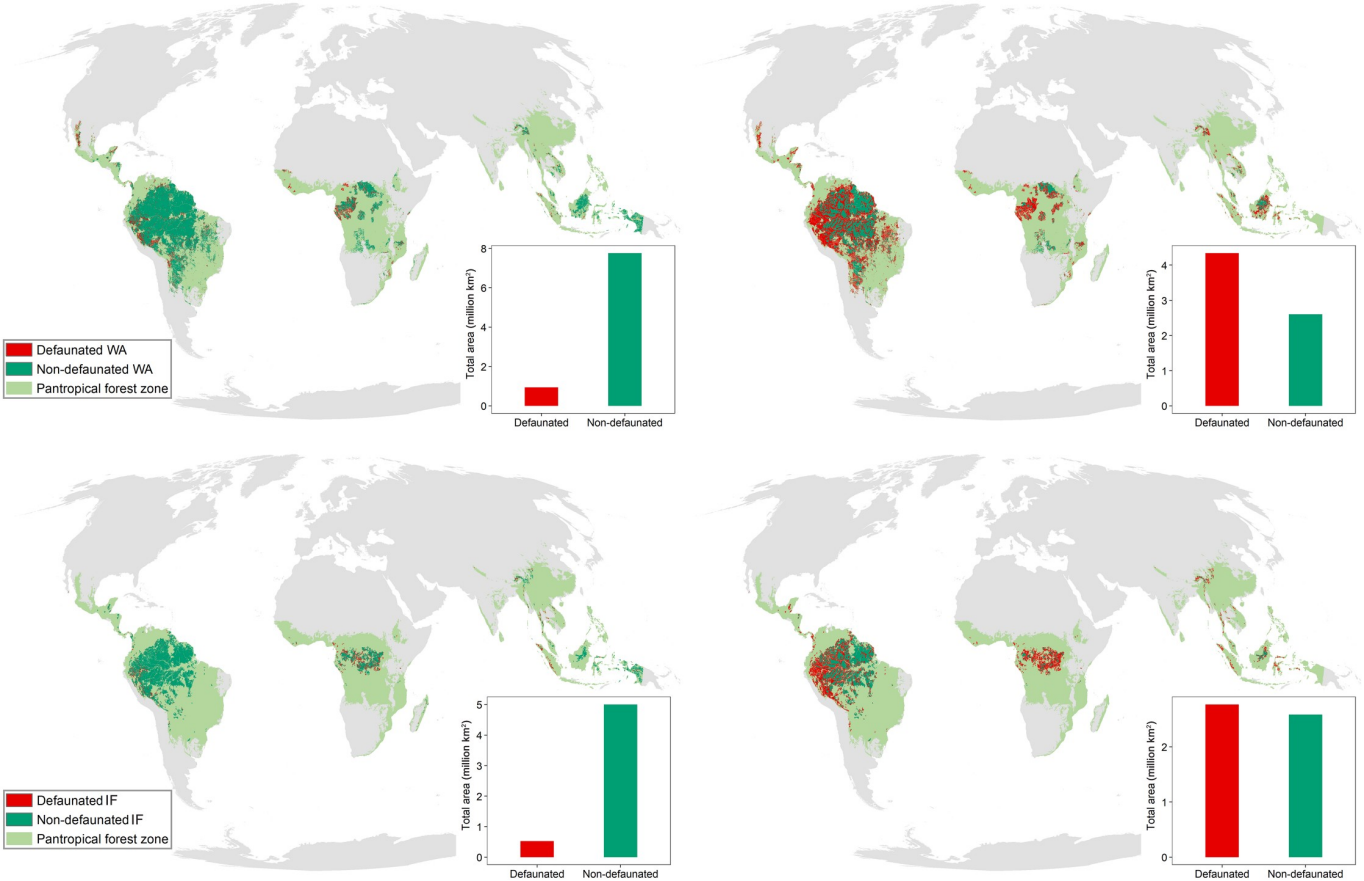
Geographic variation and spatial extent of hunting-induced defaunation for all species (left-hand side; A,C), and for large-bodied species (right-hand side; B,D) in WAs (A,B) and IFs (C,D). The insets represent the total area of forest (y-axis) predicted to be defaunated (DI > 0.1, red) and intact (DI ≤ 0.1, green) in the respective graphs. Available at https://figshare.com/projects/Intact_but_emtpy_forests_Patterns_of_hunting-induced_mammal_defaunation_in_the_tropics/31118. DI, defaunation index; IF, intact forest; WA, wilderness area.

We estimated that approximately 9% of IFs and 11% of wilderness area (WAs) are defaunated when full mammal assemblages are considered (Fig 4A and 4C). Large-bodied mammals could be, however, defaunated in more than half of the remaining IFs (52%, 2.8 million km^2^) and WAs (62%, 4.3 million km^2^), with barely any remaining intact areas in Central Africa (Fig 4B and 4D). Compared with degraded forests, IFs support more imperilled biodiversity and exceptional environmental values, including carbon sequestration and storage, water provision, indigenous cultures, and the maintenance of human health [18]. We now show here that even the last of the wild areas and the IF landscapes could be partly devoid of large mammal populations, potentially resulting in downsized mammal assemblages. As mammal complexity and diversity is reduced due to hunting, the net carbon storage of IFs could be compromised. For example, large frugivores are effective seed-dispersal agents, and their contribution to carbon storage in tropical forests is 2-fold: via discarded fruits that contribute to biomass accumulation in soil [29] and by being the main agent of dispersal of high-carbon large-seeded tree species [23]. Additionally, the loss of large herbivores results in lower herbivory rates in defaunated forests, which allows fast-growing herbivore-sensitive wind-dispersed plants (i.e., lianas) to outcompete slower-growing animal-dispersed trees. As lianas become more abundant, the net aboveground carbon uptake can be substantially reduced [30], thereby jeopardizing the role of IFs as carbon sinks [31].

Finally, we found that mammal populations could be defaunated in 20% of the IUCN PA when all mammal species are considered (S9 Fig), and that this pattern is more conspicuous in the case of large mammals (57% of the total PA). Most of the PAs predicted to be at risk of defaunation are located in Benin, Burundi, Bangladesh, Thailand, and India, with DI >0.3 (S10 Fig). While area-based conservation seems to be effective for conserving forest habitats, the evidence remains inconclusive regarding the effectiveness for maintaining wildlife populations [32–35], especially in the absence of anti-poaching regulations.

Our models conservatively assess the spatial extent of hunting, as potential time-lagged changes in mammal population abundances were not accounted for in the analysis. That is, we provide a snapshot of the spatial extent of defaunation by upscaling local studies performed between 1980 and 2017 to pantropical estimates. Hunting pressure, however, is sustained in time, and hunters will move towards more remote grounds once the larger and medium-sized species have disappeared in the proximity of their villages [36]. Thus, the mammal densities reported by local studies may be far from equilibrium and could have continued to decline years after the study was completed. In this sense, our estimates are relatively optimistic, yet they suggest a more dramatic picture of biodiversity loss than depicted in previous studies [2, 18], with possibly profound ramifications for ecosystem functioning and the livelihoods of wild-meat-dependent communities. Furthermore, we did not explicitly account for cultural beliefs and taboos regarding the consumption of some species in our analyses, as this information is extremely sparse in the literature. However, this is unlikely to have affected our conclusions because cultural beliefs and taboos do not prevent commercial hunting [37], and, in addition, their importance is declining due to rapid socioeconomic changes (i.e., increasing wealth and human mobility [38]). Finally, we did not consider that hunting impacts could be exacerbated by the synergies derived from habitat degradation, deforestation, and fragmentation [39]. Because hunter accessibility increases with increasing fragmentation via road development [40, 41], future projections of hunting-induced defaunation should rely on forecasts of HPD and include, when possible, the establishment of new roads that would provide access to the last remaining remote areas in the tropics.

We advocate for the inclusion of hunting-induced defaunation in large-scale biodiversity assessments, in which it has been routinely ignored due to data paucity. Other potential applications of our defaunation maps include species extinction risk assessments, conservation planning [42], the quantification of the effects of hunting on body size and animal biomass distributions [43], and progress evaluations to achieve global biodiversity targets [44]. For example, by combining our defaunation maps with spatially explicit mammal density estimates [45], we could gauge the remaining standing mammalian biomass in tropical forests. Special attention should be given to assess the integrity of those IFs and WAs that are at risk of defaunation, as their status could shift from “intact” to “half-empty” or “empty” forests. Retaining the integrity of intact tropical forests will not be possible if global and national environmental strategies do not address ongoing hunting practices.

## Methods

### Data collation

The relationship between mammal species abundance and hunting pressure was quantified using data from peer-reviewed and non-peer reviewed literature selected through a systematic literature search (S1 Table). We expanded upon the data set in Benítez-López and colleagues [3] that included studies that assessed the impact of hunting on wildlife abundance. Specifically, we searched for studies in which species abundance was reported in at least one hunted area and one unhunted control area, and at increasing distance from access points. Studies with potential confounding effects due to other disturbances (e.g., hunted and logged area versus unhunted unlogged area) were discarded (see Benítez-López and colleagues [3] for details on the search strategy and the study inclusion criteria). For updating the database, we specifically searched for studies performed in countries not included in the original database. In total, we included 163 studies covering 296 mammal species and 3,281 mammal abundance ratios, which corresponds to a 70% increase in the number of records in comparison with the original database of 1,938 ratios. Each study was georeferenced, and predictors were either recorded from the studies or extracted from spatially explicit raster maps (S1 Fig). Changes in abundance due to hunting pressure were expressed as the response ratio (RR) between the abundance of each species in hunted (*X*_*h*_) and unhunted (*X*_*c*_) sites within each study (RR = log(*X*_*h*_*/X*_*c*_)) [3]. Some ratios were zero for species that were completely extirpated in areas close to hunters’ access points (mean abundance equals zero), precluding log-transformation. Therefore, we converted our response variable into a binary variable (zero and nonzero abundance ratios) and a continuous variable (RRs calculated for abundance ratios >0) and modelled using a two-stage or hurdle model approach (see Modelling section). RRs are negative (RR < 0) or positive (RR > 0) if the abundance estimates are lower or higher, respectively, because of hunting pressure.

### Predictors

As predictors of hunting-induced defaunation, we used proximity to hunters’ access points (km), accessibility of urban markets (travel time to major towns, min), human population density (ind/km^2^), availability of domestic meat as an alternative food source (kg/km^2^), prevalence of stunting in children <5 years old (which indicates insufficient growth as a consequence of malnutrition and is an indicator of poverty [46]), literacy rate (as an indicator of access to qualified jobs), protection status (protected, nonprotected) [47], and species traits (body mass, kg, and diet—frugivores, herbivores, insectivores, omnivores, and carnivores) (S2 Table, S1 and S2 Figs). All spatially explicit predictors were calculated within the extent of present-day (sub-) tropical forest ecosystems (i.e., “forest zones”) based on the global year 2000 tree canopy cover data set [1, 48].

#### Distance to hunters’ access points

The distances to hunters’ access points (settlements, roads) were extracted from each study [3]. When the distance was not explicitly stated (e.g., comparison of close hunted area versus remote control area), we georeferenced the study locations and calculated the distance to the nearest settlement. Most of our studies (88%) referred to settlements as the main access point, and thus we generated a distance to the nearest settlement raster map for our model projections. We downloaded settlement locations for all countries in the tropical forest zone. The settlement data were collated from different sources, including national databases, the Humanitarian Data Exchange (HDE, https://data.humdata.org/), and OpenStreetMap (OSM, http://download.geofabrik.de) (S3 Table). Settlement data were visually inspected to assess country coverage and alignment with satellite imagery. When there were two or more data sets per country, we used the data set with the most extensive coverage. We merged data sets when they had similar coverages but differed at a few settlement points. Usually, the people that engage in hunting activities come from rural (and relatively) remote areas; i.e., decaying hunting pressure is usually related to the distance to small towns and villages, mostly because the livelihoods of people in urban areas do not depend on wild meat acquisition. Therefore, we excluded as access points settlements that referred to urban areas (those overlapping the built-up category in ESA Land Cover Maps for the year 2015 [https://www.esa-landcover-cci.org/] and those that were categorized as cities in OSM). We also filtered out location points that referred to the name of the country, county, region, or island (OSM data set). Subsequently, we calculated a raster map of the distance to the nearest settlement across the whole pantropical forest zone at a resolution of 30” (ca. 1 km; S2 Fig). Caribbean Islands, Oceanic Islands, and Papua New Guinea were discarded from the analyses due to the paucity of data on settlement locations.

#### Human population density and travel time to major towns

HPD is an indicator of wild meat demand and hunting pressure. We obtained raster maps of HPD (1-km resolution) for the period 1990–2015 from SEDAC [49, 50]. Per study location, estimates of HPD were extracted that matched the study year. Because urban areas may act as hubs where wild meat is commercialized in urban markets or transported elsewhere, we included travel time to major towns as a proxy for accessibility of urban markets and potential urban demand. Estimated travel times to the nearest town with more than 50,000 inhabitants were used as a proxy for accessibility of urban markets (1-km resolution, see [3]). Travel times were extracted from the accessibility map for the year 2000 [51] for studies performed before that year. For studies performed between 2000 and 2015, travel times were interpolated from accessibility maps for the years 2000 [51] and 2015 [52]. For more recent studies, the accessibility map from 2015 was used.

#### Stunting and literacy rate

As a poverty indicator, we used the prevalence of stunting, which represents insufficient child growth due to persistent dietary deficiencies and/or illness susceptibility. Stunting is considered a better indicator of economic and social deprivation than estimates of per capita income, as it indicates chronic failure to alleviate poverty [53]. To determine stunting, we used a spatial database on the prevalence of stunting in children <5 years old [46]. This database was outdated for some subnational areas, and thus we updated the spatial information with the WHO Global Database on Child Growth and Malnutrition (http://www.who.int/nutgrowthdb/about/en/), Demographic and Health Surveys (DHS), UNICEF MICS, and national surveys (e.g., ENSIN in Colombia), producing a new global map of stunting (S2 Fig). We recorded estimates for different time periods, and then we extracted stunting values per location by matching the values with the year of study (e.g., if the study year was 2007, we used stunting estimates corresponding to or close to 2007).

Education has been shown to correlate with the potential to access the labor market and, thus, alternative livelihoods that are less dependent on wild meat. We used literacy rate per country from the World Bank database (https://data.worldbank.org/) as a proxy for educational attainment.

#### Livestock biomass

We included livestock biomass as a proxy of accessibility of domestic meat as an alternative protein source to wild meat. We used global livestock density maps [54] to estimate the amount of domestic meat available per grid cell. The densities were transformed into livestock biomass per unit area (kg/km^2^) based on the average weights of cattle, sheep, pigs, and chicken extracted from the literature (S4 Table). The average weights were calculated based on a large number of animals (range: 1,531–4,951 individuals, depending on livestock type) across multiple studies (range: 24–37 studies).

### Modelling

Prior to modelling, we assessed the collinearity among the explanatory variables, which was low overall (S6 Fig). The highest correlation (Pearson’s rho = −0.59) was between stunting and literacy rate, and thus we kept the former, which is more intimately linked to poverty, for further analyses.

We used a hurdle or "two-stage" mixed model to accommodate the distribution of our response variable, which included local extirpations in ca. 14% of the cases (*N* = 408 out of 3,281) and abundance declines (or increases) compared with control areas in the rest of the data set (*N* = 2,873). Hurdle models use a binomial distribution to specify the probability of getting a 0 or a positive value, and then fit a zero-truncated probability density function to the nonzero data [55]. Hence, we used a binomial model to predict whether mammal populations were locally extinct or not (i.e., whether the relative abundance values were 0 or >0) and then fitted a Gaussian model through the nonzero RRs. We specified as random effects Country, Study, and Species to account for between-country variation in hunting laws and policies, culture, taboos, and traditions [56, 57] and to control for nonindependence in the data from the same study or species. All continuous variables included as fixed effects (see Predictors section) were standardized before model selection, and predictions were done with the models refit with unstandardized predictors. We fit models using maximum likelihood (ML) for model selection and restricted maximum likelihood (REML) for coefficient estimation [55]. Model selection was conducted for the binomial and continuous model based on the Bayesian Information Criterion (BIC), with models with a ΔBIC ≤2 considered supported and used for inference and for spatial predictions (S5 Table). We assessed the explained variance of the best models using the marginal *R*^2^ (fixed effects) and the conditional *R*^2^ (fixed and random effects) (see S1 Text). We also assessed variance partitioning among random effects and calculated semi-partial *R*^2^ values for each fixed effect included in the best binomial and continuous models (S5 Fig). We evaluated the predictive accuracy of the hurdle model with 5-fold cross-validation with an 80%/20% training/testing set. We split our predictions into three defaunation intensity categories of high (DI > 0.7), moderate (DI = 0.1–0.7), and low (DI ≤ 0.1), which roughly resemble the categories used in the Planetary Boundaries framework (Biodiversity Intactness Index [BII] = 90%, equivalent to DI = 0.1, and 90%–30%, equivalent to DI = 0.1–0.7) [58]. We assessed the accuracy of our model for predicting these categories of defaunation using sensitivity, specificity, and balanced accuracy. We also squared the correlation coefficient between the “Predicted” and the “Observed” data obtained from all repetitions of the 5-fold cross-validation to calculate a pseudo-*R*^2^, which was used as the predictive performance metric for the continuous range of observed abundance declines.

### Maps of hunting-induced defaunation

We extracted IUCN species ranges for all mammal species with distributions that overlapped the tropical forest area (*N* = 3,923 mammal species). This area was based on the global “forest zone” following Potapov and colleagues (2017) [1], who classified each grid cell from Landsat imagery (30-m resolution) with tree canopy cover greater than 20% in the year 2000 [48] as forest (S2A Fig). IUCN species ranges were gridded to match the spatial resolution of the predictors (1 km with Mollweide equal area projection), and, subsequently, we used our model to project the hunting-induced decline in abundance for each species. Our projections were based on the taxonomic identity of the species (captured by the random-effect intercept “Species”), the country where it was located (random-effect intercept “Country”), and its body mass (species vulnerability to hunting pressure), combined with the distribution of context-dependent drivers of hunting pressure (distance to settlements, population density, network of PAs, etc.) within the species range. Our empirical data cover 7.5% of all tropical mammal species and 30% of medium-sized and large-sized mammal species (i.e., those that are generally hunted [20]) included in the model projections. The number of species included in our database was proportional to the number of mammalian species included in the model projections (S11 Fig), and the extrapolations covered the range of body mass included in our data (range: 0.018–3,940 kg). The best represented taxa were elephants, armadillos, anteaters, and sloths, followed by ungulates, tapirs, primates, and carnivores. We back-transformed our predicted RR into a defaunation intensity index per species (DI_s_) as the reverse of the exp(RR), i.e., DI_s_ = 1 − exp(RR). Species-specific defaunation maps were then aggregated to create a composite map of hunting-induced defaunation by averaging the DI_s_ values across all species per grid cell ($DI=\frac{\sum_{1}^{s}{DI}_{s}}{S}$, with *S* being the number of species in a grid cell). We present our results in the form of defaunation gradients that range from 0 (not defaunated) to 1 (fully defaunated) [59], and consider areas with average DI > 0.1 as partially defaunated (hereafter, defaunated). We also calculated the proportion of species with DI_s_ > 0.7 to identify hotspots of defaunation caused by hunting. Because hunting is known to be a size-differential pressure [3, 10], we generated defaunation maps for small (<1 kg), medium (1–20 kg), and large (>20 kg) mammal species separately, in addition to an overall defaunation map. Additionally, we quantified defaunation for specific trophic groups (carnivores, herbivores, frugivores, insectivores) that play key roles in ecosystem functioning via seed dispersal, top-down, or bottom-up regulation [25, 28]. Finally, to identify geographic areas outside the range of socioeconomic predictor variables covered by our data, we calculated and mapped the multivariate environmental similarity surface (MESS) [60]. This analysis indicates areas where our DI estimates should be interpreted with caution, as they are based on extrapolation beyond the socioeconomic values used to fit our models (e.g., areas where HPD is higher or lower than the range of values included in the model fitting).

We then estimated the degree to which intact forest landscapes (IFLs) and WAs are defaunated (DI > 0.1) by overlapping our defaunation maps with the IFLs map, as defined by Potapov and colleagues (2017) [1], and the HF map, in which HF ≤ 2 is considered low and corresponds to WAs [2]. We calculated the total area that is defaunated (DI > 0.1) and intact (DI ≤ 0.1) within the WA and IF for all species and large mammal species. We used similar procedures to assess the risk of defaunation of IUCN PAs (cat. I–IV).

All analyses were conducted in R 3.4.1 [61]. The package “lme4” [62] was used to run the mixed models, “MuMIn” [63] was used for model selection and to calculate the marginal and conditional *R*^2^ of the models, “data.table” [64] was used to manipulate the large databases, “caret” [65] was used to calculate the accuracy metrics, “ModEvA” [66] was used to calculate the MESS, and “raster” [67] and “rgdal” [68] were used for GIS operations. Spatial analyses were conducted in Mollweide equal-area projection at a resolution of 1km using R and ArcGIS [69].

## Supporting information

S1 Text Model selection results. (DOCX)

S1 Fig Model scheme. Available at https://figshare.com/projects/Intact_but_emtpy_forests_Patterns_of_hunting-induced_mammal_defaunation_in_the_tropics/31118.
(TIF)

S2 Fig Spatial distribution of hunting studies and socioeconomic drivers of hunting pressure. (A) Location of 163 studies (in blue) with 3,281 abundance estimates for mammals in areas under hunting pressure. (B) Distance to the nearest rural settlement (km), (C) livestock biomass (kg/km^2^), (D) HPD (ind/km^2^), (E) travel time to major cities, (F) prevalence of stunting among children under five by the lowest available subnational administrative unit, varying years. Based primarily on the WHO Global Database on Child Growth and Malnutrition (http://www.who.int/nutgrowthdb/about/en/). Available at https://figshare.com/projects/Intact_but_emtpy_forests_Patterns_of_hunting-induced_mammal_defaunation_in_the_tropics/31118. HPD, human population density.
(TIF)

S3 Fig Partial plots of the relationships between the probability of a species/population being locally extirpated (0) or not (1) due to hunting, and the retained predictors in the best model. (A) Distance to hunters’ access points, (B) HPD, (C) PA status (yes, no), (D) body mass, and (E) prevalence of stunting. CIs (95%) are shown in gray. The scale of the y-axis has been adjusted to enhance visualization of the fitted lines. Available at https://figshare.com/projects/Intact_but_emtpy_forests_Patterns_of_hunting-induced_mammal_defaunation_in_the_tropics/31118. HPD, human population density; PA, protected area.
(TIF)

S4 Fig Partials plots of the relationships between the RR (change in species abundance) and the retained predictors in the best model. The dashed gray line indicates that hunting pressure has no effect on species abundance (RR = 0). Positive values indicate an increase in species abundance, whereas negative values indicate a negative effect on species abundance. (A) Distance to hunters’ access points, (B) body mass, (C) interaction between body mass and distance, and (D) HPD. CIs (95%) are shown in gray. In (C), dark blue: 0.1 kg, e.g., *Oryzomys* spp.; light blue: 1 kg, e.g., *Sylvilagus brasiliensis*; yellow: 10 kg, e.g., *Alouatta* spp.; orange: 100 kg, e.g., *Panthera onca*; red: 4,000 kg, e.g., *Loxodonta africana*. Available at https://figshare.com/projects/Intact_but_emtpy_forests_Patterns_of_hunting-induced_mammal_defaunation_in_the_tropics/31118. HPD, human population density; RR, response ratio.
(TIF)

S5 Fig Effects and relative importance of predictors on mammal abundance declines due to hunting pressure. Standardized coefficient estimates of the variables retained in the best (A) binomial (extinct/no extinct) and (B) Gaussian models (RR). Explained variance by (C) the random effects and the (D) fixed effects of the binomial and Gaussian models. Available at https://figshare.com/projects/Intact_but_emtpy_forests_Patterns_of_hunting-induced_mammal_defaunation_in_the_tropics/31118. BM, body mass; Dist, distance to hunters’ access points; HPD, human population density; PA, protected area; RR, response ratio; Stunt, stunting.
(TIF)

S6 Fig Collinearity test between explanatory variables and predictive performance of the models. (A) Correlation plot between explanatory variables, (B) predictive performance metrics (mean ± SD) for three categories of defaunation (low, DI < 0.1; intermediate, DI = 0.1–0.7; high, DI = 0.7–1.0). (C) Predicted versus observed categories of defaunation intensity obtained with the best hurdle model for the cross-validated data set. Size of the squares relative to the size of the grid indicates the proportion of the observed data of a given DI category (columns) to match with the prediction of a particular DI category (rows). Available at https://figshare.com/projects/Intact_but_emtpy_forests_Patterns_of_hunting-induced_mammal_defaunation_in_the_tropics/31118. BM, body mass, DI, defaunation index; Dist, distance to hunters’ access points; HPD, human population density; Literacy, literacy rate; LivestockBio, biomass of domestic livestock; Stunt, stunting; TravTime, travel time to major towns.
(TIF)

S7 Fig Mean DI per country and 95% CI (black lines) for (A) all pantropical area and (B) after excluding areas outside the socioeconomic domain covered by our data. Colors denote different regions. Available at https://figshare.com/projects/Intact_but_emtpy_forests_Patterns_of_hunting-induced_mammal_defaunation_in_the_tropics/31118. CAR, Central African Republic; DI, defaunation index; DRC, Democratic Republic of Congo.
(TIF)

S8 Fig (A) Geographic areas inside and outside the socioeconomic domain covered by our data, as estimated by the MESS. The values represent the similarity between each grid cell in pantropical range and those in the reference data set used to fit the models. Values range from positive (green) to negative (red). Positive values represent interpolation areas with similar socioeconomic factors (distance to hunters’ access points, HPD, and prevalence of stunting) than those used to fit the models that are covered by our data set. Negative values indicate localities where at least one socioeconomic variable is outside the range of socioeconomic variables in our data set. (B) Main variable that is dissimilar in each grid cell compared with the socioeconomic domain in our data set. Orange, distance to the nearest rural settlement; green, HPD; blue, prevalence of stunting. Available at https://figshare.com/projects/Intact_but_emtpy_forests_Patterns_of_hunting-induced_mammal_defaunation_in_the_tropics/31118. HPD, human population density; MESS, multivariate environmental similarity surface.
(TIF)

S9 Fig Geographic variation and spatial extent of hunting-induced defaunation in IUCN PAs (I–IV) for (A) all species and (B) large-bodied species. Available at https://figshare.com/projects/Intact_but_emtpy_forests_Patterns_of_hunting-induced_mammal_defaunation_in_the_tropics/31118. IUCN, International Union for Conservation of Nature; PA, protected area.
(TIF)

S10 Fig Mean DI and 95% CI (black lines) within IUCN PAs (I–IV) per country. Colors denote different regions. Available at https://figshare.com/projects/Intact_but_emtpy_forests_Patterns_of_hunting-induced_mammal_defaunation_in_the_tropics/31118. CAR, Central African Republic; DI, defaunation index; DRC, Democratic Republic of Congo; IUCN, International Union for Conservation of Nature; PA, protected area.
(TIF)

S11 Fig The relationship between the number of species represented in our database (*N* = 296) and the number of tropical species for which we extrapolated our models (*N* = 3,293) for 14 orders. Lines show 10% (dotted), 50% (solid), and 90% (dashed) representations of the predicted species in our data set. Available at https://figshare.com/projects/Intact_but_emtpy_forests_Patterns_of_hunting-induced_mammal_defaunation_in_the_tropics/31118.
(TIF)

S1 Table List of data sources included in our analyses and the associated metadata: Author and year, type of source (SP, MT, DT, TR, BC), location, habitat, order, type of access point, type of hunting, legality status, number of studies, and methods used in each source. BC, book chapter; DT, doctoral thesis; MT, master thesis; SP, scientific publication; TR, technical report.
(DOCX)

S2 Table Overview of explanatory variables included in the hurdle models. (DOCX)

S3 Table Sources of settlement location data. (DOCX)

S4 Table Number of animals and studies used to estimate average body weights for cattle, sheep, goats, pigs, and chickens. (DOCX)

S5 Table Model selection results for (a) the binomial model (0/1, extirpated versus not extirpated) and (b) the continuous model. Models were ranked according to BIC. We only show models with a BIC weight >0.01. The best model (ΔBIC < 2, in bold) was used in the cross-validation analyses and for spatial predictions. BM, body mass; BIC, Bayesian Information Criterion; Dist, distance to hunters’ access points, PA, protected area; PopDens, human population density; Stunt, stunting; TravTime, travel time to major towns.
(DOCX)

## Abbreviations

BIC: Bayesian Information Criterion
BII: Biodiversity Intactness Index
CAR: Central African Republic
DHS: Demographic and Health Surveys
DI: defaunation index
DRC: Democratic Republic of Congo
HDE: Humanitarian Data Exchange
HF: human footprint
HPD: human population density
IF: intact forest
IFL: intact forest landscape
IUCN: International Union for Conservation of Nature
MESS: multivariate environmental similarity surface
ML: maximum likelihood
OSM: OpenStreetMap
PA: protected area
REML: restricted maximum likelihood
RR: response ratio
WA: wilderness area
