# Supplementary material for: Intact but empty forests? Patterns of hunting-induced mammal defaunation in the tropics
Source: PLoS Biol. 2019 May 14;17(5):e3000247. doi: 10.1371/journal.pbio.3000247 (PMC6516652; doi:10.1371/journal.pbio.3000247)
Supplement: S1 Text — (DOCX) [file pbio.3000247.s001.docx]

S1 Text. Model selection results

We identified one best competing model for each of the binomial and continuous models (S5 Table). Marginal and conditional R^2^ were 0.31 and 0.71, and 0.10 and 0.32 for the binomial and continuous models, respectively. The binomial and continuous models retained similar predictors with slight differences regarding non-linearity in the relationships and the inclusion of an interaction term in the continuous model (S4 Table). Among the random effects, “Species” and “Study” accounted for most of the variation both in the binomial and in the continuous model, with “Country” contributing substantially less (S4 Fig). Among the fixed effects, distance (linear and quadratic terms) and body mass were the most important explanatory variables (S4 Fig). The binomial model included the effects of body mass, distance (quadratic), stunting and human population density (HPD), whereas the continuous model included the effects of body mass, distance (quadratic), the interaction Dist x BM and HPD (quadratic) (S4 Table). Overall the probability that a mammal species was not locally extirpated increased asymptotically with the distance to hunters’ access points and linearly with the prevalence of stunting, indicating that poverty is not directly linked to hunting-induced mammal extinctions (S5 Fig). In turn, the probability of not being locally extirpated decreased in highly populated locations and outside protected areas, particularly for small-bodied species. Among species that were not locally extinct, medium- and large-bodied species ( >1kg) had lower abundance in the proximity of hunters’ access points, whereas smaller species maintained similar or higher population densities regardless the hunting pressure (S6 Fig). Furthermore, human population density was negatively related with mammal population densities.
