# Supplementary figures and images for: Intact but empty forests? Patterns of hunting-induced mammal defaunation in the tropics
Source: PLoS Biol. 2019 May 14;17(5):e3000247. doi: 10.1371/journal.pbio.3000247 (PMC6516652; doi:10.1371/journal.pbio.3000247)

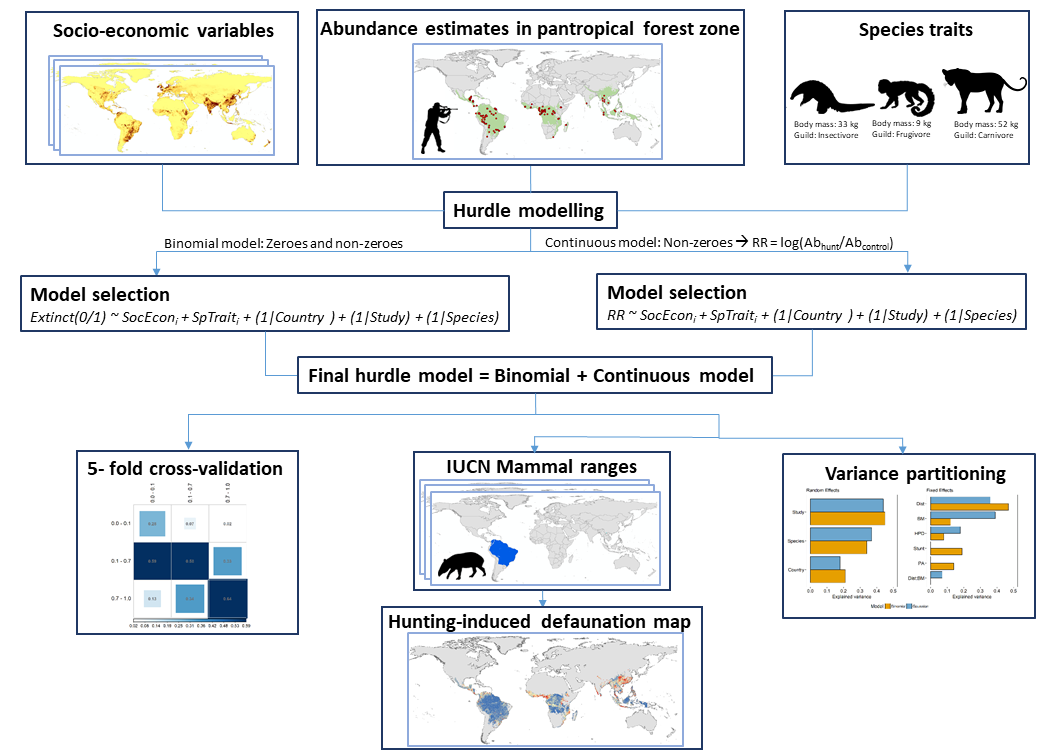

Supplement: S1 Fig — Available at https://figshare.com/projects/Intact_but_emtpy_forests_Patterns_of_hunting-induced_mammal_defaunation_in_the_tropics/31118. (TIF) [file pbio.3000247.s002.tif]

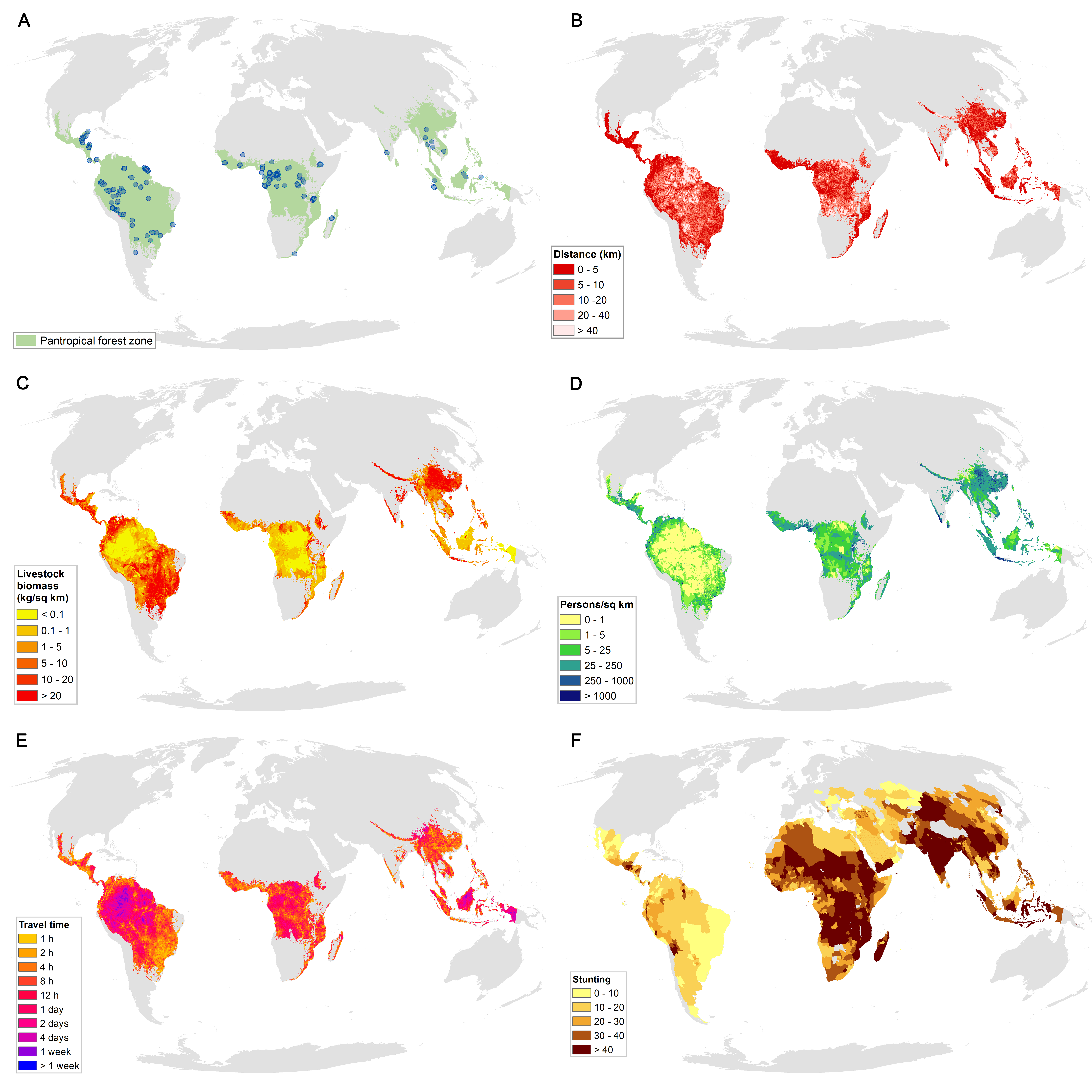

Supplement: S2 Fig — (A) Location of 163 studies (in blue) with 3,281 abundance estimates for mammals in areas under hunting pressure. (B) Distance to the nearest rural settlement (km), (C) livestock biomass (kg/km2), (D) HPD (ind/km2), (E) travel time to major cities, (F) prevalence of stunting among children under five by the lowest available subnational administrative unit, varying years. Based primarily on the WHO Global Database on Child Growth and Malnutrition (http://www.who.int/nutgrowthdb/about/en/). Available at https://figshare.com/projects/Intact_but_emtpy_forests_Patterns_of_hunting-induced_mammal_defaunation_in_the_tropics/31118. HPD, human population density. (TIF) [file pbio.3000247.s003.tif]

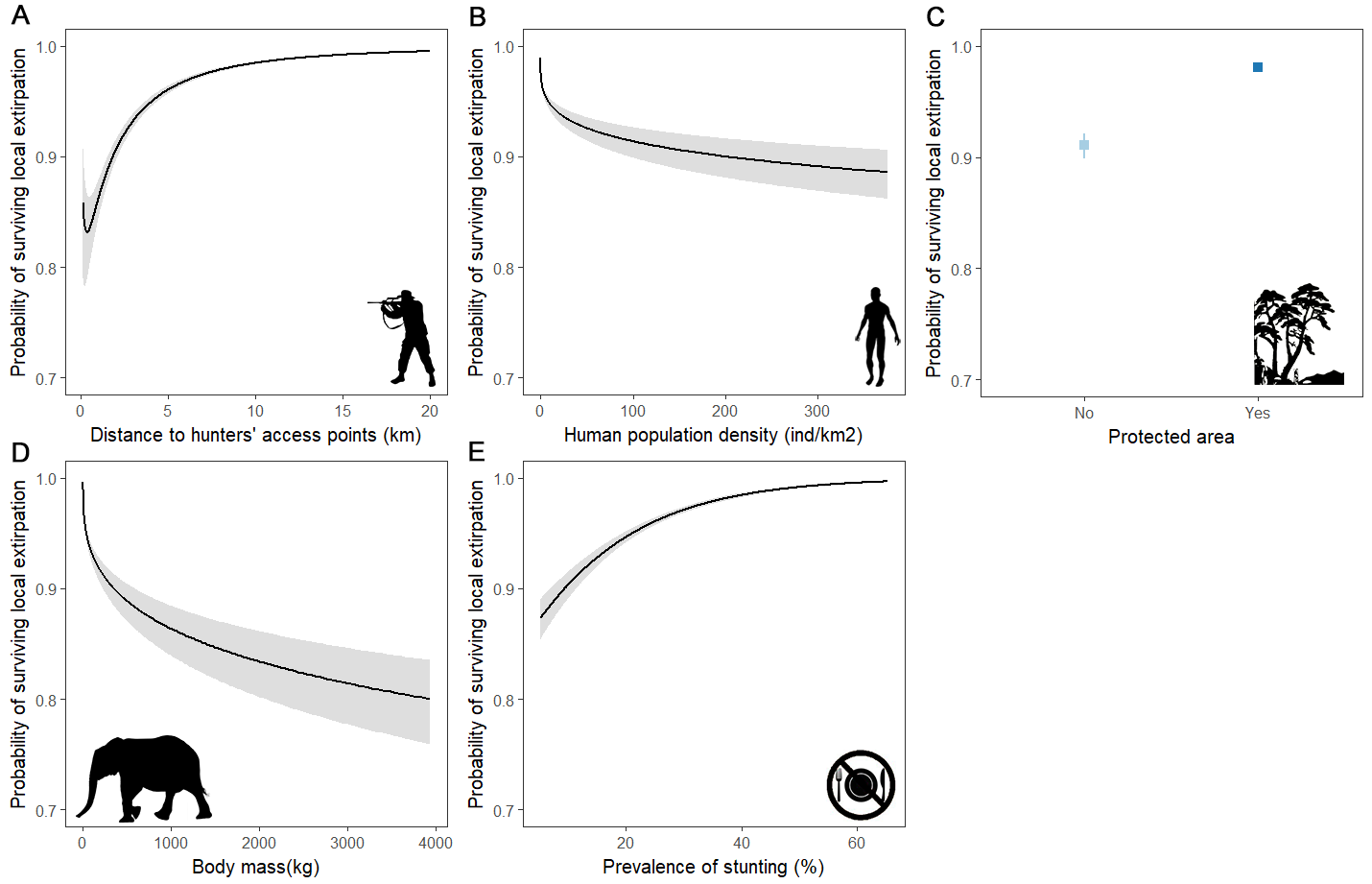

Supplement: S3 Fig — (A) Distance to hunters’ access points, (B) HPD, (C) PA status (yes, no), (D) body mass, and (E) prevalence of stunting. CIs (95%) are shown in gray. The scale of the y-axis has been adjusted to enhance visualization of the fitted lines. Available at https://figshare.com/projects/Intact_but_emtpy_forests_Patterns_of_hunting-induced_mammal_defaunation_in_the_tropics/31118. HPD, human population density; PA, protected area. (TIF) [file pbio.3000247.s004.tif]

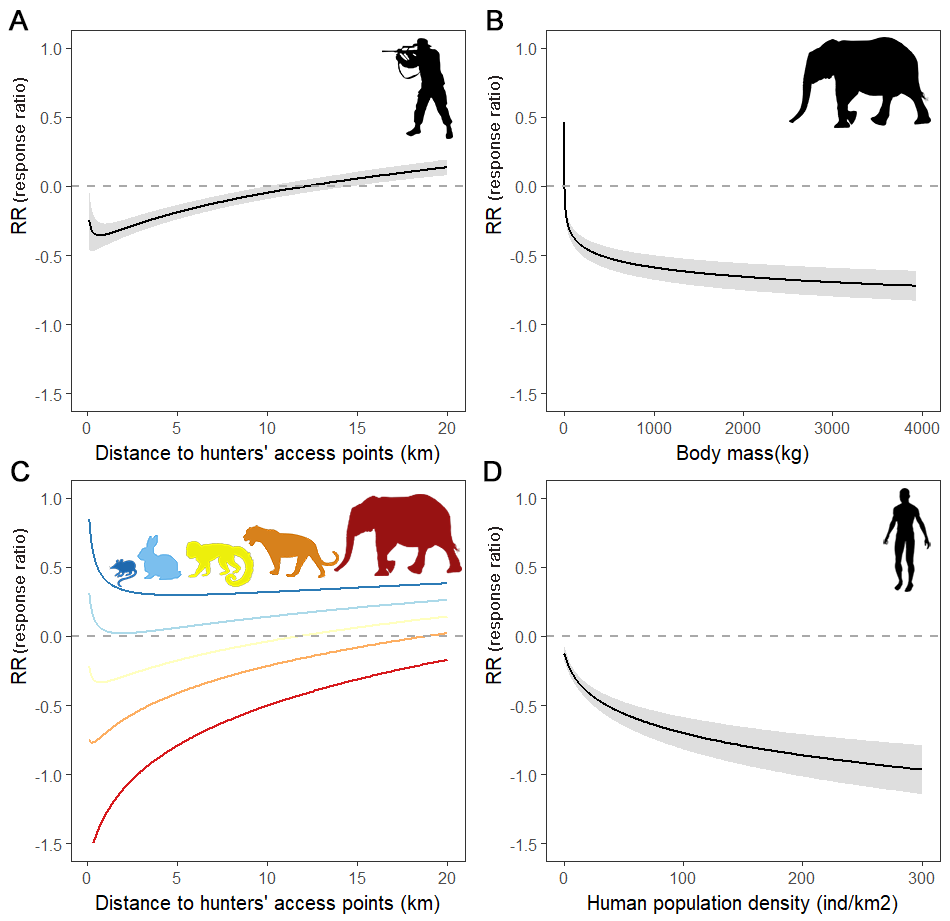

Supplement: S4 Fig — The dashed gray line indicates that hunting pressure has no effect on species abundance (RR = 0). Positive values indicate an increase in species abundance, whereas negative values indicate a negative effect on species abundance. (A) Distance to hunters’ access points, (B) body mass, (C) interaction between body mass and distance, and (D) HPD. CIs (95%) are shown in gray. In (C), dark blue: 0.1 kg, e.g., Oryzomys spp.; light blue: 1 kg, e.g., Sylvilagus brasiliensis; yellow: 10 kg, e.g., Alouatta spp.; orange: 100 kg, e.g., Panthera onca; red: 4,000 kg, e.g., Loxodonta africana. Available at https://figshare.com/projects/Intact_but_emtpy_forests_Patterns_of_hunting-induced_mammal_defaunation_in_the_tropics/31118. HPD, human population density; RR, response ratio. (TIF) [file pbio.3000247.s005.tif]

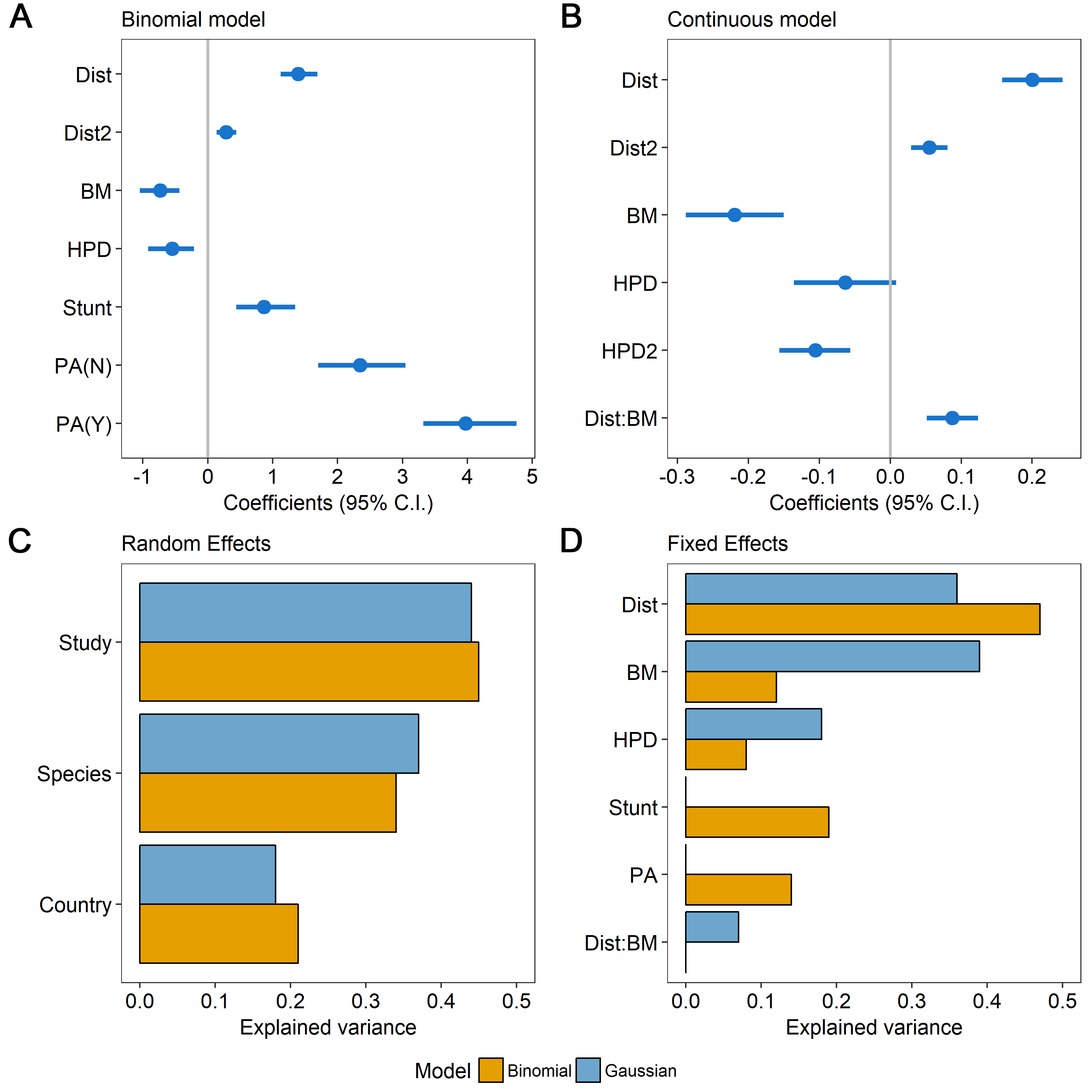

Supplement: S5 Fig — Standardized coefficient estimates of the variables retained in the best (A) binomial (extinct/no extinct) and (B) Gaussian models (RR). Explained variance by (C) the random effects and the (D) fixed effects of the binomial and Gaussian models. Available at https://figshare.com/projects/Intact_but_emtpy_forests_Patterns_of_hunting-induced_mammal_defaunation_in_the_tropics/31118. BM, body mass; Dist, distance to hunters’ access points; HPD, human population density; PA, protected area; RR, response ratio; Stunt, stunting. (TIF) [file pbio.3000247.s006.tif]

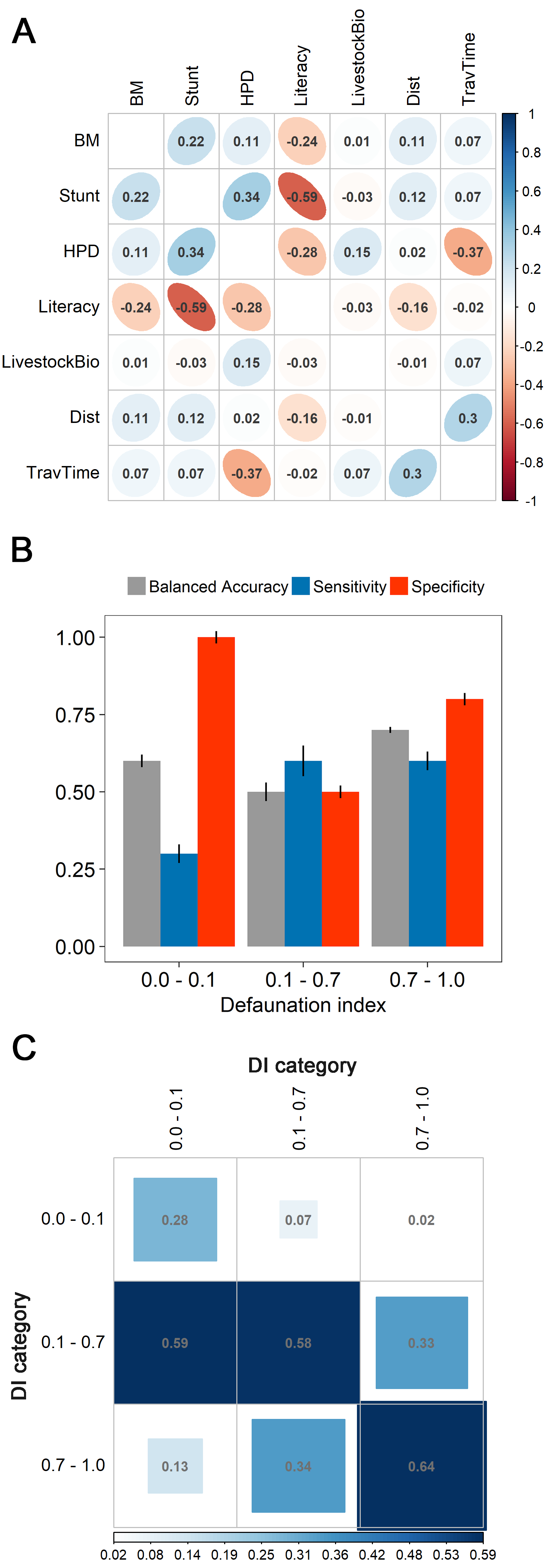

Supplement: S6 Fig — (A) Correlation plot between explanatory variables, (B) predictive performance metrics (mean ± SD) for three categories of defaunation (low, DI < 0.1; intermediate, DI = 0.1–0.7; high, DI = 0.7–1.0). (C) Predicted versus observed categories of defaunation intensity obtained with the best hurdle model for the cross-validated data set. Size of the squares relative to the size of the grid indicates the proportion of the observed data of a given DI category (columns) to match with the prediction of a particular DI category (rows). Available at https://figshare.com/projects/Intact_but_emtpy_forests_Patterns_of_hunting-induced_mammal_defaunation_in_the_tropics/31118. BM, body mass, DI, defaunation index; Dist, distance to hunters’ access points; HPD, human population density; Literacy, literacy rate; LivestockBio, biomass of domestic livestock; Stunt, stunting; TravTime, travel time to major towns. (TIF) [file pbio.3000247.s007.tif]

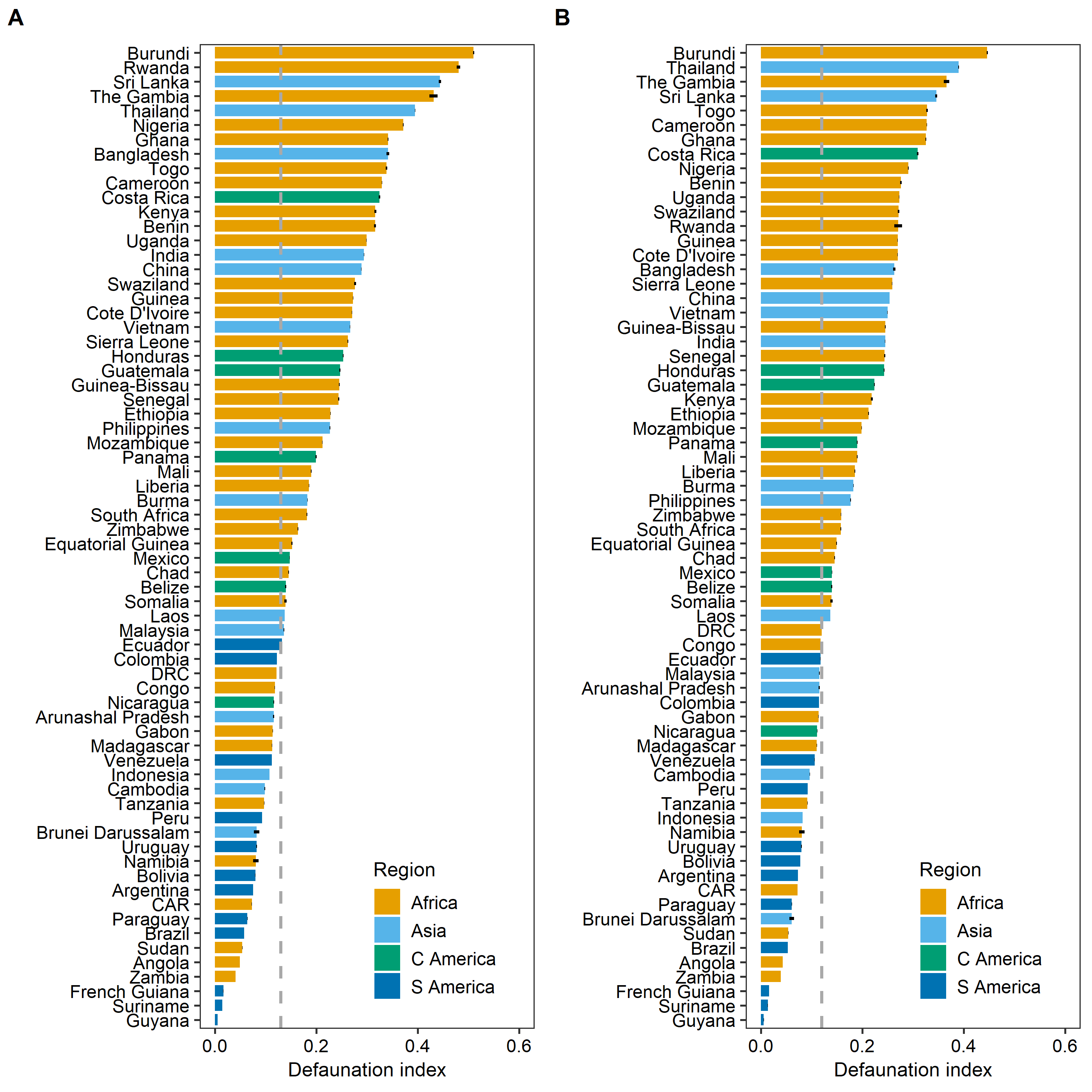

Supplement: S7 Fig — Colors denote different regions. Available at https://figshare.com/projects/Intact_but_emtpy_forests_Patterns_of_hunting-induced_mammal_defaunation_in_the_tropics/31118. CAR, Central African Republic; DI, defaunation index; DRC, Democratic Republic of Congo. (TIF) [file pbio.3000247.s008.tif]

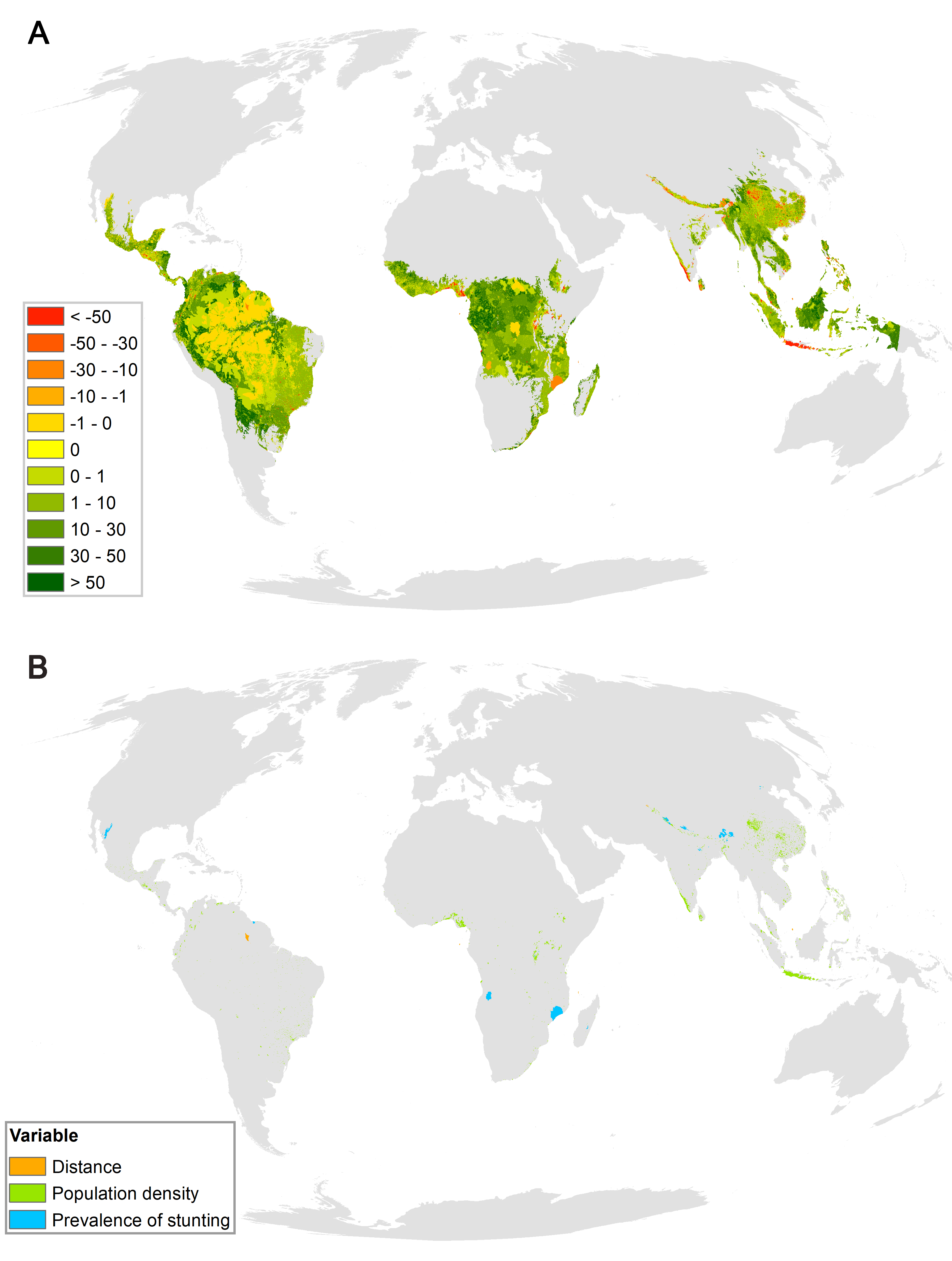

Supplement: S8 Fig — (A) Geographic areas inside and outside the socioeconomic domain covered by our data, as estimated by the MESS. The values represent the similarity between each grid cell in pantropical range and those in the reference data set used to fit the models. Values range from positive (green) to negative (red). Positive values represent interpolation areas with similar socioeconomic factors (distance to hunters’ access points, HPD, and prevalence of stunting) than those used to fit the models that are covered by our data set. Negative values indicate localities where at least one socioeconomic variable is outside the range of socioeconomic variables in our data set. (B) Main variable that is dissimilar in each grid cell compared with the socioeconomic domain in our data set. Orange, distance to the nearest rural settlement; green, HPD; blue, prevalence of stunting. Available at https://figshare.com/projects/Intact_but_emtpy_forests_Patterns_of_hunting-induced_mammal_defaunation_in_the_tropics/31118. HPD, human population density; MESS, multivariate environmental similarity surface. (TIF) [file pbio.3000247.s009.tif]

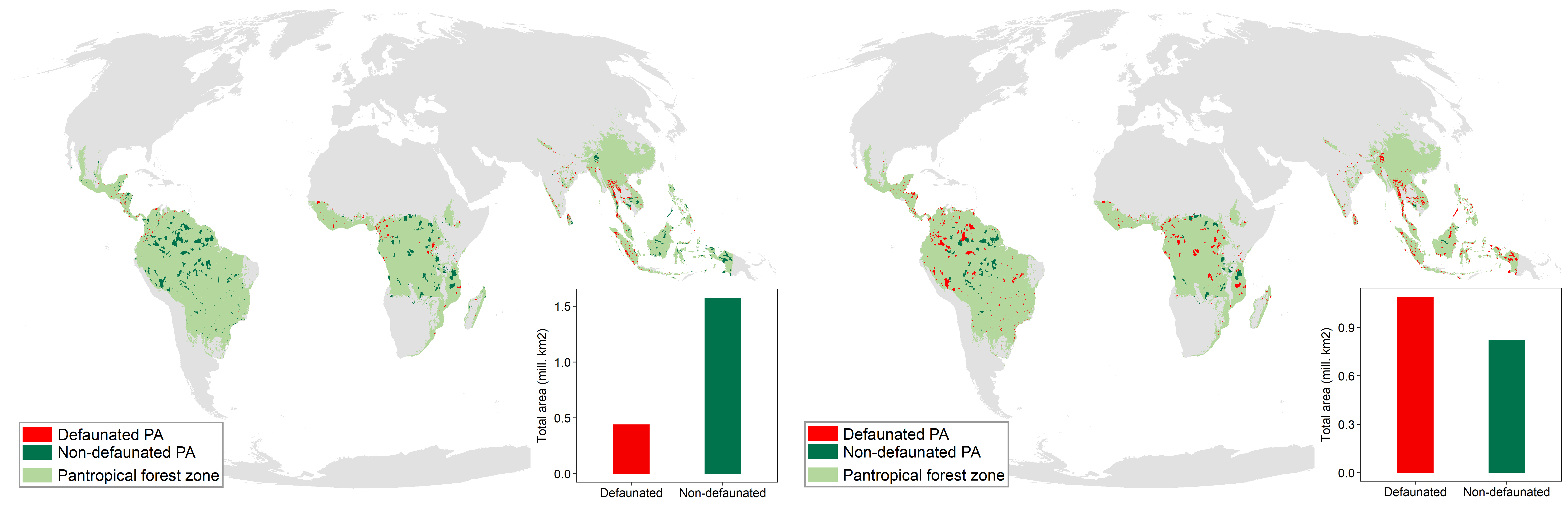

Supplement: S9 Fig — Available at https://figshare.com/projects/Intact_but_emtpy_forests_Patterns_of_hunting-induced_mammal_defaunation_in_the_tropics/31118. IUCN, International Union for Conservation of Nature; PA, protected area. (TIF) [file pbio.3000247.s010.tif]

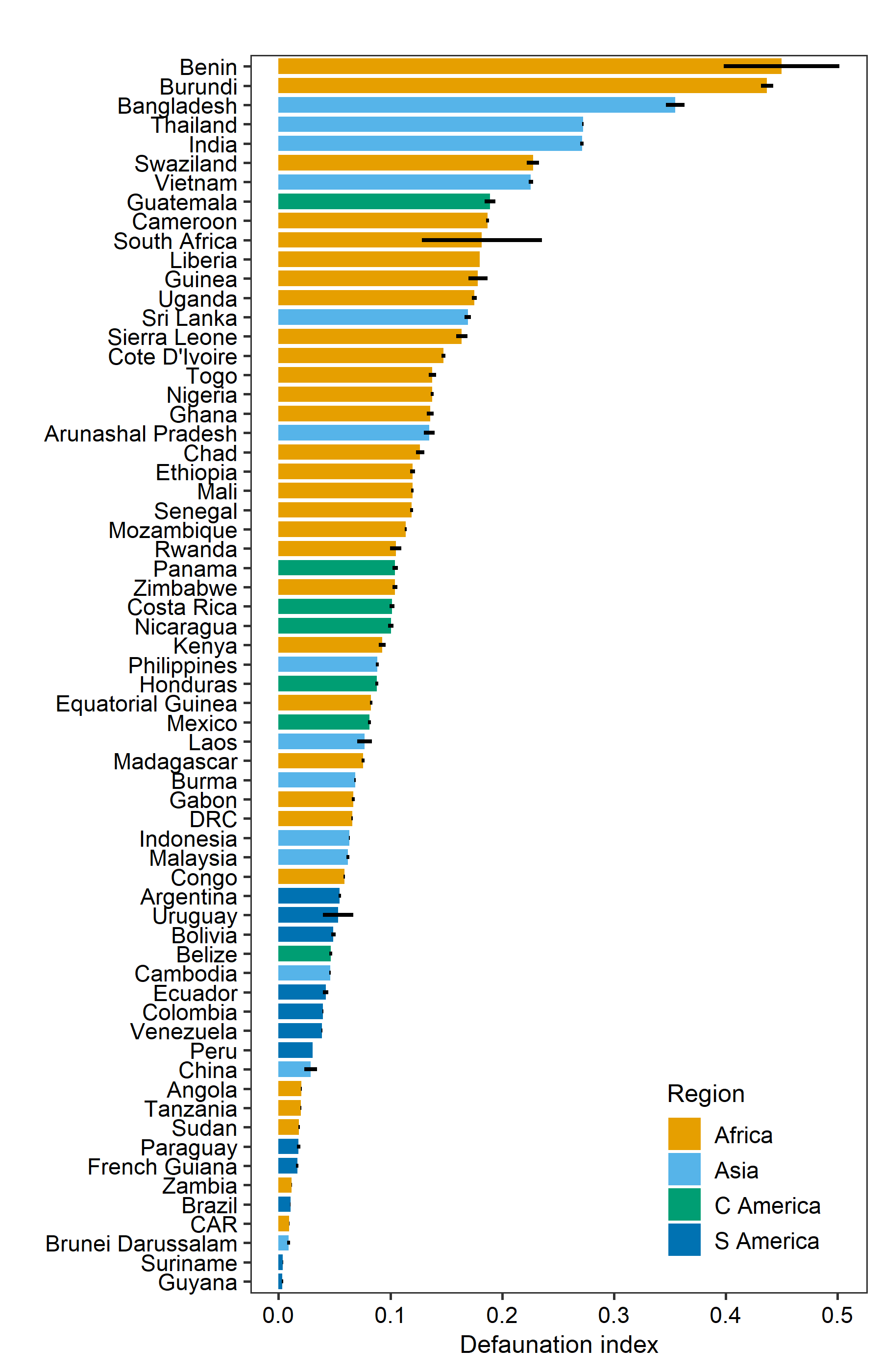

Supplement: S10 Fig — Colors denote different regions. Available at https://figshare.com/projects/Intact_but_emtpy_forests_Patterns_of_hunting-induced_mammal_defaunation_in_the_tropics/31118. CAR, Central African Republic; DI, defaunation index; DRC, Democratic Republic of Congo; IUCN, International Union for Conservation of Nature; PA, protected area. (TIF) [file pbio.3000247.s011.tif]

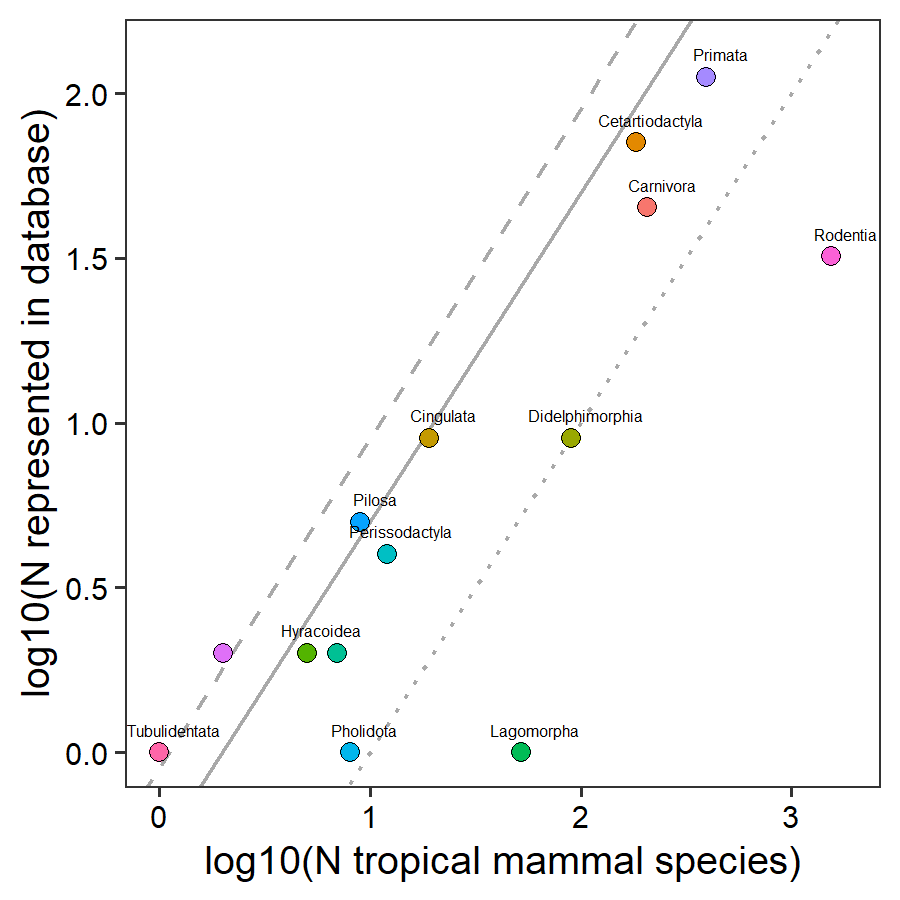

Supplement: S11 Fig — Lines show 10% (dotted), 50% (solid), and 90% (dashed) representations of the predicted species in our data set. Available at https://figshare.com/projects/Intact_but_emtpy_forests_Patterns_of_hunting-induced_mammal_defaunation_in_the_tropics/31118. (TIF) [file pbio.3000247.s012.tif]
