# Supplementary material for: Intact but empty forests? Patterns of hunting-induced mammal defaunation in the tropics
Source: PLoS Biol. 2019 May 14;17(5):e3000247. doi: 10.1371/journal.pbio.3000247 (PMC6516652; doi:10.1371/journal.pbio.3000247)
Supplement: S1 Table — BC, book chapter; DT, doctoral thesis; MT, master thesis; SP, scientific publication; TR, technical report. (DOCX) [file pbio.3000247.s013.docx]

**Table S1**. List of data sources included in our analyses and the associated metadata: author and year, type of source (SP: Scientific publication, MT: master thesis, DT: Doctoral thesis, TR: Technical report, BC: Book chapter), location, habitat, order, type of access point, type of hunting, legality status, number of studies and methods used in each source.

| **Data source** | **Source type** | **Location** | **Habitat** | **Order** | **Access point** | **Type of hunting** | **Legality** | **Nº studies** | **Methods** |
| --- | --- | --- | --- | --- | --- | --- | --- | --- | --- |
| Aliaga-Rossel, 2011 | DT  [1] | Asunción del Quiquibey forest and Madidi NP (Ersama), Bolivia | Piedmont submontane evergreen forests and moist lowland forests | Cetartiodactyla, Cingulata, Carnivora, Rodentia, Didelphimorphia, Pilosa, Perissodactyla (17 spp) | settlement | subsistence | legal | 1 | Species abundance (n plot with tracks/total track days) was estimated in hunted (10 km from village), moderately hunted (10-15 km) and unhunted forests (> 20 km) using track traps. In each forest 6 transects were randomly located 2 km apart and 9 track traps were set every 60 m. Each track was reviewed every 4 days and then the presence of mammals was recorded, identifying mammals based on field guides and with the assistance of indigenous hunters. Control distance: 20 km |
| Altrichter, 2005 | SP  [2] | Impenetrable, W Chaco province, N Argentina | semi-arid forest | Cetartiodactyla (3 spp) | settlement | subsistence | legal | 1 | Species densities (n/km2) were estimated using line transects in 3 different sites, 2 hunted sites of 18 and 16 km2 and a national park site of 12 km2. In each site a total of approximately 400km was walked which includes 2 extra repeats. Transects had various lengths and crossed the entire area. Local hunters never find the species within 1.5km from a settlement, and they never go any further than 5km. Control distance: >5km. |
| Aquino et al., 2014 | SP  [3] | Northeastern Peruvian Amazonia, Peru | tropical forest | Cetartiodactyla and Perissodactyla (5 spp) | settlement | subsistence & comercial | not specified | 1 | Species densities (n/km2) were estimated using line transects in low (Curacay river, N = 8 transects, length: 3-5 km) and high hunting pressure forests (Tigre-Nanay rivers, N = 12 transects, length: 3-5 km) in the northeastern Peruvian Amazonia. Surveys were conducted in Nov-Dec 2012 in Curacay (total transect length: 610 km), and in Feb-Jun and Aug-Oct 2013 in Tigre-Nanay river area (total transect length: 1218 km). Control distance not clear. |
| Atickem et al., 2011 | SP  [4] | Bale mountains, CS Ethiopia | montane grassland and woodland | Cetartiodactyla (1 sp) | settlement | commercial | illegal | 2 | Number of pellets (pellets/plot) counted 1515 times at 10 different sites at varying distances from settlements. Parallel line transects were used to cover the entire area of the different sites. Patrol-controlled sites have higher mountain nyala abundance and are on average closer to settlements (range: 4500- 7500 m) that non-controlled sites (9600-34700), masking the effects of distance to human settlements. Species relative abundance is calculated separately for each of these two datasets. Other potential confounding effects are elevation, forest cover and slope. Control distance: 34.7km. |
| Averbeck et al., 2012 | SP  [5] | Lake Mburo National Park (LMNP) and  Ankole Ranching Scheme (ARS), SW Uganda | savanna | Cetartiodactyla (8 spp) | settlement | subsistence & comercial | illegal | 1 | Species abundance (group size/area) was estimated using road counts between July 1997-December 1999 in order to cover a large area. Four tracks were established, each with a total track length of 150 km. Two road transects were situated inside LMNP (control), and two in ARS (hunted). Distance to nearest settlement calculated with GIS. Control distance: 9 km |
| Barlow and Peres,2006 | SP  [6] | Tapajós-  Arapiuns Extractive Reserve (W Pará), Brazil | lowland ombrophilous forest (terra firme forests, primarily) | Primata, Cetartiodactyla, Cingulata, Rodentia, Carnivora, Pilosa, Perissodactyla (18 spp) | settlement | subsistence | legal | 1 | Species abundance (n/100km) was estimate using line transects. Animal surveys took place along four 4 km transects in the lower Maró, in a hunted area, located independently of existing trails which may have been used by hunters. Additionally, one 4 km and two 5 km transects were cut in unhunted forests in a headwater tributary of the Rio Maró, 12 km upstream beyond the last extractive settlement.  Control distance: 12 km. |
| Barnes et al., 1991 | SP  [7] | NE Gabon | tropical rainforest | Proboscidea (1 spp) | road & settlement | commercial | illegal | 2 | The number of droppings were counted using 15 transects perpendicular to 3 different roads (5 transects per road with at least 5km distance between the transects). For each dropping encountered, its distance to the starting point was recorded. Settlements are distributed in a narrow band along the road. Control distance: 19-20km. |
| Barrera-Zambrano et al., 2008 | SP  [8] | Amacayacu National Park, S Colombia | Campina and Campirana Forest | Primates (7 spp) | settlement | subsistence | legal | 1 | Population densities of primates and other diurnal mammal were estimated using line transects. Transects varied in length, with an average of 3.95 ± 1.31 km. Total sample length was 190.3 km in less hunted places (far from settlements, ca. 8 km) and 90.8 km in the site near communities. Transects were walked 9 and 8 days on average, in the sites far and near communities, respectively. Sampling took place between June – September 2003, June –November 2005 and February- March 2006. Control distance: 8 km. |
| Baur, 1998 | TR  [9] | Maya Biosphere Reserve, Aldea Carmelita, Guatemala | subtropical moist forest (1% agriculture + pastures) | Cetartiodactyla, Carnivores, Perissodactyla (7 spp) | settlement | subsistence and commercial | legal | 1 | Encounter rates (n/km) were estimated using 10 line transects (2.6 km long) located at different distances from the village. Direct and indirect observations were recorded. Sampling was repeated 27 times per month, during 14 months for a total of 966.5 km. Encounter rates are reported at 4-8, 8-12, 12-16 and 16-20 km from village. Largest distance assumed control, although the impact could be larger. Control distance: 18 km |
| Blake et al., 2007 | SP  [10] | Congo Basin: Gabon, Cameroon, Congo, Central African Republic and D.R. Congo | tropical rainforest | Proboscidea (1 spp) | road | commercial | illegal | 4 | Elephant density (n/km2) was estimated from dung piles counts in 4 different sites using systematic line-transect distance surveys and reconnaissance walks (total length: > 4000 km between 2003-2005, N transects: 47-147). DISTANCE was used for estimating population densities. Human hunting signs were also recorded. The closest distance to road was recorded at every dung pile. Hunting signs declined at increasing distances from roads overall. Control distance: 42-62 km (depending on site). |
| Blake et al., 2013 | SP  [11] | Yasuni National Park, Ecuador | tropical rainforest | Primata, Rodentia, Cingulata, Cetartiodactyla, Pilosa, Perissodactyla (15 spp) | road | subsistence & commercial | legal | 1 | Abundance estimated as n photos/100 trap days using camera traps located at mineral  Licks in one hunted site (YRS, 10-550 m to road) and in one non-hunted site (TBS 12-25 km away from any access point). Cameras were active from mid-January to mid-March 2012. Total number of trap-days ranged from 54 to 55 at YRS and from 35 to 66 at TBS. Control distance: 12-25km. |
| Blom et al., 2004 | SP [12] | Dzanga-Ndoki National Park and Dzanga-Sangha Reserve, SW C.A.R. | tropical rainforest | Proboscidea & Primata (2 spp) | road & settlements | commercial | illegal | 1 | The direct and indirect signs of species were counted using 8 20km long transects parallel to the same road (and settlements) at different (random) distances from it. Every transect was walked every month from January 1997 to August 1999 (with the exception of 2 transects which were added between March and April 1998 & 2 transects which were skipped during March 1999). Every transect was divided in 500m blocks and the number of blocks with direct and indirect signs of a species was divided by the total amount of blocks to obtain an estimate of abundance. Control distance: 23km. |
| Blom et al., 2005 | SP  [13] | Dzanga-Ndoki National Park and Dzanga-Sangha Reserve, SW C.A.R. | tropical rainforest | Primata, Proboscidea & Cetartiodactyla (10 spp) | road & settlements | subsistence | legal & illegal | 1 | Mean encounter rates per km (n/km) were calculated by walking 7 line transects of 20km long parallel to the closest road in 3 different study sites: 4 in a reserve, 1 on the reserve boundary and 2 outside the reserve (closest to the road). From April to June 1998 every transect was walked once. Every transect was divided in blocks of 500m and all the blocks with direct or indirect signs was divided with the total amount of blocks in that study site, and this has eventually been related to the mean distance of that study site to the nearest road. Control distance: 17.85km. |
| Bodmer et al., 1997 | SP  [14] | Tahuayo and Yavari Miri river basins, NE Peru | upland terra  firme tropical forests | Primata, Cetartiodactyla, Rodentia, Perissodactyla (16 spp) | settlement | subsistence | legal | 1 | Species abundance (groups/km) was estimated in nine trail systems in a 200  km2 section of a hunted area (Tahuayo) and in seven trail systems in two sections of the infrequently hunted area (Yavari Miri), which were 180 km2 and the other 200 km2. Trail systems usually had 3-5 transects each that ranged from 1 to 7 km in length, and each trail was surveyed  several times. Total sampling effort was 393-626 km in the Tahuayo site and 482-495 km in the Yavari Miri sites. Control distance: 40 km |
| Bowkett et al., 2008 | SP  [15] | Mwanihana and Matundu forests, Udzungwa Mountains National Park, E Tanzania | sub-montane and lowland semideciduous forest | Cetartiodactyla (3 spp) | road & settlement | subsistence & commercial | illegal | 1 | Species abundance (camera-trap rate) was calculated as the number of photographs of a species divided by the number of trap-days per site. Camera-trap sites were located at 0.5-km intervals along four 4 km transect routes established for primate and forest antelope surveys. Camera-trap rates were estimated for 60-80 days in 2 different area's in 2 different forests with 8 camera locations at each site. The distance to the nearest village for each camera-trap was calculated using a Garmin e-trex GPS unit. Control distance: 9-10km. |
| Briceño-Méndez et al. 2016 | SP  [16] | Calakmul Biosphere Reserve (CBR), Yucatán península, México | tropical rainforest | Cetartiodactyla  (2 spp) | settlement | subsistence | illegal | 1 | Species abundance (photos/camera days) was estimated using camera-traps and direct observations. Surverys were conducted between February 2014-February 2015 in two sites differing in their degree of protection and hunting pressure: the first was Calakmul Biosphere Reserve (a site with no hunting) and the second was the community of Nuevo Becal (a hunting site). Control distance: 20 km |
| Brodie et al. 2009 | SP  [17] | N Thailand | tropical seasonal mixed-evergreen forest | Cetartiodactyla, Primata (3 spp) | settlement | subsistence & commercial | illegal | 1 | Species abundance (n scat pellet or max groups heard) ) was estimated using line transects and auditory sampling. Four national parks with different hunting pressure were surveyed. None of the  study sites were affected by forest conversion. Poaching activities were assessed with interviews. Control distance: 17 km |
| Cabassu, 2010 | MT  [18] | Rio Platano Biosphere Reserve, Honduras | tropical rainforest | Carnivora, Cetartiodactyla, Cingulata, Pilosa, Perissodactyla, Primata, Rodentia(18 spp) | settlement | subsistence | legal | 1 | Direct and indirect signs of 21 species (n/km) were recorded in four six-km long transects around the village (indigenous hunting is concentrated in a six-km radius around settlements) and four 3-km long transects in Río Plátano biosphere reserve (unhunted), 15 km away from settlement. Each of the six km long transects in the hunted ted area were visited 6-8 times (survey effort: 180 km in 30 days). The 4 3-km long transects in the unhunted area were visited 10-12 times (survey effort: 132 km in 44 days). Control distance: 15 km. |
| Calderón-Quiñonez, 2010 | MT  [19] | Parque Nacional Soberanía (PNS), Península Gigante (PG) and Barro Colorado Island (BCI), Panama | lowland tropical moist forest | Primata, Cetartiodactyla, Rodentia, Carnivora, (9 spp) | settlement | subsistence & commercial | illegal | 1 | Species abundance (n/km) was estimated in three sites with different levels of hunting pressure (intense, moderate, control). 5-km line transects were surveyed in each site during 18 weeks, and animals and hunting signs were recorded. Control distance: 9 km |
| Caro et al. 1999 | SP  [20] | Katavi National Park, Tanzania | miombo woodland | Cetartiodactyla, Primata, Proboscidea, Carnivora  (23 spp) | road | subsistence | legal | 1 | Species densities (n/km2) were estimated using line transects in 4 sites with different hunting management regimes and pressure. A total of 2953 km of transects (20 in total) were driven across all areas. For each observed animal or group the perpendicular distance to the transect was recorded. Livestock and human activities were also annotated. Control distance: 13.6 km |
| Carrillo et al., 2000 | SP  [21] | Osa Peninsula, Costa Rica | tropical forest | Primata, Rodentia, Cingulata, Didelphimorphia, Carnivora, Cetartiodactyla, Pilosa, Perissodactyla (20 spp) | settlement & roads | subsistence & commercial | legal (illegal in CNP) | 1 | Species abundance (tracks/km or groups/km) was estimated for two protected areas, one nonhunted (CNP) and one hunted area (GDFR) during September and December 1990. Mammal tracks were recorded along pre-established trails. For primates, monkey troops were located along or near the trail and counted the group as one sighting. Total sampling effort was 65 km (29 km in CNP, N = & trails, and 36 km in GDFR, N = 8 trails). Distances from the midpoint of each trail to the nearest village or road were calculated with GIS and averaged per area. Control distance: 13.5 km. |
| Chiarello 1999 | SP  [22] | Linhares Forest Reserve (LFR) and Sooretama Biological Reserve (SBR), Espirito Santo Estate, Brazil | Atlantic tropical rainforest | Didelphimorphia, Cingulata, Primates, Carnivora, Perissodactyla, Rodentia, Lagomorpha (14 spp) | settlement & roads | subsistence & commercial | illegal | 1 | Mammal abundances (n/10 km) were estimated in two large reserves (ca. 20000 ha), one with low hunting pressure (LFR) and one with high hunting pressure (SBR), using diurnal and nocturnal line-transect sampling during Oct 1994- Apr 1996. All mammals > 1 kg were censused. The extreme north of LFR is contiguous with the other large reserve (SBR) but the latter is crossed in its eastern part by a highway. Straight trails of 1.5 – 2 km of length and 1.5 m wide were cut in each study site. Number of transects and survey effort were 30 and 80.1 km and 32 and 83.6 km in LFR and SBR, respectively. Distance to nearest settlement or road calculated with GIS. Control distance: NA. |
| Croes et al. 2011 | SP  [23] | Bénoué Complex (Bénoué NP, Boubandjida NP and Faro NP), N Cameroon | wooded savanna | Carnivora (2 spp) | settlement | commercial | legal | 1 | Carnivore abundance (tracks/km) was estimated using repeat sampling on 25 km stretches of road transects. Transects were surveyed in each national park (Bénoué NP, Boubandjida NP and Faro NP) and a corresponding similar area in hunting zones adjacent to each national park. Transects were repeat sampled 16 times. Distance to nearest village was determined for 1-km segments using GIS Arcview. Control distance: 16 km. |
| Cronin 2013 | DT  [24] | Gran Caldera-Southern Highlands Scientific reserve, Bioko Island, Equatorial Guinea | tropical rainforest | Primates (2,5 and 6 spp, depending on the site) | settlement | subsistence & commercial | illegal | 3 (three sites) | Reconnaissance (recce) walk methods were used to collect data on primate abundance, hunting pressure, and habitat structure. 26 recce walk routes distributed across three sites in the GCSH were surveyed in Jan-Jun 2011 and Jan- Feb 2012. Total survey effort: 416.40 km. The three sites: Belebu, Ureca, and Moraka Playa (Moraka), were chosen due to increasing remoteness and decreasing development, respectively. Recce walks originated from points at distance intervals of 100 m, 2 km, 4 km, and 7 - 10 km from southern beaches at Moraka, and the outer village perimeters at Ureca and Belebu. Control distance: 7-10 km. |
| Cruz et al. 2014 | SP  [25] | Green Corridor of Misiones, Argentina | Atlantic (sub-) tropical forest | Perissodactyla  (1 sp) | road | subsistence & commercial | illegal | 1 | Species abundance (mean prob habitat use) was estimated using camera traps and related to the distance to the nearest hunter access point in three study sites with different hunting pressure. Signs of poaching activities (poaching campsites, spent cartridges, artifi-cial saltlicks, etc.) were recorded in each site and informal interviews were conducted with park rangers, biologists, and inhabitants of rural areas near our study sites the principal access points used by poachers to enter the study areas (roads, forest borders, rivers, etc.). Antipoaching activities were also recorded. Total effort was 5205 camera-trap days in 117 stations. Control distance: 11 km. |
| Cullen Jr. et al., 2000, 2001 | SP  [26, 27] | Mata de Planalto, São Paulo, S Brazil | Atlantic tropical deciduous forest | Perissodactyla, Cetartiodactyla, Cingulata, Rodentia, Carnivora & Primates (11 spp) | settlement | subsistence | illegal | 4 (one per hunted site) | Species abundance (n/10 km and n/km2) was estimated with line transect surveys. Four to eight 0.5-0.8 km transects were placed in each of the 5 sites (fragments of remnant forest) each categorized as slightly and heavily hunted according to colonist settled within 5 km of the sites. Each transect was surveyed multiple times between May-Dec 1996 (Total: 2287km, 161 to 618km per fragment, mean: 381km). The protected area Morro do Diabo was used as control area. DISTANCE used for estimating densities. Control distance: >5 km. |
| Danquah, 2016 | SP  [28] | Bia-Goaso Forest Block (BGFB), Westerrn Ghana | tropical moist forest | Proboscidea (1 sp) | settlement | commercial | illegal | 1 | Elephant abundance (dung piles/km) was recorded using line transects in the wet season. 130 1-km transects were located in 3 strata based on the respective dung densities recorded in a previous recce survey. The stages of dung decay were classified based on the MIKE S System Poaching signs and logging activity were recorded in each transect, along with other environmental variables. Control distance: 18.5 km |
| Davies et al., 2008 | BC  [29] | Gola Forest, Sierra Leone | tropical rainforest and farmbush | Primates (7 spp) | settlement | subsistence & commercial | Illegal in Tiwai sites (control). Legal in the rest. | 2 (forest sites and farmbush sites) | Species densities (n/km2) were estimated in forest and farmbush sites with varying hunting pressure. In the two forest sites (Mogbai and Koyema), a standardized 1 km x 500 m survey grid with parallel transects 100 m apart was used to census primates. The perimeter of the grid was walked slowly (ca. 0.5 km/h) for five consecutive days, in alternating directions each day, in four or five different months and all primate group sightings and calls were mapped. The density of individuals was then determined by multiplying group densities by the average number of individuals per group. In the farmbush sites (Lalehun and Kondebotihun), a rectangular transect was sampled, with all sightings and calls being mapped. Survey effort varied between 50.0 - 71.7 km per site. On Tiwai, primate population densities were estimated during long-term studies using sweep samples in the forest (Whitesides et al., 1988), and using transect surveys in the farmbush (Fimbel, 1994). Distance calculated using GIS. Control distance: 1.5 km (farmbush), 3 km (forest) |
| De Andrade Melo et al., 2015 | SP  [30] | Virua National Park and Novo Paraiso Settlement, Roraima State, N Brazil | Campina and Campinarana Forest | Carnivores, Cetartiodactyla, Cingulata, Perissodactyla, Pilosa, Primates, Rodentia (24 spp) | settlement | subsistence | legal | 1 | Relative abundance (individuals/10 km walked) of medium- and large-sized mammals was estimated  using the line transect method in diurnal and nocturnal surveys. At Novo  Paraíso (hunted), three parallel trails 3 km apart were opened behind and perpendicular to three inhabited settlement plots. The trails were 5 km, 3.75 km and 4 km long. At Viruá (protected), three 5-km long trails were surveyed. Sampling effort was 420 km at each site. Control distance: 10 km. |
| Demmer et al., 2002 | SP  [31] | Tawahka Asangni Biosphere Reserve, E Honduras | tropical rainforest | Carnivora, Rodentia, Cetartiodactyla, Perissodactyla, Cingulata, Primata, (13 and 7 spp) | settlement | subsistence | legal & illegal | 2 (2 villages) | The number of animal encounters was recorded along 6 different hunting trails (3 from each village) with a total distance of 23km and 137 repetitions (31 times in 1995 and 38 times in 1996 in Yapuwas village and 23 times in 1995 and 45 times in 1996 in Krausirpe village). With each encounter they also recorded the distance from the start of the hunting trail. Control distance: >2.5km. Control distance may be too conservative. |
| Derby, 2008 | DT  [32] | Yasuni National Park, Ecuador | tropical forest | Primates (7 spp) | road & settlement | subsistence & commercial | legal | 1 | Primate density (n/km2) was estimated using line transect surveys. 4 km and 3 km transects were established in one hunted and one nonhunted site. The hunted site is located adjacent to the road (see also Suárez et al. 2013). Data were collected 2-3 days per month from Feb-Dec 2005. Control distance: 36 l. |
| Dethier, 1995 | TR  [33] | Dja Reserve, S Cameroon | tropical rainforest | Cetartiodactyla (7 spp) | settlement | commercial | legal | 1 | Species abundances (n/km) were estimated by walking 5 different 5km long line transects which lie 5km from each other at varying distances from the settlement (range: 2.5-22.5 km). Every transect was surveyed 6 times. Good experimental design. The last two transects are not hunted. Control distance: 17.5km. |
| Doherty, 2005 | DT  [34] | Chiquibul Forest Reserve, Columbia River Forest Reserve and Bladen Nature Reserve, Belize | tropical forest | Didelphimorphia, Rodentia, Carnivores, Cetartiodactyla, Pilosa (9 spp) | settlement | subsistence | Not specified | 1 | Obs/hour were recorded using infrared camera traps in three protected areas, one heavily hunted, other moderately hunted, and another non-hunted. Cameras were set at trees that were dropping or about to drop fruit, and that still had ten or more fruits on the tree. Cameras were left at tree sites until there were no longer fruits on the tree or ground, and no recorded animal activity Sampling effort was 10252 hours of observation, 5693.5 of which were daylight hours. Distances to nearest settlements were calculated in GIS. Control distance: 6.6 km. |
| Effiom et al. 2013 | SP  [35] | Afi Mt Wildlife Sanctuary, Mbe Mt Community Forest, Okwango division CRNP, Nigeria | tropical forest | Cetartiodactyla (duikers and red river hog pooled into 1 guild) | settlement | subsistence & commercial | Not specified | 1 | Mammal abundance (groups/km) was estimated using diurnal standardized line transect censuses during the rainy seasons in May and June 2009 and 2010 in three protected areas with paired sites each (one hunted and one protected, total = 6). Four transects, each of 1 km, were made in all sites. The two sites in a pair were chosen relatively close to be as edaphically and floristically similar as possible to each other. Distances to nearest settlements were calculated in GIS. Control distance: > 3.4 km |
| Effiom et al. 2014 | SP  [36] | Afi Mt Wildlife Sanctuary, Mbe Mt Community Forest, Okwango division CRNP, Nigeria | tropical forest | Primates, Rodentia, Hyracoidea, (5 spp and 1 pooled) | Settlement | Subsistence & commercial | Not specified | 1 | Same methods as Effiom et al., (2013) but abundance estimates (n/km) presented by species instead of by guild. Species included in this dataset were not included in Effiom et al. (2013). Distances to nearest settlements were calculated in GIS. Control distance: > 3.4 km |
| Emmons 1984 | SP  [37] | Madre de Dios, Peru | tropical forest | Primates, Cingulata, Rodentia, Carnivora, Pilosa, Cetartiodactyla, Didelphimorphia (31 spp) | road & Settlement | subsistence | legal | 1 | Encounter rates (n/10 km) were determined in several hunted sites and one hunted site. For this study only Tampobata (hunted) and Cocha Cashu (nonhunted) are compared since the other sites belong to other countries. Survey methods were line transects (diurnal and nocturnal) and trapping for small mammals (nocturnal). Nocturnal survey effort was 161.3 h and 116.4 km in Cocha Cashu (2987 trap nights for small mammals) and 21.6 h and 27.3 km in Tambopata (434 trap nights for small mammals). Diurnal survey effort was 65.3 h and 67 km in Cocha Cashu and 29.8 h and 52.3 km in Tambopata. Number of transects or repeats not clear. Distances to nearest settlement and road calculated with GIS. Control distance: 8.7 km |
| Endo et al., 2010 | SP  [38] | Manu National Park, Peru | tropical forest | Primates, Cetartiodactyla, Rodentia, Carnivores (16 spp) | settlement | subsistence | legal | 2 (two hunted sites) | Species densities (n/km2) and encounter rates (group sightings/10km/) were estimated using 20 5-km long line transects (ca. 90 km) at seven locations inside Manu Park. Each transect was surveyed for ca. 10 d within a 21-day period (mean ± SD = 13.6 ± 33.9km/site. The two hunted locations corresponded to two settlements, whereas the non-hunted locations were located at 15-70 km from the settlements. DISTANCE used to estimate densities. Control distance: > 43 km. |
| Espinosa-Andrade, 2012; Espinosa et al. 2018 | DT, SP  [39, 40] | Yasuni Biosphere Reserve, Ecuador | tropical forest | Carnivora, Cetartiodactyla, Cingulata, Didelphimorphia,  Perissodactyla, Pilosa, Rodentia (20 spp) | settlement | subsistence and commercial | legal | 1 | Camera traps (23-26) were placed in 4 sites with different accessibility (remoteness) and different hunting pressure. Kg/100 trap days of prey were converted into ind/100 trap days using body weights from Elton Traits. Jaguar densities were estimated using program CAPTURE. Distances to settlements were calculated after georeferencing camera traps and villages, and averaging the distance from each camera trap to the closest village. Control distance ~ 12 km. |
| Eves, 2006 | DT  [41] | Boundaries of Nouabale-Ndoki National Park, Congo | tropical forest | Cetartiodactyla (6 Cephalophus spp. pooled) | settlement | subsistence and/or commercial (depending on the site) | legal | 3 (three sites) | The relative abundance of ‘huntable’ duikers (Cephalophus spp) was estimated in the vicinity of villages (N=24) in three sites (Forest Management Units) using duiker calls along 25-km transects in 10 km sections each day over a five-day period (Jan-Apr 1996). Data is presented as Potential Rate of Return (PRR), which is an indicator of relative duiker abundance vulnerable to hunting calculated as the kg/hour observed along each 5-km segment of the transect. Regular hunting territories in the area extend to a distance of 15 to 30 km from villages. Kg/hour converted to ind/hour dividing by the pooled weight of the 6 species surveyed. Control distance: 25 km |
| Fay and Agnagna, 1991 | SP  [42] | N Congo | tropical rainforest | Proboscidea (1 sp.) | settlement | commercial | illegal | 1 | Elephant density was estimated by walking 401km of line transects in 4 different study sites (63 to 141.5km per site) from February to April 1989 and in June 1990. Every transect was divided in blocks of 500m and all the blocks with dung piles was divided with the total amount of blocks in that study site. This was eventually related to the mean distance of that study site to the nearest settlement. Control distance: 37.3km. |
| Fay, 1991 | SP  [43] | SW and SE Central African Republic | tropical rainforest | Proboscidea (1 spp) | settlement | commercial | illegal | 1 | The elephant density was estimated using dung piles counted at 14 line transects (mean=10.50km and SD=3.79km) distributed over 4 study areas. Each transect was split into 0.5km sectors and for each study area the amount of sectors with dung were divided by the total amount of sectors. This was compared with the average distance of each study site to the nearest settlement. Control distance: 25-34 km. |
| Fimbel et al., 2000 | BC  [44] | Lobeke Forest, SE Cameroon | tropical rainforest | Cetartiodactyla (5 spp, 4 aggregated in one group: red duikers) | road and settlements | subsistence and commercial | legal and illegal | 1 | Number of individuals/km2 estimated using line transects at different distance intervals from road and settlements (0-10, 10-20, 20-30, >30 km). 15 5-km transects were surveyed 3 times in each stratified zone. Density estimates were obtained from direct sightings and dungs using DISTANCE. Hunting pressure was reported as number of captures per distance interval. Control distance: 30 km |
| Fragoso, 1991 | BC  [45] | Belize | tropical rainforest | Perissodactyla (1 sp) | settlement | subsistence | illegal | 1 | Numbers of tapirs/10 km of tapirs were estimated for a hunted area 2 km away from closest settlement, and for an unhunted reserve 26 km away from nearest settlement. 10-km long canoe transects were performed along two rivers in each area during the day and night. Each 10-km transect was surveyed 114 times. Control distance: 26 km. |
| Glanz, 1991 | BC  [46] | Barro Colorado Island and Pipeline Road, Panama | tropical rainforest | Primates, Pilosa, Rodentia, Carnivora, Perissodactyla (14 spp) | road & settlement | subsistence & comercial | legal | 1 | Species abundance (sightings/km) was calculated using line transect sampling in several hunted and non-hunted areas. Here only Barro Colorado Island (BCI, control) and Pipeline road (PIP, hunted) areas are used since they have similar habitat characteristics (mature forests). In BCI, nine transects were surveyed at least 5 times (survey effort: 100 km) between 1980-1986. In 1977-1978, six transects were sampled (Total: 314 km). PIP was sampled in 1977-1978 on several transects: 1 2-3 km along Rio Limbo (3 repeats), an adjacent 1-km route (1 repeat) and 6 km in Rio Agua Salud y Frijoles Road areas (2 repeats). Distance to road calculated with GIS. Control distance: 9 km |
| Gray and Phan, 2011 | SP  [47] | E Phnom Prich Wildlife Sanctuary, Mondulkiri province, E Cambodia | tropical rainforest (deciduous and evergreen) | Cetartiodactyla, Proboscidea, Carnivora, Rodentia & Primata (12 spp) | settlement | subsistence & commercial | illegal | 1 | Between Dec.2008 and Aug.2009 intensive camera-trapping was conducted in east of Phnom Prich Wildlife Sanctuary, Mondulkiri province, Cambodia. Forty camera-trap locations were set up for >2700 camera-trap nights producing 707 independent encounters of 23 mammal species. Relative abundance index calculated as the number of independent photographs of each species per 100 camera trap nights (average ± SD: 67.9 ± 33 nights). For each the camera the distance to the nearest settlement was also recorded. Control distance: 11-15km. |
| Hall et al., 1997 | SP  [48] | Kahuzi-Biega National Park and Kasese region, E D.R. Congo | tropical rainforest | Proboscidea (1 spp) | road & settlement | commercial | illegal | 1 | Dung piles per km2 were estimated using line transects with a total length of 480km at 7 different locations (4 within the National Park and 3 just outside it) with 5 to 6 transects per site between January to August 1994 and March to June 1995. For each dung pile found, the distance to the nearest road was recorded. DISTANCE software was used to estimate densities. Human signs were also recorded (snares, trails, cuts, etc). Distance intervals are large and vary per site. Control distance: 20-35km. |
| Hall et al., 1998 | SP  [49] | Kahuzi-Biega National Park and Kasese region, E D.R. Congo | tropical rainforest | Primates (1 spp) | road & settlement | commercial | illegal | 1 | Ape nest densities (n/km) were estimated using variable strip width line transect sampling. Survey effort was 480km at 7 different locations (4 within the National Park and 3 just outside it) with 5 to 6 transects per site between January to August 1994 and March to June 1995. Human signs were also recorded (snares, trails, cuts, etc). Nest encounter rates were calculated for each sampling site, which were located at different distance intervals from roads and settlements. Distance intervals are large. Control distance: 20-35km. |
| Hart, 2000 | BC  [50] | Ituri Forest, R.D. Congo | tropical rainforest | Cetartiodactyla (7 sp but 5 aggregated in a single density estimate) | settlement | subsistence & commercial | legal | 1 | Duiker densities (n/km2) were estimated using net drives in hunted and unhunted areas. Survey teams consisted of 49 people. Drive locations for each census period were determined by random placement of the first drive. Drive efficiency was standardized per area. After each drive, all captured animals were recorded. Animal densities were calculated as the total number of animals detected divided by drive area. Control distance: 20 km |
| Hayward, 2009 | SP  [51] | Dwesa and Cwebe Nature Reserves, Eastern Cape, SE South Africa | subtropical forest | Cetartiodactyla, Perissodactyla, Rodents, Carnivores & Primates (11 spp) | road | subsistence & commercial | illegal | 2 (2 reserves) | The relative abundance index (RAI, n/100 m transect) of several mammal species was estimated using line transect sampling. Between March-July 2003 83 variable-width transects were walked in two reserves, 47 in Dwesa (mean: 484m ±69) and 37 in Cwebe (mean: 564m ±60). RAI was related to the distance to hunter access points (reserve boundary). Human signs (snares, cartridges) were also recorded. Variable sample size per distance interval (N= 3-15). Control distance: 3 km, probably conservative. |
| Hegerl et al., 2015 | SP  [52] | Udzungwa Mountains, Tanzania | lowland deciduous to submontane and montane  evergreen forests | Carnivores, Cetartiodactyla, Primates, Rodents, Hyracoidea, Macroscelidea, Proboscidea, (22 spp) | settlement | subsistence & commercial | illegal | 1 | Relative abundance index (species-specific number of events/ 100 camera days were calculated using camera traps in two forest sites (hunted: Uzungwa Scarp Forest Reserve, USFR and non-hunted: Mwanihana Forest, MF). In USFR two grids of 15 digital camera traps were set sequentially at a density of 1camera/km2. IN MF 60 camera traps were distributed at a density of camera/2km2. Sampling effort was 850 (mean per camera 28.3) and 917 (mean per camera 30.6) camera days in USFR (Dec 2013-Jan 2014 and Jan-Feb 2014) and Mwanihana Forest (Jul-Nov 2013), respectively. Species-specific detectabilities (p, probability of detection) and occupancies (ψ, proportion of sites occupied by a species), were modelled using single-species occupancy models. Distances to access points calculated with GIS. Control distance: > 8.2 km |
| Hema et al., 2011 | SP  [53] | Nazinga Game Ranch, Burkina Faso | savannah woodlands and shrublands | Proboscidea (1 sp) | settlements | commercial | illegal | 3 (3 seasons) | Elephant density (droppings/km) was estimated between 2006 and 2008 using a systematic transect design. A grid of sides 2 km was placed over the study area, and 54 1 km transects were laid at 4 -km intervals (random start). Hunting of other species is regulated in the ranch but illegal in case of elephants. Elephants seem to be attracted to villages and cases of crop-raiding were frequent, but poaching is particularly apparent in areas where law enforcement is lower (distant from guard posts). Control distance: 15-20 km. |
| Henschel et al., 2011; Henschel, 2008 | SP; DT  [54, 55] | Central Gabon | tropical rainforest | Carnivora, Proboscidea, Primates, Cetartiodactyla (11spp) | road & settlement | commercial | illegal | 1 | Using camera-trap data (Henschel, 2008), leopard population density and the relative abundance of prey and of human hunters were compared across four study sites at varying distances from settlements and from roads/railways, used as market access points. 87 camera stations were used: 15, 18, 23 and 31 per site, respectively, resulting in 4060 trap-days. Control distance: 19-29km. |
| Hill et al., 1997 | SP  [56] | Mbaracayu Reserve, E Paraguay | tropical rainforest | Primata, Perissodactyla, Cetartiodactyla, Rodentia, Carnivora & Cingulata (4 spp for Ache and 8 spp for Non-Ache hunters) | settlements (around reserve boundary) | subsistence | legal (Ache hunters) and illegal (Paraguayan and Brazilian peasants) | 2 | Encounter rate for different game species was estimated using over 1400 km of stratified random line transects. The transects (N = 91) generally started at a dirt road that traverse the reserve. All animal signs (direct and indirect) were recorded per 200 m of transect to derive an encounter rate. Hunting signs were also recorded to estimate extent of hunting pressure. Both indexes were related to the distance to the nearest access point for Ache or non-Ache Hunters. Control distance: >18km for Ache hunting, >11 km for Non-Ache hunting. |
| Hurtado-Gonzales and Bodmer, 2004 | SP  [57] | Tamshiyacu-Tahuayo Communal Reserve, Loreto, NE Peru | tropical rainforest | Cetartiodactyla (2 spp) | settlement | subsistence | legal | 1 | Species densities were estimated by walking line transects in 3 different study sites from 1991 to 1999 with different levels of hunting pressure. Densities calculated for Tahuayo/Blanco (heavily hunted, 0-14 km from village), the Lower Yavari-Miri (slightly hunted, 14-20 km), and the Upper Yavari-Miri (non-hunted) sites, which had 923, 889, and 941 km of line, transects censused; respectively, for a total of 2753 km of census. Distance intervals calculated from catchment area, some assumptions about distances made. Censuses were carried out from 1991 to 1999. Control distance: >20km. |
| Kano and Asato, 1994 | SP  [58] | Motaba River area, NE Congo | tropical rainforest | Primata (2 spp) | settlement | subsistence | illegal | 1 | The number of ape nests was counted using 12 line transects of various lengths starting from 4 different settlements. Total transect length was 222km. All the transects were repeated once. The location of a nest was recorded using GPS and related to the nearest settlement. All direct sightings and signs of apes, including vocalizations, nests, feeding remnants, dung, and footprints, and all signs of human activities such as hunting camps and honey-collecting sites, were recorded with a GPS. Densities in six equally spaced (5 km) distance intervals. Data was pooled for the 4 villages. Control distance: >25km. |
| Koerner et al. 2016 | SP  [59] | Makokou (Ogooué-Ivindo province), NE northeastern Gabon | tropical forest | Cetartiodactyla, Primates, Proboscidea Rodentia (28 spp) | settlement | subsistence | Not specified | 1 | Encounter rates (n/km) and densities (n/km2) were calculated from direct and indirect observations of large vertebrates. Monthly surveys were conducted from Dec 2013-Dec 2014 line using 24 2.5-km straight-line transects located in a gradient of hunting intensity (distance to villages). Hunting signs were recorded. Densities estimated using DISTANCE software. Control distance: >15 km |
| Kosydar et al, 2014 | SP  [60] | Chiquitano Forest, S Bolivia | tropical dry forest | Didelphimorpha, Carnivora, Cingulata, Rodentia, Cetartiodactyla, Pilosa, Lagomorpha, Perissodactyla (23 spp) | road | subsistence & commercial | legal & illegal | 1 | Species relative abundance (n traps/100 trap nights and n photos/100 camera nights) was estimated between 2007-2008 in a hunted area (< 1.5 km from road, N = 6 sites, total trap-nights = 1496), in fragmented-plus-hunted area (7 km from road, N = 5 sites, total trap-nights = 1344) and in a control area (24 km from road, N = 5 sites, total trap-nights = 1279). Control distance: 24 km. |
| Kuehl et al., 2009 | SP  [61] | Moukalaba Doudou National Park, SW Gabon | (secondary) tropical rainforest | Primata (2 spp) | road & settlement | subsistence & commercial | illegal | 1 | From April 2004 to July 2005, ape sleeping nest (densities were sampled using point transects (100 m diameter). Each point transect was sampled with decreasing intensity from the center towards the edge. Five observers walked in concentric circles recording ape nests with an integrated GPS and data logging. Hunting intensity is measured as both the Euclidean distance to villages and human population centers (Gamba, Tchibanga, and Mandji) and a cost–weight distance that accounted for variation in travel speed. A total of 540 point transects were walked at 18 (not independent) sites. Control distance: 35.5 km. |
| Kümpel et al., 2008 | SP  [62] | Monte Mitra area, Monte Alén National Park, W Equatorial Guinea | tropical rainforest (mature secondary evergreen humid closed forest) | Primata (4 spp) | settlement | commercial | illegal | 1 | Species densities (n/km2) were estimated using 8 line transects randomly placed at each of the 2 study sites, one close to the settlement and frequently hunted and the other far from the nearest settlement not (or lightly) hunted). Minimum distance between the transects was 300m. The transects far away (control) were walked between February to June 2003 (208km total) and the transects close to the settlement between July 2003 and January 2004 (200km total). Hunter movements were tracked by GPS. Densities estimated using DISTANCE software. Control distance: 35km. |
| Kun-Rodrigues et al., 2014 | SP  [63] | Ankarafantsika National Park, N Madagascar | Dry deciduous forests and savannas | Primata (1 sp) | road | subsistence | illegal | 1 | Species density (n/km2) was estimated at different sites located at different distances from a national road that crosses through a National Park. At each site, three to six line transects were surveyed four to six times during 2–3 days (Aug-Sep 2009). Control distance: 36 km. |
| Lahm, 1993 | DT  [64] | NE Gabon | tropical rainforest | Proboscidea, Primates, Cetartiodactyla, Carnivores, Rodentia (18 datapoints per distance interval, Carnivores, 11 spp, Prosimians: 4 spp, represented by 1 datapoint each) | road & settlements | subsistence & commercial | legal and ilegal | 1 | Species density (ind/km2 or mean number/ 10 km) was estimated using line transect surveys between 1988 and 1991. 16 line transects were established: 6 transects were located near villages (0-5km), 6 transects at intermediate distances (7.5-9.5 km) and 4 transects in remote areas (30-50 km). Each transect was surveyed 6 times. Control distance: 30-50km. |
| Lahm et al., 1998 | SP  [65] | NE Gabon | tropical rainforest | Primata (1 spp) | road & settlements | subsistence | legal | 1 | Primate species density (ind/km2 ) was estimated using line transect surveys. 16 different 5-km long transects were surveyed between 1988 and 1991. The starting points of the transects were randomly selected: 6 of them were close to a road, 6 of them started 5-7km from the road & 4 of them started further away in the forest. From each transect 6 replicate counts were made. Monkey density was related to the mean distance of each transect to a road. Control distance: 50km. |
| Laurance et al., 2006 | SP  [66] | SW Gabon | tropical rainforest | Cetartiodactyla & Proboscidea (7 spp) | road | commercial | legal | 1 | Species abundances were estimated using 5 standardized transects (1 km long and parallel to the road at 5 different distances from it) per site. 12 study sites were selected using a stratified random design and foot surveys. All sites were at least 2.4km apart. In 2004 all the transects were surveyed 2 times in the dry and 2 times in the wet season, with at least 2 weeks’ time between the different surveys. Control distance: 1.2km. |
| Levi et al. 2011; Mitchell and Raez Luna, 1991 | SP, TR  [67, 68] | Manu National Park, Peru | tropical rainforest | Primata (2 spp) | settlement | subsistence | legal | 1 | Species encounter rates (n/km) were estimated using line transects. One 8-km transect was conducted radially outward from the Matsigenka native community of Yomybato (1991), and several 4.5 km transects were conducted later on (2006).  Control distance: 8 km. |
| Lin et al.,2008 | SP  [69] | S China | tropical bamboo and humid conifer forest | Proboscidea (1 sp) | road and settlements | commercial | illegal | 1 | Elephant frequency of signs proportion of plots with elephant presence) was recorded at different levels of human disturbance (< 0.5km, 0.5-2km and >2km from settlements and roads). Thirty transects 20 m wide and 10 to 50 km long were monitored. Plots (20 x 20 m) were sampled at intervals of 2 km along the transects. Authors recorded vegetation type, human disturbance, and presence of fresh elephant signs (footprints, dung piles, grubbing and foraging), among other variables. Poaching suggested as threat. Control distance: < 2 km. |
| Linder (2008), Linder et al. (2011) | DT, SP  [70, 71] | Korup National Park, SW Cameroon | tropical forest | Primates (7 spp) | settlements | subsistence & commercial | illegal | 1 | Standard line transect methods were used to collect data on primate abundance (encounter rates), habitat characteristics, and hunting pressure in 10 transects located in a gradient of hunting intensity. Transect length varied between 1.825 and 5.180 km. Transects were surveyed monthly between October 2004 through June 2005 (southern transects) and between December 2004 through April 2005 (northern transects). Total survey effort was 320.2 km. Distances from each transect to the nearest village were calculated using GIS. Distance was negatively related to hunting pressure (hunt signs/km). The transect with the lowest hunting pressure and located at largest distance is used as control. Control distance: 10.8 km. |
| Lwanga ,2006 | SP  [72] | Kibale National Park, Uganda | secondary tropical forest | Cetartiodactyla, (2 spp) | settlement | subsistence and commercial | illegal | 1 | Duiker abundance (n/km) was estimated using line transects between July 2002 and August 2004. Two monthly censuses were conducted along three transects, of which, two (colonizing forests 1 and 2) were located in colonizing forests naturally replacing anthropogenic grasslands and one in old growth forest. Only the two colonizing forests are used in this study to avoid confounding effects due to habitat differences. The two colonizing forests had different hunting pressure (high vs low). Control distance: ~8.6 km |
| Madhusudan and Karanth, 2000 | BC  [73] | Nagarahole National Park, S India | tropical moist and dry deciduous  forests | Cetartiodactyla, Primata, Proboscidea, Rodentia (9 spp) | settlement | subsistence and commercial | illegal | 1 | Species densities (n/km2) were estimated in two ecologically similar sites, Nalkeri and Arkeri in South India, but with different hunting pressure. Both sites were surveyed with distance sampling field methods. Four permanent transect lines were surveyed  in Arkeri (total length = 10.4 km) and five lines in Nalkeri (total length = 12.6 km). The total sampling effort was 271.4 km for Arkeri and 327.6 km for Nalkeri. Mean  animal densities were calculated with DISTANCE. Interviews were conducted to determine hunting patterns and intensities. Control distance: 5 km |
| Maldonado, 2010 | DT  [74] | Amacayacu National Park, Colombia | tropical rainforest | Primata, Cetartiodactyla, Rodentia, Cingulata, Carnivora, Perissodactyla (10 spp) | settlement | subsistence | legal | 1 | Species densities (n/km2) were estimated using line transects, which were conducted on a monthly basis over a period of 41 months from June 2005 to May 2009. Four sites with different hunting pressure and located at different distances from villages were surveyed ((Bacaba and Pucacuro in Mocagua, and Agua Blanca and Agua Pudre in San Martín). Hunting pressure was determined by quantifying the total biomass extracted by hunters over a 48 month period in each hunted site. Densities were estimated with DISTANCE. Total sampling effort: 2262 km and 236 days.  Control distance: 11.6 km |
| Marshall et al., 2005 | SP  [75] | Uzundwa Mountains (West Kilombero Scarp Forest Reserve and New Dabaga/Ulangambi ForestReserve | montane forest | Primates ( 3 spp) | settlement | subsistence | illegal | 1 | Monkey sighting frequencies per km transect (n/km) and group size were recorded in two census transect routes, each in a heavily hunted site and in an unhunted/slightly hunted site. Transects were 2.520–3.192 km long and sampled montane forest habitats. Routes were repeated (9-11 times). Distances were calculated using GIS and georeferenced transects (2.5, 3, 9, 14.5 km). Control distance: 14.5 km |
| Marshall et al., 2006 | SP  [76] | East Kalimantan, E Borneo | tropical rainforest | Primata (1 spp) | settlement | subsistence | illegal | 1 | Orangutan density per km2 was estimated by counting nests and recording its distance to the nearest settlement at 22 sites (minimum 5km apart) between December 2001 and August 2004. At each site 1-10 midlines of 1km in length and minimum 1km apart from each other were randomly placed with a total of 108 midlines. Perpendicular to these midlines 762 transects were randomly placed with a length 500m each and a minimum distance of 100m towards each other. One distance interval is larger than the rest to balance sample size per distance. Control distance: >60km. |
| Martins, 1993 | MT  [77] | Iaco river, Acre, Brazil | tropical rainforest | Primata, Cetartiodactyla, Rodentia (8 spp) | settlement | subsistence | legal | 1 | Species density (n/km2) was estimated using line transects in two sites ecologically similar but with different hunting pressure (Rio Iaco and Fazenda União, where hunting has ceased three years before). Hunting signs were recorded. Total sampling effort: 180 km.  Control distance: 16 km |
| Mena et al., 2000 | BC  [78] | Quehueiri-ono community, Napo Province, Ecuador | upland humid tropical forest | Cetartiodactyla, Primates, Rodentia, Carnivora (25 spp) | settlement | subsistence | legal | 1 | Species densities (n/km2) were estimated using line transect sampling. Four 2-km transects were located in infrequently hunted areas (16 km away from settlement) and other 4 2-km transects were located in persistently hunted areas (ca. 4 km radius around settlement). Each transect was surveyed for a 2-day period each month during 11 months (N = 60 repeats in each area, total length: 117.2 and 118.6 km in each area). Species density (n/km2) was estimated using detection distances and areas (detection distance x transect length). Control distance: 16 km. |
| Muchaal and Ngandjui, 1999 | SP  [79] | W Dja Reserve, S Cameroon | tropical rainforest | Cetartiodactyla, Primata, Carnivora, Rodentia & Pholidota (17 spp) | settlement | subsistence & commercial | legal and illlegal | 1 | Direct and indirect signs of animal species were collected by walking 4 different transects of 5x0.03km in 4 different study area's (with some differences in hunting pressure), each with a different distance to the nearest settlement. Between July 1994 and December 1995 a total of 450km were walked. Transect 1 (1.5km from settlement) was walked 31 times, transect 2 (15.0km) 19 times, transect 3 (30.0km) 27 times and transect 4 (45.0km) 13 times. Control distance: >40km. |
| Nielsen, 2006 | SP  [80] | Udzungwa Mountains, Tanzania | montane forest | Cetartiodactyla, Rodentia (3 spp) | settlement | subsistence | illegal | 1 | Line transect sampling was used to estimate relative densities. A total of 19.40 and 11.05 km were surveyed, divided on 5 and 4 transects in NDUFR (hunted, surrounded by 6 villages at 1.5-14 km from reserve boundary) and WKSFR (not or low hunting, difficult access), respectively. Indirect signs were counted and DISTANCE was used to estimate relative densities. The surveys were conducted in the dry season from July to late October 2001. Control distance: > 14 km. |
| Nijman, 2004 | SP  [81] | Kayan Mentarang National Park, East Kalimantan, E Borneo | tropical rainforest (primary, old secondary and young secondary forest) | Primata (1 spp) | settlement | subsistence & commercial | illegal | 1 | Groups of monkeys were counted using repeated line transect surveys between September and December 1996. Transects were situated in 4 different forest types: primary hill forest (four transects with a total length of 10.75 km repeated 44 times), primary riverine forest (one transect of 4.9 km repeated 6 times), old secondary forest (one transect of 3.3 km repeated 20 times) and young secondary forest (two transects totaling 4.6 km repeated 19 times). Control distance: 5km. |
| Noss, 2000a | BC [82] | Dzangha-Sangha Special Reserve, CAR | tropical forest | Cetartiodactyla (4 spp) | road and settlements | Subsistence | legal | 1 | Species densities (n/km2) estimated by surveying line transects (total: 97 km). The survey consisted of 4 parallel straight 2-km line transects and 2 1.5-km perpendicular transects connecting the ends of the 4 parallel transects. Areas sampled were located at 0-5, 5-10 and 10-15 km from the road and settlements. Number of hunts per sampled area used as proxy of hunting pressure. Control distance: 10-15km |
| Noss, 2000b | BC  [83] | El Chaco, Bolivia | xerophilous forest | Cetartiodactyla, Cingulata, Perissodactyla (10 spp) | settlements | Subsistence | legal | 1 | Species densities (n/km2) and encounter rates (n/km) were estimated in line transects located in two sites, one hunted (20 5-km transects, total survey effort: 6560 km, Aug 97-Aug 99) and one non-hunted (6 2-km transects, total survey effort: 1106 km, Jan-Oct 98). Distances were calculated with GIS. Control distance: 23 km. |
| Novack, 2003 | MT  [84] | Maya Biosphere Reserve, Guatemala | lowland tropical rainforest | Cetartiodactyla, Carnivora and Rodentia (6 spp, 2 spp aggregated in 1 group) | settlement | subsistence | legal | 1 | Species densities were estimated using line-transect sampling and DISTANCE software. 12 transects with similar habitat characteristics were sampled within the hunted area at 6-10 km from the village of Uaxactun (N=6, total: 1045 km) and in a protected unhunted area 32 km away (N=6, total= 1414 km) between Aug 2000-jul 2001). Control distance: >32 km |
| Nuñez-Iturri , 2007, Nuñez-Iturri and Howe, 2007; Nuñez-Iturri et al., 2008 | DT, SP, SP [85-87] | Madre de Dios State, SE Peru | lowland mature tropical forest | Rodentia, Cetartiodactyla, Primates and Carnivora (35 sp) | settlement | subsistence & commercial | legal | 1 | Species densities were estimated to quantify differences in mammal abundance between 3 hunted and 3 protected sites with similar vegetation composition. Diurnal and nocturnal censuses of mammals were performed using a standardized line- transect method. Total transect length was 700 km and 630 km in protected and hunted sites. The three hunted sites were located at 3-8 km from hunting villages, and protected sites were at least 30-40 km away. Hunting with firearms. Control distance: 30 km |
| Paciulli, 2004 | DT  [88] | Mentawai Islands, Indonesia | lowland evergreen tropical rainforest (unlogged, logged 10 ya and logged 20 ya) | Primates (4 spp) | settlement | subsistence | legal | 3 (three forest types) | Data on primate species’ densities were gathered using line transects in nine forest sites with different hunting pressure and located at different distances from villages. Forest sites were categorized as unlogged forests, forests logged 10 years ago and forests logged 20 years ago. In each of the nine survey areas, three 4 km long independent transects were surveyed. Each transect was walked three times (total line length per site: 36 km). Control distances: 2, 5 and 7 km depending on forest type. |
| Parry et al., 2009 | SP  [89] | Jarí region, NE Brazilian Amazon | Landscape matrix of upland primary terra firme forests, secondary forests, and industrial plantation forests | Primates, Rodentia, Cetartiodactyla (7 spp) | settlement | subsistence | legal | 1 | Mammal and bird densities were estimated with standardized line transect surveys at eight unhunted (98 surveys, total effort: 344 km) and six hunted primary forest sites (74 surveys, 225 km). Unhunted sites were in remote parts of the landscape with limited proximity or access to communities or urban areas. Hunting signs were recorded. Distances from each site to nearest village obtained with ArcGIS. Mean distance to nearest village ± SD for hunted sites: 5.0 ± 1.3 km; for unhunted sites: 16.2 ± 11.3. Control distance: >16 km |
| Paviolo et al. 2009, Paviolo, 2010 | TR, DT  [90, 91] | Corredor Verde de Misiones, Argentina | Atlantic (sub-) tropical forest | Cetartiodactyla, Perissodactyla (5 spp) | road | subsistence  & commercial | illegal | 1 | Three areas with different levels of hunting pressure (low, intermediate, high) were surveyed with camera traps. The area with low hunting pressure is well protected by rangers. Species abundance (n camera traps with records/total camera traps) was related to the distance to the nearest access point. Total effort was 12 843 trap-days in 216 stations. Control distance: 9 km. |
| Perera-Romero et al., 2015 | BC  [92] | Caura river, Venezuela | tropical rainforest | Didelphimorphia, Carnivores, Cetartiodactyla, Perissodactyla, Cingulata, Pilosa, Rodentia (21 spp) | settlement | subsistence | legal | 1 | Encounter rates (Events/1000 trap nights) were estimated using camera traps in three areas (two hunted and one nonhunted and not inhabited) in March – June 2011, 2013 and 2014. Number of trap stations was 58, 53 and 52 in the nonhunted area (Ka’kada), and the tow hunted areas (Yudiña-Ayawaña and Anadekeña-Juwutuña), respectively. Survey effort was 1933, 2220 and 1508 trap nights. Distances to nearest settlement calculated with GIS. Control distance: 24 km. |
| Peres and Lake, 2003 | SP  [93] | Amazon basin, Brazil | tropical rainforest | Primata, Cetartiodactyla, Perissodactyla & Rodentia (12 spp) | road & rivers | subsistence | legal | 1 | Species abundance (n groups/10 km) was estimated using a standardized series of line-transect from 1987 to 2000 at 21 heavily or moderate hunted sites. 45 transects which were walked 13 to 15 times were used, contributing to a total distance of 2753 km over 576 walks. For each sighting the GPS location was recorded and compared to the distance of the closest road (in sections of 500m). Data is pooled for all sites. Control distance: 4.5-5km. |
| Peres and Nascimento, 2006 | SP  [94] | A ‘Ukre village, SE Amazon, Brazil | tropical rainforest | Primata, Perissodactyla, Cetartiodactyla, Carnivora, Rodentia, Cingulata (18 spp) | settlement | subsistence | legal | 1 | Species densities (n/km2) were estimated using line-transect surveys (4-5 km long) in two sites with different hunting pressure. One study site had been unhunted for the last 5 years and is located at 12-18km from the settlement, whereas the other site lies 0-5.5 km from the settlement and is frequently used to hunt. Total sampling effort was 248.5 km in the unhunted site (1994-1997), and 199.4km in the hunted site (1997 – 1999). Control distance: 12-18km. |
| Peres, 1990 | SP  [95] | Several sites in Brazilian Amazon | tropical rainforest | Primates (15 spp) | settlement | subsistence | legal | 4 (4 hunted sites) | Species density (n/km2) was estimated using repeated line transects (4-5 km long) at 7 sites with different levels of hunting pressure in Brazilian Amazonia (period: 1987-1988). Transects were repeated 10 times and all encounters with primate groups or individuals were noted. Data collected from additional sources (4 sites) was not used in the analyses of this article. Hunting pressure extended up to 9 km from human settlements. Densities of primates in control sites (4, which were relatively close to each other) were averaged per species and used as control density. Control distance: > 9 km |
| Peres, 1996 | SP  [96] | CW and E Brazil | tropical rainforest | Cetartiodactyla (1 spp) | settlement & rivers | subsistence | legal | 9 (9 hunted sites) | Species density (n/km2) was estimated using repeated line transects (4-5.6 km long) at 23 sites with different levels of hunting pressure (period: 1987-1994). Total sampling effort was 44-359 km per site (mean=103km, SD=65). Hunting pressure extended up to 12 km from the settlement or nearest river according to interviews with hunters. This hunting distance interval was used in each hunted site. Densities of peccaries in control sites (4, which were relatively close to each other) ranged 8.8-11.7 (mean: 10; SD: 1.38). This value was used as control density (approximate carrying capacity). Control distance: >12km. |
| Pia, 2011; Pia et al., 2013 | DT, SP  [97, 98] | Quebrada del Condorito National Park, C Argentina | high tussock grasslands and shrublands, with granite outcrops | Carnivora (2 spp) | settlement | subsistence | illegal | 2 (outside and inside the National Park) | Number of signs (tracks, faeces, or direct sightings) of culpeo foxes and pumas were counted in 14 transects inside the National park (limited or no poaching), and 13 transects outside the park (higher hunting pressure) in 2007 and 2008. Transects (5 m x 1000 m) were set up on natural sandbanks along riversides, and were located at a wide range of distances to human settlements. Control distances: > 1600 m outside the NP, > 3000 m inside the NP. |
| Pianca, 2004 | MT  [99] | Piraniapiacaba mountain range, Brazil | Atlantic tropical rainforest | Primata, Carnivora, Rodentia, Cingulata, Didelphimorphia, Pilosa (15 spp) | settlement | subsistence | illegal | 1 | Species abundance (n/10 km or n tracks/station) was estimated using track plots and line transects. Three areas with different intensity of hunting pressure were surveyed (Sede and Turvinho base in Carlos Botelho State Park and Zizo’s Park Particular Reserve. Hunting frequency was assessed via interviews to hunters and via direct observations. Sampling effort was 80.2 km in Sede, 74.2 km in Turvinho and 70.2 km in Zizo (total: 225.1 km).  .Control distance: 6 km |
| Poulsen et al., 2011 | SP  [100] | Kabo Forest Reserve, D. R. Congo | tropical forest | Cetartiodactyla, Primates, Rodentia, Proboscidea (19 spp) | settlement | subsistence & commercial | legal | s1 | Species densities (n/km2) were calculated using line transects in three forest types: logged and hunted forest, logged and unhunted forest, and unlogged and unhunted forest. On the first two are considered in the meta-analysis as hunted and unhunted areas. In each forest type 10 2.5-km transects were randomly located and surveyed during two years. The transects follow a gradient of hunting intensity from the town of Kabo. DISTANCE was used to estimate densities. Distances were calculated with GIS. Control distance: 33 km. |
| Remis, 2000 | SP [101] | Dzanga-Sangha Forest Reserve (DSFR), Central African Republic | tropical rainforest | Cetartiodactyla, Primata, Proboscidea (3 species and 2 pooled) | settlement | subsistence & commercial | legal and illegal | 1 | Species abundances (signs/km, calls/km, dungs/km, nests/km) were estimated using line transect sampling in three 30-sq-km sample blocks located at varying distances (10, 30 and 35 km) from the population centre of Bayanga. Three trained research teams surveyed six variable width and length (4-5 km) line transects in each block for wildlife and human sign (survey effort: 81.2 km).Gorilla and chimpanzee tracks, food remains or dung, elephant and ungulate dung, and monkey sightings and calls were recorded on each transect. All indications of human activity, hunting sings and presence were noted. DISTANCE was used to calculate gorilla density estimates. Control distance: 30 km (the site with lowest hunting signs/km) |
| Remis and Kpanou 2010 | SP  [102] | Dzanga-Sangha Reserve, Central African Republic | tropical rainforest | Cetartiodactyla, Primata, Proboscidea (12 spp, 4 duiker spp. pooled in one) | road & Settlement | subsistence & commercial | legal and illegal, depending on the site | 1 | Encounter rates (signs/5 km) were calculated using line transects in 4 areas with different levels of hunting pressure and at varying distances from the main town. At each site 5-km linear transects were placed perpendicular to the primary logging roads and the  drainage patterns to sample a range of vegetation gradients (Core Park, three sites 20 km each = 60 km, Core Reserve, three sites 20 km each = 60 km, Remote Park, one site, two 5 km transects = 10 km, Remote Reserve, one site, five 5 km transects = 25 km).Hunting signs were recorded per site, and the core park area is used as control due to low hunting pressure. Distances to roads and settlements calculated using GIS. Control distance: 6.35 km. |
| Reyna-Hurtado, 2002; Reyna-Hurtado and Tanner, 2007 | MT, SP  [103, 104] | Calakmul Biosphere Reserve and buffer areas, Mexico | tropical rainforest | Cetartiodactyla, Perissodactyla (5 spp) | settlement | subsistence | legal | 1 | Straight line transects were cut in the forest and used to estimate the relative abundance of ungulate species (track encounter rate, n/km) between Feb- Jul 2001. Transects were randomly located in three ejidos previously identified as hunting areas (N = 24, 20, 18 transects) and in a non-hunted area (CBR, N =28 transects). Transects started 3-5 km away from the villages to avoid censusing in fragmented areas due to agriculture. Abundance data is pooled for the three hunted sites and compared to the abundance in the control area. Control distance > 20 km from the nearest hunted site and 10 km from road. |
| Roldan and Simonetti, 2001 | SP  [105] | Beni Biosphere Reserve (Estación Biológica Beni,  EBB), Bolivia | lowland tropical forests | Carnivora, Rodentia, Cingulata, Perissodactyla, Cetartiodactyla (9 spp) | settlement | subsistence | legal | 1 | Species relative abundance (n plots with tracks/total plots) was estimated using track plots in two sites located 30 km apart. The intensely hunted forest was located 1 km from a Tsimane village, whereas the other forest was occasionally hunted and rarely visited by Tsimane hunters. 100 plots of 0.5 m2 were established evenly spaced along a 500-m transect within each forest, and tracks were counted every month (January-June 1996). Total sampling effort was 703 and 850 plot-days for the occasionally hunted forest and the  intensively hunted forest, respectively. Control distance: 30 km |
| Rosenbaum et al., 1998 | SP  [106] | Tangkoko-Batuangas-DuaSudara Nature Reserve,  Sulawesi, Indonesia | lowland tropical rainforest | Primata (1 sp) | settlement | subsistence  & commercial | illegal | 1 | Species densities (n/km2) were estimated along five trails at TBDS between 1002-1994. Two sites were used for comparison, Tangkoko as control (2 trails) and DuaSudara as hunted forest (1 trail). Batuangas (2 trails) is a disturbed forest and was excluded from further analyses. Distances to settlements calculated with GIS. Control distance >4 km. |
| Rosin and Swamy, 2013 | SP  [107] | Madre de Dios river basin, Peru | tropical rainforest | Primates (5 spp) | settlement | subsistence | legal | 1 | Encounter rates (n/10 km) were estimated using line transects of varying length in three sites (RA: 4 transects, 100.15 km; LA: 3 transects, 102.3 km; TRC: 3 transects, 102.5 km) under varying degrees of protection from hunting pressure. Transects were surveyed in the morning and in the afternoon. The size of the focal protected area and straight-line distance to the nearest human settlement were used as proxies for hunting pressure. The sites were selected to minimize potentially confounding natural variation and other effects of such as logging and agriculture. Control distance > 50 km. |
| Rovero et al., 2012 | SP  [108] | Udzungwa Mountains, South-Central Tanzania | lowland deciduous forest and evergreen moist montane forest | Primata(6 spp) | settlement | subsistence & commercial | illegal | 1 | Primate encounter rates (groups/km) were estimated using line-transects between Feb 2004 and Oct 2009 in a hunted (USFR) and unhunted (MW) forest in in the Udzungwa Mountains. Three 3.1–4 km long transects were laid out in each forest and 180 and 134 transect repetitions were conducted in MW and USFR, totalling 702 and 479 km of transect, respectively. Hunting signs were recorded and hunters were interviewed. Hunting villages located at 2.5-5 km from hunted forest. Control distance: 150 km |
| Salvador, 2015 | MT  [109] | Yasuní National Park, Ecuador | evergreen terra firme tropical forest | Cetartiodactyla, Rodentia, Perissodactyla  (7 spp) | road | subsistence & commercial | not specified | 1 | Species abundance (n photos/100 days) was estimated using camera traps. 60 camera trap sites were spatially distributed in 24 transects (ranging from 0.4 to 5 km long) perpendicular to both sides of the Maxus Road. Cameras were located approximately 100 m off human-made trails, so as not to bias records in favor of species that preferentially use such trails. Photo capture rates of each of the studied species were recorded within different distance categories from Maxus Road: 400 – 1000 m, 1000 – 2000 m, 2000 – 3000 m, and 3000 – 4700 m. Control distance: 3.85 km |
| Schmid and Rasoloarison, 2002 | BC  [110] | Ankarafantsika National Park, N Madagascar | xerophytic forests and scrubs | Primata (5 spp) | road | subsistence | illegal | 1 | Species abundance (sightings/km) was estimated at different sites located at different distances from a national road. The most remote site was relatively intact. Distance to roads was obtained from (Kun-Rodrigues et al., 2014). At each site, 2-3 line transects were surveyed 2-10 times using nocturnal and diurnal censuses, depending on species activity. Local people were questioned to collect information about the presence of lemur species and about the hunting pressure on the primate fauna. Control distance: 17.6 km |
| Shaffer et al., 2017a,b | SP  [111, 112] | Konashen Community Owned Conservation Area (KCOCA), Guyana | tropical rainforest | Primata (1 sp) | settlement | subsistence | legal | 1 | Species abundance (n/10 km) was estimated from line-transect surveys conducted in the KCOCA in 2013 and 2015. Two 10 km transects radiating out from Masakenari were used to conduct surveys using standard line-transect survey methodology. Hunting effort was recorded and a biodemographic model applied and validated with the empirically-derived encounter rates. Control distance: 10 km |
| Stokes et al., 2010 | SP  [113] | N Congo | tropical rainforest | Primata & Proboscidea (3 spp) | road & settlement | commercial | illegal | 1 | Line transect distance sampling was used to estimate densities (ind/km2) of elephants, gorillas and chimpanzees from counts of elephant dung piles and great ape sleeping nests respectively. 10 different teams performed the counts from February to August 2006 in the dry season. Every transect was walked by 2 to 3 different teams and all were done as fast as possible before moving to the next one in order to minimize seasonal variation. In total 166 transects were walked with a total distance of 329.7km. The GPS location of every observation was recorded in order to relate this with distance to roads and settlements. Control distance: 22-33km. |
| Suarez et al.,2013 | SP  [114] | Yasuní Biosphere Reserve, Ecuador | tropical rainforest (terra firme) | Primates, Cetartiodactyla, Rodentia (21 spp) | road & settlement | subsistence and commercial | legal and illegal | 1 | Species density was estimated from bi-monthly surveys of medium-sized and large mammals between April 2005 to July 2006. Surveys were performed using distance sampling techniques along six line transects (2 km each) in each of three sampling sites (1 control and two hunted sites). Each transect originated and continued perpendicularly from the roads (Taracoa and Yasuní Research Station) or from the river (Control site). Each transect was surveyed during eight time periods. Sample size per site: 6 x 8 = 48. Hunted distances: up to 2 km, but could expand further into the forest (Control distance: 89 (Taracoa) and 40 km (Yasuni) |
| de Thoisy et al., 2005 | SP  [115] | N French Guiana | tropical rainforest | Primata (6 spp) | settlement | subsistence & commercial | illegal | 1 | Study performed in 17 sites spread all over the country. The sighting rates (indiv/km) of 6 monkey species were estimated using 1 line transect of 4-5km at every of the 13 hunted sites and 4 unhunted sites. 1 site was recently logged (<5 years ago). Every transect was walked 20-38 times. Most hunting occurs within 2.5 km of access points (settlements, tracks and rivers). Maximum distance in hunting trip is 5 km. Values of unhunted sites used as control densities (sighting rates). Control distance: 5 km. |
| Topp-Jørgensen et al. 2009 | SP  [116] | Uzungwa mountains, Tanzania | montane forest | Primates, Cetartiodactyla, Proboscidea, Tubulidentata, Rodentia (11 spp) | settlement | subsistence | illegal | 1 | Relative densities (groups/km, animal trails/km and number burrows/ha) were estimated using line transects, line intersect methods and fixed area searches, depending on the surveyed species. Surveyed line transects were 4.42 km, 4.26 km, and 6.00 km in WKSFR (22 repetitions), USFR (19 repetitions) and NDUFR (10 repetitions). For the line intersect method, transects were 4.26, 4.09 and 4.65 km long. Trails were attributed to a species based on footprints and dung. In the fixed area searches, the number of burrows within five meters to either side of the transect was recorded. Only active burrows were recorded based on uncovered trails and holes. A distance of 25 m between entrance holes was applied to discern between den systems to avoid overestimation. Distances were calculated with GIS. Control distance: > 7.5 km |
| Trolliet et al., 2017 | SP  [117] | Malebo Research station, Bandundu province, D.R. Congo | semi-natural forest-savanna mosaic | Cetartiodactyla, Primata, Proboscidea (4 spp) | settlement | subsistence | legal | 1 | Species abundance (observations/km) was estimated by surveying line-transects and REConnaissanCE (RECCE) transects, totaling 131 km across five sites with different hunting pressure. All direct and indirect traces of the presence of animals (feces, footprints, calls, nests) and hunting activity (rifle cartridges, gunshots, traps, fires) were recorded. Only two sites used for this analysis (Mbanzi and Nkombo) because the others are small fragments. Control distance: 8.75 km |
| Urquiza-Haas et al. 2011 | SP  [118] | Yucatán Peninsula, Mexico | tropical forest | Primata, Rodentia, Cetartiodactyla, Carnivora, Pilosa (9 spp) | settlement | subsistence | Not specified | 2 (2 control sites according to forest cover) | Diurnal vertebrate surveys were conducted using standardized line-transect census. Species were identified by direct sightings and/or acoustic cues, particularly alarm calls. Two or three transects of 3–4.5km in length were walked once a day at each site. Cumulative effort was 93.3–147.4km per survey site, and a total of 927.2km walked across all eight sites. Distance intervals not clear, but hunters reported exploiting catchment areas between 2.5 and 9 km of their settlements, and typically within 3–4 km. Hunting activities were evaluated qualitatively via semi-structured interviews. Control distance > 9 km. |
| Van Vliet and Nasi, 2008 | SP  [119] | SW Gabon | tropical rainforest | Primates, Cetartiodactyla & Proboscidea (12 spp) | road | subsistence | legal | 1 | Species observations were recorded during a routine forest inventory between 2001 and 2003. 159 parallel line transects of the same distance were used with 5711 survey units centered on the transect lines of 20m by 200m each. Hunting signs were recorded as well. The distance of each survey unit to the nearest road was recorded. Almost no sign of hunting traces over 7 km. Data aggregated over 0-3, 3-7,7-10 and >10 km distance intervals. Control distance: >10 km. |
| Vinitpornsawan, 2013 | DT  [120] | Thung Yai Naresuan Wildlife Sanctuary, Thailand | tropical rainforest | Carnivora, Proboscidea, Perissodactyla, Cetartiodactyla (8 spp) | settlement | subsistence & commercial | illegal | 2 (sign survey and camera trap survey) | Mean relative abundance of mammal species was estimated in a sign survey covering 10 16x16 km grid cells (Nov. 2010-Feb 2012). Length of survey routes within grid cells were proportional to the amount of forest cover (max= 60 km per 256 km2). Tracks, pellet/dung piles/scat, scratch marks, direct sight, etc. were recorded only the first encounter of each type of sign/evidence within each 100 m segment. Mean relative abundance (n 100m segments with sign/total number) is calculated for 1 km grid cells at varying distances to settlements. Human disturbance index is also recorded. Camera trap was used for tiger and leopard (50 cameras, 99 camera trap locations, 1689 trap nights). No sign of tiger or leopards within 10 km from villages. Mean density estimates for these two species were obtained using Bayesian approaches. Assumptions about control distance (longest). Control distance: 10 km and >10 km. |
| Wang et al., 2007 | SP  [121] | Dja Reserve and Kompia Community forest, S Cameroon | tropical rainforest (44% mature semi-deciduous lowland forest, abandoned fields/secondary forests (20%), swamps (26%) and active crop plots (10%) | Primates (6 spp) | settlement | subsistence & commercial | legal | 1 | Arboreal frugivores were censused using modified line transects in 1999 and 2004. At the hunted forest site (16.3 km2 centered around Kompia village), frugivores were surveyed on four routes (4.4-5.8 km long). At the protected forest site (25 km2), surveys were conducted on seven routes (6.4 -7.9 km long). All routes were surveyed three times per month. Total length: 640 km in the hunted (N= 4 routes x 3 repeats = 12 repeats per month) and 1727 km in protected forest sites (N= 7 routes x 3 repeats = 21 repeats per month). Mean monthly relative abundance estimates and confidence intervals were obtained for each primate species at each site. Monthly estimates were pooled to obtain a unique estimate per species per site. Control distance: 23 km |
| Weber, 2005 | DT  [122] | Calakmul Biosphere Reserve and buffer area, Mexico | tropical rainforest | Cetartiodactyla (3 spp) | settlement | subsistence | legal | 1 | Deer density estimates (tracks/km) were obtained using line transect sampling on 6 permanent transects and 23 temporary transects in areas with no hunting, moderate and heavy hunting pressure. Mean hunting distance: 13.5 km. Control distance located in reserve in isolated area: 60 km (although 10 km from other nearest village). |
| Wilkie et al., 1987, 1990 | DT, SP  [123, 124] | Ituri Forest, E D.R. Congo | tropical rainforest | Cetartiodactyla, Proboscidea, Carnivora, Primata, Pholidota & Rodentia (17 spp) | road & settlements | subsistence | legal | 1 | Using line transects, the average number of tracks was calculated in 3 5-ha sites within mature forest (unhunted, located 6km from the nearest road) and 3 5-ha sites in secondary forest (hunting occurs between 1.5 and 3km from the road). In each study site five track recording stations were equally placed along each of five 100m transects. Data was collected from June 1982 to January 1983 and during April 1983. Every station was checked every 7 to 11 days. Control distance: 6 km. |
| Williams-Guillen et al., 2006 | SP, TR  [125, 126] | Bosawás Biosphere Reserve, Nicaragua | tropical rainforest | Primata, Cetartiodactyla, Rodentia, Cingulata, Carnivora, Perissodactyla (16 spp) | settlement | subsistence | legal | 1 | Species abundance (n/km) was estimated using 14 1-km line transects randomly located in areas with different hunting pressure and at increasing distance from the village. Transects were surveyed several times between Oct 2003 – Dec 2004. All direct and indirect observations (sightings, tracks, faeces, …) were annotated. Total sampling effort was 151 km. Control distance: 25 km |
| Wright et al., 2000 | SP  [127] | Several sites,  Panama | Secondary lowland tropical rainforests (evergreen and wet, age: 80-150 y) | Cetartiodactyla, Didelphimorphia, Carnivores, Primates, Pilosa, Rodentia (11 spp) | settlements | subsistence & commercial (not clearly reported) | illegal | 1 | Abundance is reported as number individuals/km2, as number of groups/km (primates) and as number animal captured/100 trapping nights. 5-km transects were established in eight study sites with different hunting pressures and different levels of protection. Transect counts were conducted weekly between 18 Aug – 11 Dec 1997 (total 634 km transects; 60-89 km per site). Additionally two rounds of small mammal trapping were conducted at each site using Sherman traps. Poaching intensitity was estimated by recording hunting signs in each site and by interviews with national park guards. Distances were calculated with GIS and georreferenced transects. Control distance: 9 km |
| Yasuoka 2006 | SP  [128] | Site adjacent to Nki and Boumba-Bek National Parks, Cameroon | tropical rainforest | Cetartiodactyla, Proboscidea (5 spp, red duikers pooled) | settlement (along logging road) | subsistence | legal | 1 | Species density (n/km2) and relative abundance (number of dungs/plot) was estimated in February and March 2005 at varying distances from a hunting village (Zoualabot), after the bushmeat trade boom during 2002-2004.Two 20×500-m belt transects were established in five plots at 13, 17, 22, 28 and 33 km from the village. When animal dung was found in the belt transect, the distance along the transect, the perpendicular distance from the centerline, the name of the animal, and the date of defecation estimated by the Baka, were recorded. Dung density was calculated based on the assumption that all dung in the belt transect was counted, and was not prone to underestimation. Hunting signs (snares) were recorded at each plot. Control distance: 33 km. |

**References**

1. Aliaga-Rossel E. The cascading effect of mammal species defaunation on seed and seedling survivorship as a result of hunting: University of Hawaii at Manoa; 2011.

2. Altrichter M. The sustainability of subsistence hunting of peccaries in the Argentine Chaco. Biological Conservation. 2005;126(3):351-62.

3. Aquino R, López L, Arévalo I, García G, Charpentier E. Densidad de ungulados en bosques de baja y alta presión de caza en el nororiente de la Amazonía peruana. Ciencia Amazónica (Iquitos). 2014;4(2):128-37.

4. Atickem A, Loe LE, Langangen O, Rueness EK, Bekele A, Stenseth NC. Estimating population size and habitat suitability for mountain nyala in areas with different protection status. Animal Conservation. 2011;14(4):409-18. doi: 10.1111/j.1469-1795.2011.00437.x. PubMed PMID: WOS:000293173900014.

5. Averbeck C, Plath M, Wronski T, Apio A. Effect of human nuisance on the social organisation of large mammals: group sizes and compositions of seven ungulate species in Lake Mburo National Park and the adjacent Ankole Ranching Scheme. Wildlife Biology. 2012;18(2):180-93.

6. Barlow J, Peres CA. Effects of single and recurrent wildfires on fruit production and large vertebrate abundance in a central Amazonian forest. Biodiversity and Conservation. 2006;15(3):985-1012.

7. Barnes RFW, Barnes KL, Alers MPT, Blom A. Man determines the distribution of elephants in the rain forests of northeastern Gabon. African Journal of Ecology. 1991;29(1):54-63. doi: 10.1111/j.1365-2028.1991.tb00820.x.

8. Barrera Zambrano VA, Zambrano Moncada J, Stevenson PR. Diversity of regenerating plants and seed dispersal in two canopy trees from Colombian Amazon forests with different hunting pressure. Revista De Biologia Tropical. 2008;56(3):1531-42. PubMed PMID: WOS:000265268000042.

9. Baur EH. Estudio de la cacería de subsistencia en la Concesión Forestal de Carmelita, San Andrés, Petén. Informe interno. Guatemala: ProPetén/Conservation International, 1999.

10. Blake S, Strindberg S, Boudjan P, Makombo C, Bila-Isia I, Ilambu O, et al. Forest elephant crisis in the Congo Basin. Plos Biology. 2007;5(4):945-53. doi: 10.1371/journal.pbio.0050111. PubMed PMID: WOS:000245901500025.

11. Blake J, Mosquera D, Salvador J. Use of mineral licks by mammals and birds in hunted and non‐hunted areas of Yasuní National Park, Ecuador. Animal Conservation. 2013;16(4):430-7.

12. Blom A, Van Zalinge R, Mbea E, Heitkönig I, Prins HH. Human impact on wildlife populations within a protected Central African forest. African Journal of Ecology. 2004;42(1):23-31.

13. Blom A, van Zalinge R, Heitkonig IMA, Prins HHT. Factors influencing the distribution of large mammals within a protected central African forest. Oryx. 2005;39(4):381-8. doi: 10.1017/s0030605305001080. PubMed PMID: WOS:000234153500009.

14. Bodmer RE, Eisenberg JF, Redford KH. Hunting and the Likelihood of Extinction of Amazonian Mammals: Caza y Probabilidad de Extinción de Mamiferos Amazónicos. Conservation Biology. 1997;11(2):460-6.

15. Bowkett AE, Rovero F, Marshall AR. The use of camera-trap data to model habitat use by antelope species in the Udzungwa Mountain forests, Tanzania. African Journal of Ecology. 2008;46(4):479-87. doi: 10.1111/j.1365-2028.2007.00881.x. PubMed PMID: WOS:000261243500004.

16. Briceño-Méndez M, Naranjo E, Mandujano S, Altricher M, Reyna-Hurtado R. Responses of two sympatric species of peccaries (Tayassu pecari and Pecari tajacu) to hunting in Calakmul, Mexico. Tropical Conservation Science. 2016;9(3):1940082916667331.

17. Brodie JF, Helmy OE, Brockelman WY, Maron JL. Bushmeat poaching reduces the seed dispersal and population growth rate of a mammal-dispersed tree. Ecological Applications. 2009;19(4):854-63. doi: doi:10.1890/08-0955.1.

18. Cabassu Y. Impacts of indigenous subsistence hunting on wildlife abundance in the Río Plátano Biosphere Reserve, Honduras [MSc thesis]. Ottawa, Ontario, Canada: Carleton University; 2010.

19. Calderón-Quiñonez AP. Efectos de la cacería sobre la abundancia de mamíferos y sus consecuencias en la herbivoría y pisoteo de plántulas en tres áreas protegidas de Panamá [MSc thesis]. Nueva Guatemala de la Asunción: Universidad San Carlos de Guatemala; 2010.

20. Caro TM. Densities of mammals in partially protected areas: the Katavi ecosystem of western Tanzania. Journal of Applied Ecology. 1999;36(2):205-17. doi: 10.1046/j.1365-2664.1999.00392.x. PubMed PMID: WOS:000081084900002.

21. Carrillo E, Wong G, Cuarón AD. Monitoring mammal populations in Costa Rican protected areas under different hunting restrictions. Conservation Biology. 2000;14(6):1580-91.

22. Chiarello AG. Effects of fragmentation of the Atlantic forest on mammal communities in south-eastern Brazil. Biological Conservation. 1999;89(1):71-82.

23. Croes B, Funston P, Rasmussen G, Buij R, Saleh A, Tumenta P, et al. The impact of trophy hunting on lions (Panthera leo) and other large carnivores in the Bénoué Complex, northern Cameroon. Biological Conservation. 2011;144(12):3064-72.

24. Cronin DT. The impact of bushmeat hunting on the primates of Bioko Island, Equatorial Guinea [PhD Thesis]. Philadelphia, PA: Drexel University; 2013.

25. Cruz P, Paviolo A, Bó RF, Thompson JJ, Di Bitetti MS. Daily activity patterns and habitat use of the lowland tapir (Tapirus terrestris) in the Atlantic Forest. Mammalian Biology-Zeitschrift für Säugetierkunde. 2014;79(6):376-83.

26. Cullen L, Bodmer RE, Pádua CV. Effects of hunting in habitat fragments of the Atlantic forests, Brazil. Biological conservation. 2000;95(1):49-56.

27. Cullen L, Bodmer E, Valladares-Padua C. Ecological consequences of hunting in Atlantic forest patches, São Paulo, Brazil. Oryx. 2001;35(02):137-44.

28. Danquah E. Spatial Distribution of Elephants versus Human and Ecological Variables in Western Ghana. Advances in Ecology. 2016;2016.

29. Davies A, Schulte-Herbrüggen B, Kümpel NF, Mendelson S. Hunting and trapping in Gola Forests, south-eastern Sierra Leone: Bushmeat from farm, fallow and forest. Bushmeat and Livelihoods: Wildlife Management and Poverty Reduction Blackwells Publishing, Oxford. 2007:15-29.

30. de Andrade Melo ER, Gadelha JR, Domingos da Silva MdN, da Silva Junior AP, Mendes Pontes AR. Diversity, abundance and the impact of hunting on large mammals in two contrasting forest sites in northern amazon. Wildlife Biology. 2015;21(5):234-45. doi: 10.2981/wlb.00095. PubMed PMID: WOS:000363668300001.

31. Demmer J, Godoy R, Wilkie D, Overman H, Taimur M, Fernando K, et al. Do levels of income explain differences in game abundance? An empirical test in two Honduran villages. Biodiversity and Conservation. 2002;11(10):1845-68. doi: 10.1023/a:1020305903156. PubMed PMID: WOS:000178204300010.

32. Derby AM. Investigating how ecology and demography influence folivorous primate biomass in the Western Amazon [PhD Thesis]. Stony Brook, NY: Stony Brook University; 2008.

33. Dethier M. Etude chasse. Yaounde, Cameroon: ECOFAC, 1995.

34. Doherty DA. Hunting and the implications for mammals in Belize [PhD thesis]. Davis, CA: University of California Davis; 2005.

35. Effiom EO, Nuñez-Iturri G, Smith HG, Ottosson U, Olsson O. Bushmeat hunting changes regeneration of African rainforests. Proceedings of the Royal Society of London B: Biological Sciences. 2013;280(1759):20130246.

36. Effiom EO, Birkhofer K, Smith HG, Olsson O. Changes of community composition at multiple trophic levels due to hunting in Nigerian tropical forests. Ecography. 2014;37(4):367-77.

37. Emmons LH. Geographic variation in densities and diversities of non-flying mammals in Amazonia. Biotropica. 1984:210-22.

38. Endo W, Peres CA, Salas E, Mori S, Sanchez-Vega JL, Shepard GH, et al. Game Vertebrate Densities in Hunted and Nonhunted Forest Sites in Manu National Park, Peru. Biotropica. 2010;42(2):251-61. doi: 10.1111/j.1744-7429.2009.00546.x. PubMed PMID: WOS:000275215700017.

39. Espinosa-Andrade SR. Road development, bushmeat extraction and jaguar conservation in Yasuni Biosphere Reserve-Ecuador [PhD thesis]. Gainesville, FL: University of Florida; 2012.

40. Espinosa S, Celis G, Branch LC. When roads appear jaguars decline: Increased access to an Amazonian wilderness area reduces potential for jaguar conservation. PloS one. 2018;13(1):e0189740.

41. Eves HE. The bushmeat crisis in Central Africa: resolving a common pool resource problem in the common interest [PhD thesis]. New Haven, CT: Yale University; 2006.

42. Fay JM, Agnagna M. A population survey of forest elephants (Loxodonta africana cyclotis) in Northern Congo. African Journal of Ecology. 1991;29(3):177-87.

43. Fay JM. An elephant (Loxodonta africana) survey using dung counts in the forests of the Central African Republic. Journal of Tropical Ecology. 1991;7(01):25-36.

44. Fimbel C, Curran B, Usongo L. Enhancing the sustainability of duiker hunting through community participation and controlled access in the Lobéké region of southeastern Cameroon. In: Robinson JG, Bennett EL, editors. Hunting for sustainability in tropical forests. New York: Columbia University Press; 2000. p. 356-74.

45. Fragoso JM. The effect of hunting on tapirs in Belize. In: Robinson JG, Redford KH, editors. Neotropical wildlife use and conservation. Chicago, IL: Chicago University Press; 1991. p. 154-62.

46. Glanz WE. Mammalian densities at protected versus hunted sites in Central Panama. In: Robinson JG, Redford KH, editors. Neotropical wildlife use and conservation Chicago, IL: Chicago University Press; 1991. p. 163-73.

47. Gray TNE, Phan C. Habitat preferences and activity patterns of the larger mammal community in Phnom Prich Wildlife Sanctuary, Cambodia. Raffles Bulletin of Zoology. 2011;59(2):311-8. PubMed PMID: WOS:000301312200022.

48. Hall JS, Inogwabini BI, Williamson EA, Omari I, Sikubwabo C, White LJT. A survey of elephants (Loxodonta africana) in the Kahuzi-Biega National Park lowland sector and adjacent forest in eastern Zaire. African Journal of Ecology. 1997;35(3):213-23. doi: 10.1111/j.1365-2028.1997.088-89088.x. PubMed PMID: WOS:000072183400004.

49. Hall JS, White LJ, Inogwabini B-I, Omari I, Morland HS, Williamson EA, et al. Survey of Grauer's gorillas (Gorilla gorilla graueri) and eastern chimpanzees (Pan troglodytes schweinfurthi) in the Kahuzi-Biega National Park lowland sector and adjacent forest in eastern Democratic Republic of Congo. International Journal of Primatology. 1998;19(2):207-35.

50. Hart J. Impact and sustainability of indigeneous hunting in the Ituri forest, Congo-Zaire: a comparison of unhunted and hunted duiker populations. In: Robinson JG, Bennett EL, editors. Hunting for sustainability in tropical forests. New York: Columbia University Press; 2000. p. 106-53.

51. Hayward MW. Bushmeat hunting in Dwesa and Cwebe Nature Reserves, Eastern Cape, South Africa. South African Journal of Wildlife Research. 2009;39(1):70-84. doi: 10.3957/056.039.0108. PubMed PMID: WOS:000269692700008.

52. Hegerl C, Burgess ND, Nielsen MR, Martin E, Ciolli M, Rovero F. Using camera trap data to assess the impact of bushmeat hunting on forest mammals in Tanzania. Oryx. 2015:1-11.

53. Hema EM, Barnes RFW, Guenda W. Distribution of savannah elephants (Loxodonta africana africana Blumenbach 1797) within Nazinga game ranch, Southern Burkina Faso. African Journal of Ecology. 2011;49(2):141-9. doi: 10.1111/j.1365-2028.2010.01239.x. PubMed PMID: WOS:000290171900002.

54. Henschel P, Hunter LTB, Coad L, Abernethy KA, Muehlenberg M. Leopard prey choice in the Congo Basin rainforest suggests exploitative competition with human bushmeat hunters. Journal of Zoology. 2011;285(1):11-20. doi: 10.1111/j.1469-7998.2011.00826.x. PubMed PMID: WOS:000294170500002.

55. Henschel P. The conservation biology of the leopard Panthera pardus in Gabon: status, threats and strategies for conservation [PhD Thesis]. Göttingen, Germany: University of Göttingen; 2008.

56. Hill K, Padwe J, Bejyvagi C, Bepurangi A, Jakugi F, Tykuarangi R, et al. Impact of hunting on large vertebrates in the Mbaracayu Reserve, Paraguay. Conservation Biology. 1997;11(6):1339-53.

57. Hurtado-Gonzales JL, Bodmer RE. Assessing the sustainability of brocket deer hunting in the Tamshiyacu-Tahuayo Communal Reserve, northeastern Peru. Biological Conservation. 2004;116(1):1-7.

58. Takayoshi K, Ryu A. Hunting Pressure on Chimpanzees and Gorillas in the Motaba River Area, Northeastern Congo. African Study Monographs. 1994;15(3):143-62.

59. Koerner SE, Poulsen JR, Blanchard EJ, Okouyi J, Clark CJ. Vertebrate community composition and diversity declines along a defaunation gradient radiating from rural villages in Gabon. Journal of Applied Ecology. 2016.

60. Kosydar AJ, Rumiz DI, Conquest LL, Tewksbury JJ. Effects of hunting and fragmentation on terrestrial mammals in the Chiquitano forests of Bolivia. Tropical Conservation Science. 2014;7(2):288-307. doi: 10.1017/s0376892906002566. PubMed PMID: WOS:000238311500006.

61. Kuehl HS, Nzeingui C, Yeno SLD, Huijbregts B, Boesch C, Walsh PD. Discriminating between village and commercial hunting of apes. Biological Conservation. 2009;142(7):1500-6. doi: 10.1016/j.biocon.2009.02.032. PubMed PMID: WOS:000266752500026.

62. Kumpel NF, Milner-Gulland EJ, Rowcliffe JM, Cowlishaw G. Impact of gun-hunting on diurnal primates in continental Equatorial Guinea. International Journal of Primatology. 2008;29(4):1065-82. doi: 10.1007/s10764-008-9254-9. PubMed PMID: WOS:000258959800015.

63. Kun-Rodrigues C, Salmona J, Besolo A, Rasolondraibe E, Rabarivola C, Marques TA, et al. New Density Estimates of a Threatened Sifaka Species ( Propithecus coquereli) in Ankarafantsika National Park. American Journal of Primatology. 2014;76(6):515-28. doi: 10.1002/ajp.22243. PubMed PMID: WOS:000335490200001.

64. Lahm SA. Ecology and economics of human/wildlife interaction in northeastern Gabon [PhD thesis]. New York: New York University; 1993.

65. Lahm S, Barnes R, Beardsley K, Cervinka P. A method for censusing the greater white-nosed monkey in northeastern Gabon using the population density gradient in relation to roads. Journal of Tropical Ecology. 1998;14(05):629-43.

66. Laurance WF, Croes BM, Tchignoumba L, Lahm SA, Alonso A, Lee ME, et al. Impacts of roads and hunting on central African rainforest mammals. Conservation Biology. 2006;20(4):1251-61. doi: 10.1111/j.1523-1739.2006.00420.x. PubMed PMID: WOS:000239545500036.

67. Levi T, Shepard GH, Jr., Ohl-Schacherer J, Wilmers CC, Peres CA, Yu DW. Spatial tools for modeling the sustainability of subsistence hunting in tropical forests. Ecological Applications. 2011;21(5):1802-18. PubMed PMID: WOS:000292766100029.

68. Mitchell CL, Raez-Luna EF. The impact of human hunting on primate and game bird populations in the Manu Biosphere Reserve in Southeastern Peru. New York, USA: Wildlife Conservation Society, New York Zoological Society, 1991.

69. Lin L, Feng L, Pan W, Guo X, Zhao J, Luo A, et al. Habitat selection and the change in distribution of Asian elephants in Mengyang Protected Area, Yunnan, China. Acta Theriologica. 2008;53(4):365-74. doi: 10.1007/bf03195197. PubMed PMID: WOS:000260556500005.

70. Linder JM, Oates JF. Differential impact of bushmeat hunting on monkey species and implications for primate conservation in Korup National Park, Cameroon. Biological Conservation. 2011;144(2):738-45. doi: <http://dx.doi.org/10.1016/j.biocon.2010.10.023>.

71. Linder JM. The impact of hunting on primates in Korup National Park, Cameroon: implications for primate conservation [PhD thesis]. New York, NY: City University of New York; 2008.

72. Lwanga JS. The influence of forest variation and possible effects of poaching on duiker abundance at Ngogo, Kibale National Park, Uganda. African Journal of Ecology. 2006;44(2):209-18.

73. Madhusudan M, Karanth K. Hunting for an answer: is local hunting compatible with large mammal conservation in India. Hunting for sustainability in tropical forests. 2000;1:339-55.

74. Maldonado Rodriguez AM. The Impact of Subsistence Hunting by Tikunas on Game Species in Amacayacu National Park, Colombian Amazon [PhD thesis]. Oxford, UK: Oxford Brookes University; 2010.

75. Marshall AR, Topp-Jørgensen JE, Brink H, Fanning E. Monkey Abundance and Social Structure in Two High-Elevation Forest Reserves in the Udzungwa Mountains of Tanzania. International Journal of Primatology. 2005;26(1):127-45. doi: 10.1007/s10764-005-0011-z.

76. Marshall AJ, Nardiyono, Engstrom LM, Pamungkas B, Palapa J, Meijaard E, et al. The blowgun is mightier than the chainsaw in determining population density of Bornean orangutans (Pongo pygmaeus morio) in the forests of East Kalimantan. Biological Conservation. 2006;129(4):566-78. doi: 10.1016/j.bibcon.2005.11.025. PubMed PMID: WOS:000237878900014.

77. Martins E. A caça de subsistencia de extrativistas na Amazonia: sustentabilidade, biodiversidade e extinçao de especies [MSc thesis]. Brasilia, Brazil: Universidade de Brasilia; 1992.

78. Mena V, Stallings J, Regalado J, Cueva R. The sustainability of current hunting practices by the Huaorani. In: Robinson JG, Bennett EL, editors. Hunting for sustainability in tropical forests. New York: Columbia University Press; 2000. p. 57-78.

79. Muchaal PK, Ngandjui G. Impact of village hunting on wildlife populations in the western Dia Reserve, Cameroon. Conservation Biology. 1999;13(2):385-96. doi: 10.1046/j.1523-1739.1999.013002385.x. PubMed PMID: WOS:000079472000022.

80. Nielsen MR. Importance, cause and effect of bushmeat hunting in the Udzungwa Mountains, Tanzania: Implications for community based wildlife management. Biological Conservation. 2006;128(4):509-16. doi: 10.1016/j.biocon.2005.10.017. PubMed PMID: WOS:000236100800009.

81. Nijman V. Effects of habitat disturbance and hunting on the density and the biomass of the endemic Hose's leaf monkey Presbytis hosei (Thomas, 1889) (Mammalia : Primates : Cercopithecidae) in east Borneo. Contributions to Zoology. 2004;73(4):283-91. PubMed PMID: WOS:000227928800004.

82. Noss AJ. Cable snares and nets in the Central African Republic. Hunting for sustainability in tropical forests. New York: Columbia University Press; 2000. p. 282-304.

83. Noss A. La sostenibilidad de la cacería de subsistencia Izoceña. In: Cabrera E, Mercolli C, Resquin R, editors. Manejo de fauna silvestre en Amazonía y Latinoamérica: CITES (Convención sobre el Comercio Internacional); 2000. p. 535-44.

84. Novack AJ. Impacts of subsistence hunting on the foraging ecology of the jaguar and puma in the Maya Biosphere Reserve, Guatemala [MSc thesis]. Gainesville, FL: University of Florida; 2003.

85. Nuñez-Iturri G, Olsson O, Howe HF. Hunting reduces recruitment of primate-dispersed trees in Amazonian Peru. Biological Conservation. 2008;141(6):1536-46.

86. Nuñez-Iturri G, Howe HF. Bushmeat and the Fate of Trees with Seeds Dispersed by Large Primates in a Lowland Rain Forest in Western Amazonia. Biotropica. 2007;39(3):348-54. doi: 10.1111/j.1744-7429.2007.00276.x.

87. Nuñez-Iturri G. The effects of hunting on the regeneration of trees in mature floodplain forests in southeastern Peru [PhD Thesis]. Champaign, IL: University of Illinois; 2007.

88. Paciulli LM. The effects of logging, hunting, and vegetation on the densities of the Pagai, Mentawai Island Primates [PhD thesis]. Stony Brook, NY: Stony Brook University; 2004.

89. Parry L, Barlow J, Peres CA. Hunting for Sustainability in Tropical Secondary Forests. Conservation Biology. 2009;23(5):1270-80. doi: 10.1111/j.1523-1739.2009.01224.x. PubMed PMID: WOS:000269802200030.

90. Paviolo A, De Angelo C, Di Blanco Y, Agostini I, Pizzio E, Melzew R, et al. Efecto de la caza y el nivel de protección en la abundancia de los grandes mamíferos del Bosque Atlántico de Misiones. Contribuciones para la conservación y manejo en el Parque Nacional Iguazú. 2009:237-54.

91. Paviolo AJ. Densidad de yaguareté (*Panthera onca*) en la selva paranaense : su relación con la disponibilidad de presas, presión de caza y coexistencia con el puma (*Puma concolor*) [PhD thesis]. Córdoba, Argentina: UNIVERSIDAD NACIONAL DE CÓRDOBA; 2010.

92. Perera-Romero L, Polisar J, Maffei L. Grandes vertebrados terrestres en tierras ancestrales indígenas del alto Caura: importancia de acuerdos comunitarios para la conservación del Escudo Guayanés venezolano. In: Payán E, Lasso CA, Castaño-Uribe C, editors. I Conservación de grandes vertebrados en áreas no protegidas de Colombia, Venezuela y Brasil Serie Editorial Fauna Silvestre Neotropical. Bogotá, D.C., Colombia: Instituto de Investigación de Recursos Biológicos Alexander von Humboldt (IAvH). 2015.

93. Peres CA, Lake IR. Extent of nontimber resource extraction in tropical forests: Accessibility to game vertebrates by hunters in the Amazon basin. Conservation Biology. 2003;17(2):521-35. doi: 10.1046/j.1523-1739.2003.01413.x. PubMed PMID: WOS:000181736400021.

94. Peres CA, Nascimento HS. Impact of game hunting by the Kayapo of south-eastern Amazonia: implications for wildlife conservation in tropical forest indigenous reserves. Biodiversity and Conservation. 2006;15(8):2627-53. doi: 10.1007/s10531-005-5406-9. PubMed PMID: WOS:000241168900016.

95. Peres CA. Effects of hunting on western Amazonian primate communities. Biological Conservation. 1990;54(1):47-59. doi: <http://dx.doi.org/10.1016/0006-3207(90)90041-M>.

96. Peres CA. Population status of white-lipped *Tayassu pecari* and collared peccaries *T. tajacu* in hunted and unhunted Amazonian forests. Biological Conservation. 1996;77(2-3):115-23. doi: 10.1016/0006-3207(96)00010-9. PubMed PMID: WOS:A1996UT59400001.

97. Pia MV. Influencia conjunta de la vegetación, asentamientos humanos, caminos y actividades ganaderas sobre la ocurrencia y dieta de los carnívoros tope de Achala (Córdoba, Argentina) [PhD thesis]. Córdoba, Argentina: Universidad Nacional de Córdoba; 2011.

98. Pia MV, Renison D, Mangeaud A, De Angelo C, Haro JG. Occurrence of top carnivores in relation to land protection status, human settlements and rock outcrops in the high mountains of central Argentina. Journal of Arid Environments. 2013;91:31-7. doi: 10.1016/j.jaridenv.2012.11.004. PubMed PMID: WOS:000318056100005.

99. Pianca CC. A caça e seus efeitos sobre a ocorrência de mamíferos de médio e grande porte em áreas preservadas de Mata Atlântica na Serra de Paranapiacaba (SP). [MSc thesis]. Sao Paulo, Brazil: Universidade de Sao Paulo; 2004.

100. Poulsen J, Clark C, Bolker B. Decoupling the effects of logging and hunting on an Afrotropical animal community. Ecological Applications. 2011;21(5):1819-36.

101. Remis MJ. Preliminary assessment of the impacts of human activities on gorillas Gorilla gorilla gorilla and other wildlife at Dzanga‐Sangha Reserve, Central African Republic. Oryx. 2000;34(1):56-65.

102. Remis MJ, Kpanou JB. Primate and ungulate abundance in response to multi‐use zoning and human extractive activities in a Central African Reserve. African Journal of Ecology. 2011;49(1):70-80.

103. Reyna-Hurtado R, Tanner GW. Ungulate relative abundance in hunted and non-hunted sites in Calakmul Forest (Southern Mexico). Biodiversity and Conservation. 2007;16(3):743-56. doi: 10.1007/s10531-005-6198-7.

104. Reyna-Hurtado RÁ. Hunting effects on the ungulate species in Calakmul forest, Mexico [MSc thesis]. Gainesville, FL: University of Florida; 2002.

105. Roldán AI, Simonetti JA. Plant‐mammal interactions in tropical Bolivian forests with different hunting pressures. Conservation Biology. 2001;15(3):617-23.

106. Rosenbaum B, O'Brien TG, Kinnaird M, Supriatna J. Population densities of Sulawesi crested black macaques (Macaca nigra) on Bacan and Sulawesi, Indonesia: effects of habitat disturbance and hunting. American Journal of Primatology. 1998;44(2):89-106.

107. Rosin C, Swamy V. Variable Density Responses of Primate Communities to Hunting Pressure in a Western Amazonian River Basin. Neotropical Primates. 2013;20(1):25-31. doi: 10.1896/044.020.0105.

108. Rovero F, Mtui AS, Kitegile AS, Nielsen MR. Hunting or habitat degradation? Decline of primate populations in Udzungwa Mountains, Tanzania: An analysis of threats. Biological Conservation. 2012;146(1):89-96.

109. Salvador J. Effects of human disturbance on occurrence patterns and behavior of terrestrial mammals in eastern Ecuador [MSc thesis]. Florida, USA: University of Florida; 2015.

110. Schmid J, Rasoloarison MR. Lemurs of the Réserve Naturelle d’Ankarafantsika, Madagascar. In: Alonso LE, Schulenberg TS, Radilofe S, Missa O, editors. A biological assessment of the Réserve Naturelle Intégrale d’Ankarafantsika, Madagascar RAP Bulletin of Biological Assessment No 23. Washington, DC: Conservation International; 2002. p. 73-82.

111. Shaffer CA, Milstein MS, Yukuma C, Marawanaru E, Suse P. Sustainability and comanagement of subsistence hunting in an indigenous reserve in Guyana. Conservation Biology. 2017;31(5):1119-31.

112. Shaffer C, Yukuma C, Marawanaru E, Suse P. Assessing the sustainability of Waiwai subsistence hunting in Guyana by comparison of static indices and spatially explicit, biodemographic models. Animal Conservation. 2018;21(2):148-58.

113. Stokes EJ, Strindberg S, Bakabana PC, Elkan PW, Iyenguet FC, Madzoke B, et al. Monitoring Great Ape and Elephant Abundance at Large Spatial Scales: Measuring Effectiveness of a Conservation Landscape. Plos One. 2010;5(4). doi: 10.1371/journal.pone.0010294. PubMed PMID: WOS:000277079300006.

114. Suarez E, Zapata-Rios G, Utreras V, Strindberg S, Vargas J. Controlling access to oil roads protects forest cover, but not wildlife communities: a case study from the rainforest of Yasuni Biosphere Reserve (Ecuador). Animal Conservation. 2013;16(3):265-74. doi: 10.1111/j.1469-1795.2012.00592.x. PubMed PMID: WOS:000319703600005.

115. de Thoisy B, Renoux F, Julliot C. Hunting in northern French Guiana and its impact on primate communities. Oryx. 2005;39(2):149-57. doi: 10.1017/s0030605305000384. PubMed PMID: WOS:000228944800017.

116. Topp-Jørgensen E, Nielsen MR, Marshall AR, Pedersen U. Relative densities of mammals in response to different levels of bushmeat hunting in the Udzungwa Mountains, Tanzania. Tropical Conservation Science. 2009;2(1):70-87.

117. Trolliet F, Forget PM, Huynen MC, Hambuckers A. Forest cover, hunting pressure, and fruit availability influence seed dispersal in a forest‐savanna mosaic in the Congo Basin. Biotropica. 2017;49(3):337-45.

118. Urquiza-Haas T, Peres CA, Dolman PM. Large vertebrate responses to forest cover and hunting pressure in communal landholdings and protected areas of the Yucatan Peninsula, Mexico. Animal Conservation. 2011;14(3):271-82. doi: 10.1111/j.1469-1795.2010.00426.x.

119. Van Vliet N, Nasi R. Mammal distribution in a Central African logging concession area. Biodiversity and Conservation. 2008;17(5):1241-9. doi: 10.1007/s10531-007-9300-5. PubMed PMID: WOS:000255998100017.

120. Vinitpornsawan S. Population and spatial ecology of Tigers and Leopards relative to prey availability and human activity in Thung Yai Naresuan (East) Wildlife Sanctuary, Thailand [PhD Thesis]. Amherts, MA: University of Massachussets Amherts; 2013.

121. Wang BC, Sork VL, Leong MT, Smith TB. Hunting of mammals reduces seed removal and dispersal of the afrotropical tree Antrocaryon klaineanum (Anacardiaceae). Biotropica. 2007;39(3):340-7.

122. Weber M. Ecology and conservation of sympatric tropical deer populations in the Greater Calakmul Region, south-eastern Mexico [PhD Thesis]. Durham, UK: Durham University; 2005.

123. Wilkie DS, Finn JT. Slash-burn cultivation and mammal abundance in the Ituri Forest, Zaire. Biotropica. 1990:90-9.

124. Wilkie DS. Impact of swidden agriculture and subsistence hunting on diversity and abundance of exploited fauna in the Ituri forest of NorthEastern Zaire: University of Massachusetts; 1987.

125. Williams-Guillén K, Griffith D, Polisar J, Camilo G, Bauman K. Abundancia de animales de caza y características de cacería en el territorio indígena de Kipla Sait Tasbaika, reserva de biósfera BOSAWAS. Wani. 2006;23:37-61.

126. William-Guillen K, Griffith D, Polisar J, Dixon O, Camilo G, Asa C, et al. Poblaciones de animales silvestres y sostenabilidad de la cacería en Kipla Sait Tasbaika Kum, Bosawás, Nicaragua. Unpublished Report, Saint Louis Zoo, Managua, Nicaragua. 2006.

127. Wright SJ, Zeballos H, Domínguez I, Gallardo MM, Moreno MC, Ibáñez R. Poachers alter mammal abundance, seed dispersal, and seed predation in a Neotropical forest. Conservation Biology. 2000;14(1):227-39.

128. Yasuoka H. The sustainability of duiker (Cephalophus spp.) hunting for the Baka hunter-gatherers in southeastern Cameroon. African Study Monographs. 2006;33:95-120.
