# Supplementary material for: Intact but empty forests? Patterns of hunting-induced mammal defaunation in the tropics
Source: PLoS Biol. 2019 May 14;17(5):e3000247. doi: 10.1371/journal.pbio.3000247 (PMC6516652; doi:10.1371/journal.pbio.3000247)
Supplement: S2 Table — (DOCX) [file pbio.3000247.s014.docx]

**Table S2**. Overview of explanatory variables included in the hurdle models.

| **Explanatory variable** | **Expected relationship** | **Reason for inclusion** | **Type, transformation** | **Source** | **Resolution** | **Notes** |
| --- | --- | --- | --- | --- | --- | --- |
| Distance to hunters’ access points (km) | Non-linear positive, saturating beyond a certain threshold | Distance to hunter’s access points is negatively related with hunting pressure. Therefore, species abundance increases with distance to hunters’ access points and levels off beyond a certain threshold distance. | Continuous, log | Reported in the studies or, if not readily available, by georeferencing the study location and calculating the distances to the closest settlement in ArcGIS. | NA |  |
| Travel Time to major towns (min) | Non-linear positive, saturating beyond a certain threshold | Travel time to major towns is used as a *proxy* of accessibility to urban markets. Hunting pressure is expected to be higher in areas more accessible (with less travel time) to urban markets. | Continuous, log | Nelson, A. (2008) Estimated travel time to the nearest city of 50,000 or more people in year 2000. Global Environment Monitoring Unit - Joint Research Centre of the European Commission, Ispra Italy. Available at http://forobs.jrc.ec.europa.eu/products/gam/ (accessed 28/01/2016).[129]  D. Weiss *et al.*, A global map of travel time to cities to assess inequalities in accessibility in 2015. *Nature*, (2018)[130] | 30” |  |
| Region | Higher impacts in historically more hunted regions, such as SE Asia and Africa, compared to C America and S America | Hunting-induced defaunation may vary regionally. For example, regions with more human population and historically more hunted (Africa) may be more defaunated (lower effect sizes) than regions with historically less human population (South America). | Categorical, 4 levels  (Africa, C America, S America and Asia) | Study | NA |  |
| Diet | Abundance declines are expected to be larger in carnivores and herbivores as they are largely threatened by hunting | Large-scale hunting may affect feeding guilds differently, with different consequences for ecosystem functioning. Different guild can respond to hunting differently depending on their population density, gregariousness, locomotion habits, resource availability, etc | Categorical, 5 levels (frugivore, carnivore, herbivore, insectivore, omnivore) | EltonTraits 1.0 database (Wilman, H., Belmaker, J., Simpson, J., de la Rosa, C., Rivadeneira, M.M., Jetz, W., 2014. EltonTraits 1.0: Species-level foraging attributes of the world's birds and mammals. Ecology 95, 2027-2027) [131] | NA |  |
| Body Mass (kg) | Non-linear negative, saturating beyond a certain threshold | Large species are more heavily hunted. | Continuous, log | EltonTraits 1.0 database (Wilman, H., Belmaker, J., Simpson, J., de la Rosa, C., Rivadeneira, M.M., Jetz, W., 2014. EltonTraits 1.0: Species-level foraging attributes of the world's birds and mammals. Ecology 95, 2027-2027) [131] | NA |  |
| Protected areas | Lower species abundance outside protected areas | The impact of hunting on wildlife populations is expect to be larger outside of protected areas under the most stringent IUCN categories (I-IV) | Categorical, 2 levels (inside, outside) | Study/GIS IUCN WDPA ([www.protectedplanet.org](http://www.protectedplanet.org)) | NA |  |
| Stunting in children < 5 years old (%) | Non-linear positive or negative, saturating beyond a certain threshold | Stunting is intimately linked to poverty levels. If bushmeat is an inferior good, we expect higher hunting pressure in poorer areas (high stunting prevalence). On the contrary, if bushmeat is a normal good, we expect higher hunting pressure in wealthier areas (low stunting prevalence) | Continuous, percentage | FAO (Food and Agriculture Organization), 2003. Chronic under nutrition among children: an indicator of poverty. Poster and unpublished data set. FAO, Rome. [www.fao.org](http://www.fao.org)  Updated with data from WHO Global Health Observatory (http://www.who.int/gho/database/en/). | 30’ | When data was not available we used data from the nearest grid cells/countries. E.g.: For French Guiana we used the average of the closest grid cells from Surinam and Brazil. |
| Livestock biomass (kg/km^2^) | Non-linear positive,  saturating beyond a certain threshold | Accessibility to alternative protein sources may shift wild meat dependence towards domestic consumption, leading to lower hunting pressure. | Continuous, log | Robinson, T. P., Wint, G. W., Conchedda, G., Van Boeckel, T. P., Ercoli, V., Palamara, E., ... & Gilbert, M. (2014). Mapping the global distribution of livestock. PloS one, 9(5), e96084.[132] | 30” | Calculated based on livestock densities of cattle, sheep, goat, chicken and pigs, and by multiplying n/km^2^ by average weight per livestock type (Leonie Lautz, unpublished). |
| Literacy rate in adults (%) | Non-linear positive,  saturating beyond a certain threshold | Education has been shown to positively correlate with environmental health and with the potential to access labor market. We thus expected literacy rate to be negatively related to hunting pressure, and positively to mammal abundances. | Continuous, percentage | United Nations Educational, Scientific, and Cultural Organization (UNESCO). Institute for statistics. <https://data.worldbank.org/indicator/SE.ADT.LITR.ZS> | Country level |  |
