# Supplementary material for: Intact but empty forests? Patterns of hunting-induced mammal defaunation in the tropics
Source: PLoS Biol. 2019 May 14;17(5):e3000247. doi: 10.1371/journal.pbio.3000247 (PMC6516652; doi:10.1371/journal.pbio.3000247)
Supplement: S3 Table — (DOCX) [file pbio.3000247.s015.docx]

**Table S3**. Sources of settlement location data.

| **Country** | **Year** | **Source** | **Link** | **Comments** |
| --- | --- | --- | --- | --- |
| Angola | 2017 | OpenStreetMap | <http://download.geofabrik.de/africa/angola.html> |  |
| Argentina | 2015, 2017 | Instituto Geografico Nacional (Argentina), OpenStreetMap | [http://www.ign.gob.ar/sig#descarga, http://download.geofabrik.de/south-america/argentina.html](http://www.ign.gob.ar/sig#descarga) | Datasets were merged. |
| Bangladesh | 2017 | OpenStreetMap | <http://download.geofabrik.de/asia/bangladesh.html> |  |
| Belize | 2010, 2017 | Meerman, J. and Clabaugh, J. 2017. Biodiversity and Environmental Resource Data System of Belize. Online. http://www.biodiversity.bz, OpenStreetMap | [http://www.biodiversity.bz/; http://download.geofabrik.de/central-america/belize.html](http://www.biodiversity.bz/;) | Datasets were merged. |
| Benin | 2017 | OpenStreetMap | <http://download.geofabrik.de/africa/benin.html> |  |
| Bolivia | 2001, 2017 | Instituto Nacional de Estadísticas and OCHA Regional Office for Latin America and the Caribbean, OpenStreetMap | [https://data.humdata.org/dataset/bolivia-settlements; http://download.geofabrik.de/south-america/bolivia.html](https://data.humdata.org/dataset/bolivia-settlements) | Datasets were merged. |
| Bostwana | 2017 | Bostwana | <http://download.geofabrik.de/africa/botswana.html> |  |
| Brazil | 2010, 2017 | Instituto Brasileiro de Geografia e Estatística, OpenStreetMap | http://downloads.ibge.gov.br/downloads_geociencias.htm; http://download.geofabrik.de/south-america/brazil.html | Datasets were merged. |
| Brunei | 2017 | OpenStreetMap | <http://download.geofabrik.de/asia/malaysia-singapore-brunei.html> |  |
| Burkina Faso | 2017 | OpenStreetMap | <http://download.geofabrik.de/africa/burkina-faso.html> |  |
| Burundi | 2017 | OpenStreetMap | <http://download.geofabrik.de/africa/burundi.html> |  |
| Cambodia | 2017 | OpenStreetMap | <http://download.geofabrik.de/asia/cambodia.html> |  |
| Cameroon | 2012, 2017 | Interactive Forest Atlas of Cameroon, World Resources Institute (WRI) and the Ministry of Forestry and Wildlife (MINFOF), OpenStreetMap | [http://www.wri.org/our-work/project/congo-basin-forests/cameroon; http://download.geofabrik.de/africa/cameroon.html](http://www.wri.org/our-work/project/congo-basin-forests/cameroon) | Datasets were merged. |
| Central African Republic | 2012, 2017 | Interactive Forest Atlas of Central African Republic, World Resources Institute (WRI) and the Ministry of Waters, Forests, Hunting and Fishing (MEFCP), OpenStreetMap | [http://www.wri.org/our-work/project/congo-basin-forests/central-african-republic#project-tabs, http://download.geofabrik.de/africa/central-african-republic.html](http://www.wri.org/our-work/project/congo-basin-forests/central-african-republic#project-tabs) | Datasets were merged. |
| Chad | 2017 | OpenStreetMap | <http://download.geofabrik.de/africa/chad.html> |  |
| China | 2017 | OpenStreetMap | http://download.geofabrik.de/asia/china.html |  |
| Colombia | 2008, 2017 | DANE, División Político Administrativa de Colombia; OpenStreetMap | [https://blog.jorgeivanmeza.com/2008/09/departamentos-y-municipios-de-colombia-actualizacion-20080915/; http://download.geofabrik.de/south-america/colombia.html](https://blog.jorgeivanmeza.com/2008/09/departamentos-y-municipios-de-colombia-actualizacion-20080915/) | Datasets were merged. |
| Congo | 2010, 2017 | Forest Atlas of Congo, World Resources Institute (WRI) and the Ministry of Forest Economy and Sustainable Development (MEFDD), OpenStreetMap | [http://www.wri.org/our-work/project/congo-basin-forests/congo#project-tabs, http://download.geofabrik.de/africa/congo-brazzaville.html](http://www.wri.org/our-work/project/congo-basin-forests/congo#project-tabs) | Datasets were merged. |
| Costa Rica | 2016 | Portal de Datos Abiertos "Daticos" | <http://daticos-geotec.opendata.arcgis.com/datasets/f3c43f4bcf884657aea5683e2041ebea_0> |  |
| Cote d'Ivoire | 2016, 2017 | Humanitarian Data Exchange, OCHA-CI and the Comité National de Télédétection et d'Information Géographique (CNTIG), OpenStreetMap | [https://data.humdata.org/dataset/444d352f-db9f-49bc-b170-37d31e9a1433,http://download.geofabrik.de/africa/ivory-coast.html](https://data.humdata.org/dataset/444d352f-db9f-49bc-b170-37d31e9a1433,http:/download.geofabrik.de/africa/ivory-coast.html) | Datasets were merged. |
| D.R. Congo | 2013, 2017 | Interactive Forest Atlas of the Democratic Republic of Congo, World Resources Institute (WRI) and Ministry of Environment & Sustainable Development (MEDD); OpenStreetMap | [http://www.wri.org/our-work/project/congo-basin-forests/democratic-republic-congo#project-tabs, http://download.geofabrik.de/africa/congo-democratic-republic.html](http://www.wri.org/our-work/project/congo-basin-forests/democratic-republic-congo#project-tabs) | Datasets were merged. |
| Ecuador | 2012 | Infraestructura de Datos Espaciales para Instituto Geográfico Militar | <http://www.geoportaligm.gob.ec/portal/index.php/descargas/cartografia-de-libre-acceso/registro/> |  |
| Equatorial Guinea | 2015, 2017 | Humanitarian Data Exchange, OCHA Regional Office for West and Central Africa, National Geospatial-Intelligence Agency (NGA), OpenStreetMap | [https://data.humdata.org/dataset/equatorial-guinea-settlements, http://download.geofabrik.de/africa/equatorial-guinea.html](https://data.humdata.org/dataset/equatorial-guinea-settlements) | Datasets were merged. |
| Ethiopia | 2016 | Humanitarian Data Exchange, OCHA Ethiopia | <https://data.humdata.org/dataset/ethiopia-settlements> |  |
| French Guiana | 2009 | BRGM (Bureau de Recherches Géologiques et Minières) | <http://gisguyane.brgm.fr/gis_download.asp?langue=GB&theme=GRA> |  |
| Gabon | 2013, 2017 | Interactive Forest Atlas of Gabon, World Resources Institute (WRI) and Ministry of Forest Economy, Water, Fishing, and Aquaculture (MEFEPA), OpenStreetMap | [http://www.wri.org/our-work/project/congo-basin-forests/gabon#project-tabs, http://download.geofabrik.de/africa/gabon.html](http://www.wri.org/our-work/project/congo-basin-forests/gabon#project-tabs) | Datasets were merged. |
| Gambia | 2017 | OpenStreetMap | <http://download.geofabrik.de/africa/senegal-and-gambia.html> |  |
| Ghana | 2017 | OpenStreetMap | <http://download.geofabrik.de/africa/ghana.html> |  |
| Guatemala | NA | Secretaria Planificacion y Ordenacion (SEGEPLAN) | <http://www.segeplan.gob.gt/nportal/index.php/ide-descargas> |  |
| Guinea | 2016, 2017 | Humanitarian Data Exchange, OCHA Regional Office for West and Central Africa, National Geospatial-Intelligence Agency (NGA), OpenStreetMap | [https://data.humdata.org/dataset/guinea-settlement, http://download.geofabrik.de/africa/guinea.html](https://data.humdata.org/dataset/guinea-settlement) | Datasets were merged. |
| Guinea-Bissau | 2017 | OpenStreetMap | <http://download.geofabrik.de/africa/guinea-bissau.html> |  |
| Guyana | 2000 | Center for International Earth Science Information Network - CIESIN - Columbia University, International Food Policy Research Institute - IFPRI, The World Bank, and Centro Internacional de Agricultura Tropical - CIAT. 2011. Global Rural-Urban Mapping Project, Version 1 (GRUMPv1): Settlement Points. Palisades, NY: NASA Socioeconomic Data and Applications Center (SEDAC). | <http://sedac.ciesin.columbia.edu/data/set/grump-v1-settlement-points/data-download> |  |
| Honduras | 2001 | GIS portal Honduras government | <http://www.sinit.hn/> |  |
| India | 2017 | OpenStreetMap | http://download.geofabrik.de/asia/india.html |  |
| Indonesia | 2017 | OpenStreetMap | <http://download.geofabrik.de/asia/indonesia.html> |  |
| Kenya | 2017 | OpenStreetMap | <http://download.geofabrik.de/africa/kenya.html> |  |
| Lao | 2017 | Humanitarian Data Exchange,Lao Statistics Bureau (LSB), OCHA ROAP | <https://data.humdata.org/dataset/laos-populated-places> |  |
| Lesotho | 2017 | OpenStreetMap | <http://download.geofabrik.de/africa/lesotho.html> |  |
| Liberia | 2015, 2017 | Humanitarian Data Exchange, OCHA Liberia, United Nations Mission in Liberia (UNMIL) and Humanitarian Information Centre (HIC), OpenStreetMap | [https://data.humdata.org/dataset/liberia-settlements, http://download.geofabrik.de/africa/liberia.html](https://data.humdata.org/dataset/liberia-settlements) | Datasets were merged. |
| Madagascar | 2017 | OpenStreetMap | <http://download.geofabrik.de/africa/madagascar.html> |  |
| Malaysia | 2017 | OpenStreetMap | <http://download.geofabrik.de/asia/malaysia-singapore-brunei.html> |  |
| Mali | 2015, 2017 | Humanitarian Data Exchange, Direction Nationale de l'Administration Territoriales (DNAT) and l'Institut national de la statistique (INSTAT), OCHA Mali, OpenStreetMap | [https://data.humdata.org/dataset/mali-settlements, http://download.geofabrik.de/africa/mali.html](https://data.humdata.org/dataset/mali-settlements) | Datasets were merged. |
| Mexico | 2016 | Intituto Nacional de Estadistica y Geografia (INEGI) | <http://www.beta.inegi.org.mx/app/biblioteca/ficha.html?upc=702825217341> |  |
| Mozambique | 2017, 2017 | Humanitarian Data Exchange, International Organization for Migration (IOM), OpenStreetMap | [https://data.humdata.org/dataset/mozambique-settlement-shapefiles, http://download.geofabrik.de/africa/mozambique.html](https://data.humdata.org/dataset/mozambique-settlement-shapefiles) | Datasets were merged. |
| Myanmar | 2017, 2017 | Humanitarian Data Exchange, MIMU, UNODC, WFP, OCHA Myanmar, OpenStreetMap | [https://data.humdata.org/dataset/mmr-settlement, http://download.geofabrik.de/asia/myanmar.html](https://data.humdata.org/dataset/mmr-settlement) | Datasets were merged. |
| Namibia | 2017 | OpenStreetMap | <http://download.geofabrik.de/africa/namibia.html> |  |
| Nicaragua | 2016 | OpenStreetMap | <http://datos.mapanica.net/index.html> |  |
| Niger | 2016, 2017 | Humanitarian Data Exchange, OCHA Niger and Institut National de la Statistique, OpenStreetMap | [https://data.humdata.org/dataset/niger-settlements, http://download.geofabrik.de/africa/niger.html](https://data.humdata.org/dataset/niger-settlements) | Datasets were merged. |
| Nigeria | 2017 | OpenStreetMap | http://download.geofabrik.de/africa/nigeria.html |  |
| Panama | 2010 | Censo Nacional de Población y Vivienda 2010 Panama | <http://datos-geored.opendata.arcgis.com/datasets/46931f3d6c794ab7adbfa5825f1a2f34_0> |  |
| Paraguay | 2017 | OpenStreetMap | <http://download.geofabrik.de/south-america/paraguay.html> |  |
| Peru | 2016, 2017 | Ministerio de Educacion de Peru, OpenStreetMap | [http://sigmed.minedu.gob.pe/descargas/, http://download.geofabrik.de/south-america/peru.html](http://sigmed.minedu.gob.pe/descargas/) | Datasets were merged. |
| Philippines | 2017 | OpenStreetMap | <http://download.geofabrik.de/asia/philippines.html> |  |
| Rwanda | 2017 | OpenStreetMap | <http://download.geofabrik.de/africa/rwanda.html> |  |
| Senegal | 2017, 2017 | Humanitarian Data Exchange, Government of Senegal, OCHA ROWCA, OpenStreetMap | [https://data.humdata.org/dataset/senegal-settlements, http://download.geofabrik.de/africa/senegal-and-gambia.html](https://data.humdata.org/dataset/senegal-settlements) | Datasets were merged. |
| Sierra Leone | 2017 | OpenStreetMap | <http://download.geofabrik.de/africa/sierra-leone.html> |  |
| Singapore | 2017 | OpenStreetMap | <http://download.geofabrik.de/asia/malaysia-singapore-brunei.html> |  |
| Somalia | 2017 | OpenStreetMap | <http://download.geofabrik.de/africa/somalia.html> |  |
| South Africa | 2017 | OpenStreetMap | <http://download.geofabrik.de/africa/south-africa.html> |  |
| South Sudan | 2017 | OpenStreetMap | http://download.geofabrik.de/africa/south-sudan.html |  |
| Sri Lanka | 2017 | OpenStreetMap | http://download.geofabrik.de/asia/sri-lanka.html |  |
| Sudan | 2017 | OpenStreetMap | <http://download.geofabrik.de/africa/sudan.html> |  |
| Suriname | 2017 | OpenStreetMap | <http://download.geofabrik.de/south-america/suriname.html> |  |
| Swaziland | 2017 | OpenStreetMap | <http://download.geofabrik.de/africa/swaziland.html> |  |
| Tanzania | 2017 | OpenStreetMap | <http://download.geofabrik.de/africa/tanzania.html> |  |
| Thailand | 2017 | OpenStreetMap | <http://download.geofabrik.de/asia/thailand.html> |  |
| Togo | 2017 | OpenStreetMap | <http://download.geofabrik.de/africa/togo.html> |  |
| Uganda | 2015, 2017 | Humanitarian Data Exchange, Uganda Bureau of Statistics (UBOS), OCHA ROSEA, OpenStreetMap | [https://data.humdata.org/dataset/uganda-other-0-0; http://download.geofabrik.de/africa/uganda.html](https://data.humdata.org/dataset/uganda-other-0-0) | Datasets were merged. |
| Uruguay | 2017 | OpenStreetMap | http://download.geofabrik.de/south-america/uruguay.html |  |
| Venezuela | 2015 | Carlos Efraín Porto Tapiquén. Orogénesis Soluciones Geográficas. Porlamar, Venezuela, 2015. | [http://tapiquen-sig.jimdo.com](http://tapiquen-sig.jimdo.com/) |  |
| Vietnam | 2015, 2017 | Humanitarian Data Exchange, Open Street Map, OCHA ROAP, OpenStreetMap | [https://data.humdata.org/dataset/viet-nam-settlements; http://download.geofabrik.de/asia/vietnam.html](https://data.humdata.org/dataset/viet-nam-settlements) | Datasets were merged. |
| Zambia | 2017 | OpenStreetMap | <http://download.geofabrik.de/africa/zambia.html> |  |
| Zimbabwe | 2015 | Humanitarian Data Exchange, Zimbabwe Department of the Surveyor General (DSG), OCHA ROSA, OpenStreetMap | [https://data.humdata.org/dataset/zimbabwe-settlements; http://download.geofabrik.de/africa/zimbabwe.html](https://data.humdata.org/dataset/zimbabwe-settlements) | Datasets were merged. |
