# Supplementary material for: Intact but empty forests? Patterns of hunting-induced mammal defaunation in the tropics
Source: PLoS Biol. 2019 May 14;17(5):e3000247. doi: 10.1371/journal.pbio.3000247 (PMC6516652; doi:10.1371/journal.pbio.3000247)
Supplement: S4 Table — (DOCX) [file pbio.3000247.s016.docx]

**Table S4**. Number of animals and studies used to estimate average body weights for cattle, sheep, goats, pigs and chicken.

| **Livestock category** | **Number studies** | **Number individuals** | **Body weight (mean ± SD)** | **Sources** |
| --- | --- | --- | --- | --- |
| Cattle | 29 | 1531 | 495.6 ± 106.2 | [133-160] |
| Sheep and goats | 37 | 4951 | 54.9 ± 6.9 | [139, 144, 161-195] |
| Pigs | 24 | 2378 | 117.5 ± 25.4 | [134, 139, 164, 196-216] |
| Chicken | 32 | 2036 | 2.112 ± 0.41 | [217-248] |
