# Supplementary material for: Intact but empty forests? Patterns of hunting-induced mammal defaunation in the tropics
Source: PLoS Biol. 2019 May 14;17(5):e3000247. doi: 10.1371/journal.pbio.3000247 (PMC6516652; doi:10.1371/journal.pbio.3000247)
Supplement: S5 Table — Models were ranked according to BIC. We only show models with a BIC weight >0.01. The best model (ΔBIC < 2, in bold) was used in the cross-validation analyses and for spatial predictions. BM, body mass; BIC, Bayesian Information Criterion; Dist, distance to hunters’ access points, PA, protected area; PopDens, human population density; Stunt, stunting; TravTime, travel time to major towns. (DOCX) [file pbio.3000247.s017.docx]

**Table S5.** Model selection results for (a) the binomial model (0/1, extirpated vs no extirpated) and (b) the continuous model. Models were ranked according to BIC. We only show models with a BIC weight > 0.01. The best model (ΔBIC < 2, in bold) was used in the cross-validation analyses and for spatial predictions. BM: Body mass, Dist: Distance to hunters’ access points, PopDens: Human population density, Stunt: Stunting, PA: Protected area, TravTime: Travel time to major towns.

1. Binomial model

| Variables | df | logLik | BIC | ΔBIC |
| --- | --- | --- | --- | --- |
| **BM + Dist + Dist^2^ + PopDens + Stunt + PA** | **10** | **-928.64** | **1937.96** | **0.00** |
| BM + Dist + Dist^2^ + Stunt + PA | 9 | -934.16 | 1940.92 | 2.97 |
| BM + Dist + PopDens + Stunt + PA | 9 | -935.44 | 1943.50 | 5.54 |
| BM + Dist + Dist^2^ + LivestockBio + PopDens + Stunt + PA | 11 | -927.78 | 1944.30 | 6.35 |
| BM + Dist + Dist^2^ + Stunt + PA + TravTime | 10 | -931.94 | 1944.56 | 6.6 |
| BM + Dist + Dist^2^ + PopDens + PopDens2 + Stunt + PA | 11 | -928.09 | 1944.92 | 6.97 |
| BM + Dist + Dist^2^ + PopDens + Stunt + PA + TravTime | 11 | -928.14 | 1945.03 | 7.07 |
| BM + Dist + Dist^2^ + PA | 8 | -940.6 | 1945.74 | 7.79 |
| BM + Dist + Dist^2^ + PopDens + Stunt + Stunt^2^ + PA | 11 | -928.58 | 1945.91 | 7.95 |
| BM + BM^2^ + Dist + Dist^2^ + PopDens + Stunt + PA | 11 | -928.64 | 1946.02 | 8.06 |
| BM + Dist + Dist^2^ + BM x Dist + PopDens + Stunt + PA | 11 | -928.64 | 1946.02 | 8.07 |

1. Continuous model

| Variables | df | logLik | BIC | ΔBIC | |
| --- | --- | --- | --- | --- | --- |
| **BM + Dist + Dist^2^ + PopDens + PopDens^2^ + BM x Dist** | **11** | **-3701.45** | **7490.20** | **0.00** |  |
| BM + Dist + Dist^2^ + PopDens + PopDens^2^ + Stunt + BM x Dist | 12 | -3698.56 | 7492.34 | 2.14 |  |
| BM + Dist + Dist^2^ + PopDens + PopDens^2^ + PA + BM x Dist | 12 | -3699.84 | 7494.92 | 4.72 |  |
| BM + Dist + Dist^2^ + PopDens + PopDens^2^ + BM x Dist + LivestockBio | 12 | -3700.35 | 7495.94 | 5.74 |  |
| BM + Dist + Dist^2^ + PopDens + PopDens^2^ + BM x Dist + LivestockBio + LivestockBio^2^ | 13 | -3696.42 | 7496.01 | 5.81 |  |
| BM + Dist + Dist^2^ + PopDens + PopDens^2^ + Stunt + PA + BM x Dist | 13 | -3696.83 | 7496.82 | 6.62 |  |
| BM + BM^2^ + Dist + Dist^2^ + PopDens + PopDens^2^ + BM x Dist | 12 | -3700.92 | 7497.07 | 6.87 |  |
| BM + Dist + Dist^2^ + BM x Dist | 9 | -3712.86 | 7497.15 | 6.95 |  |
| BM + Dist + Dist^2^ + PopDens + PopDens^2^ + BM x Dist + LivestockBio + LivestockBio^2^ + Stunt | 14 | -3693. 21 | 7497.53 | 7.33 |  |
| BM + Dist + Dist^2^ + PopDens + PopDens^2^ + TravTime + BM x Dist | 12 | -3701.43 | 7498.09 | 7.89 |  |
| BM + Dist + Dist^2^ + PopDens + PopDens^2^ + BM x Dist + LivestockBio + Stunt | 13 | -3697.78 | 7498.73 | 8.53 |  |
| BM + BM^2^ + Dist + Dist^2^ + PopDens + PopDens^2^ + Stunt + BM x Dist | 13 | -3698.07 | 7499.32 | 9.12 |  |
| BM + Dist + PopDens + PopDens^2^ + BM x Dist | 10 | -3709.99 | 7499.35 | 9.15 |  |
